# Supplementary figures and images for: Genome-wide identification and classification of the Hsf and sHsp gene families in Prunus mume, and transcriptional analysis under heat stress
Source: PeerJ. 2019 Jul 29;7:e7312. doi: 10.7717/peerj.7312 (PMC6673427; doi:10.7717/peerj.7312)

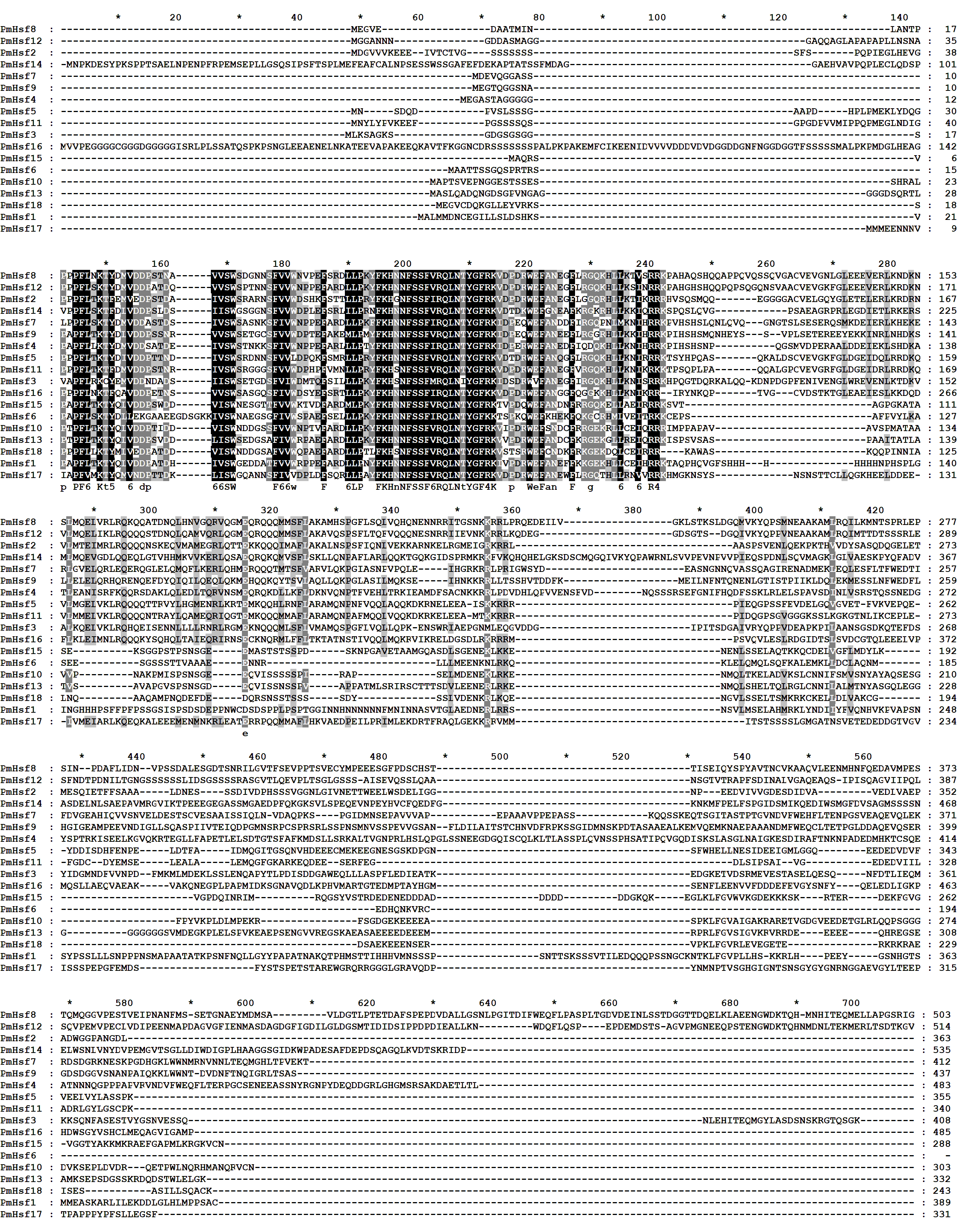

Supplement: Supplemental Information 1 [file peerj-07-7312-s001.png]

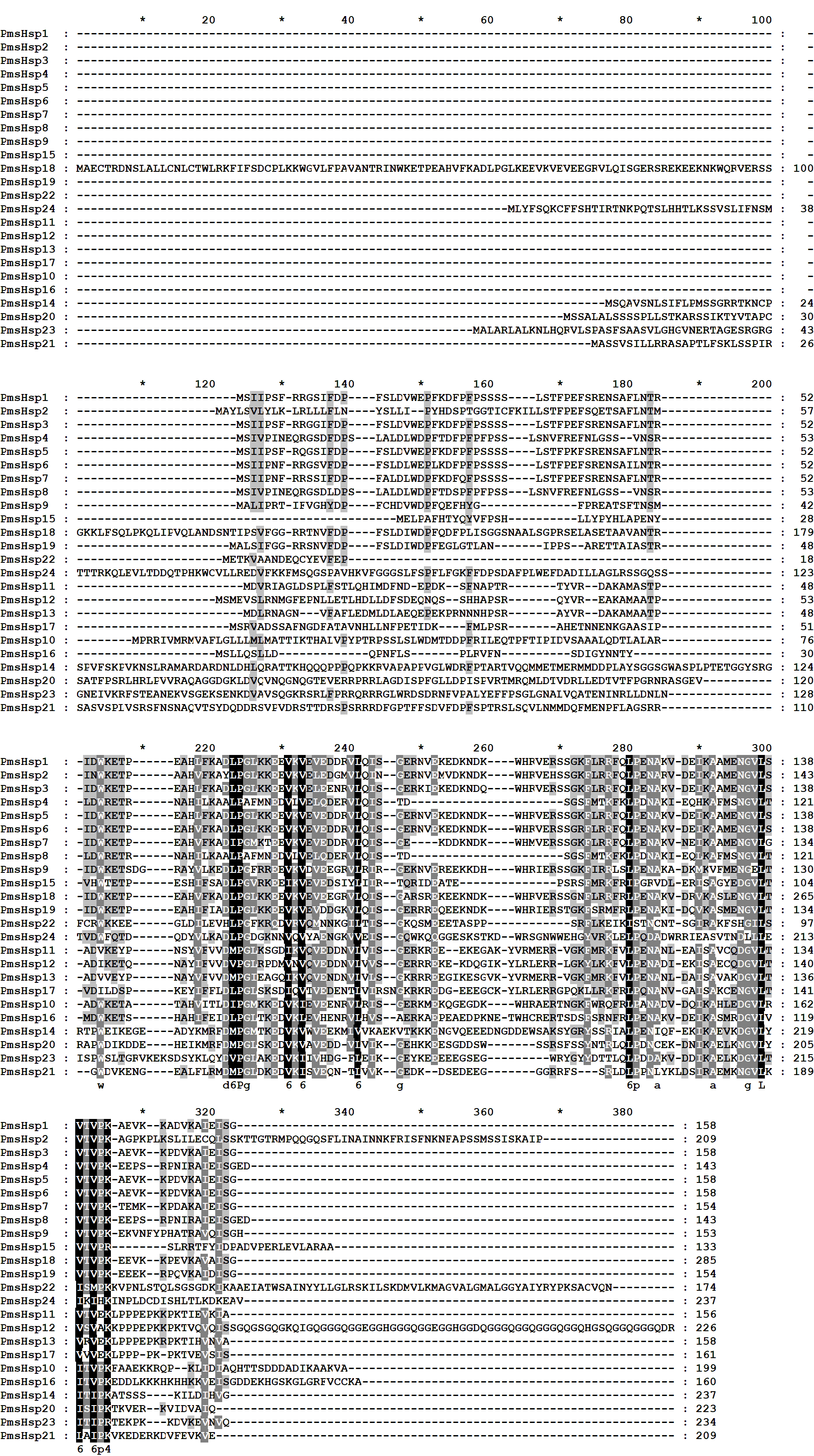

Supplement: Supplemental Information 2 [file peerj-07-7312-s002.png]

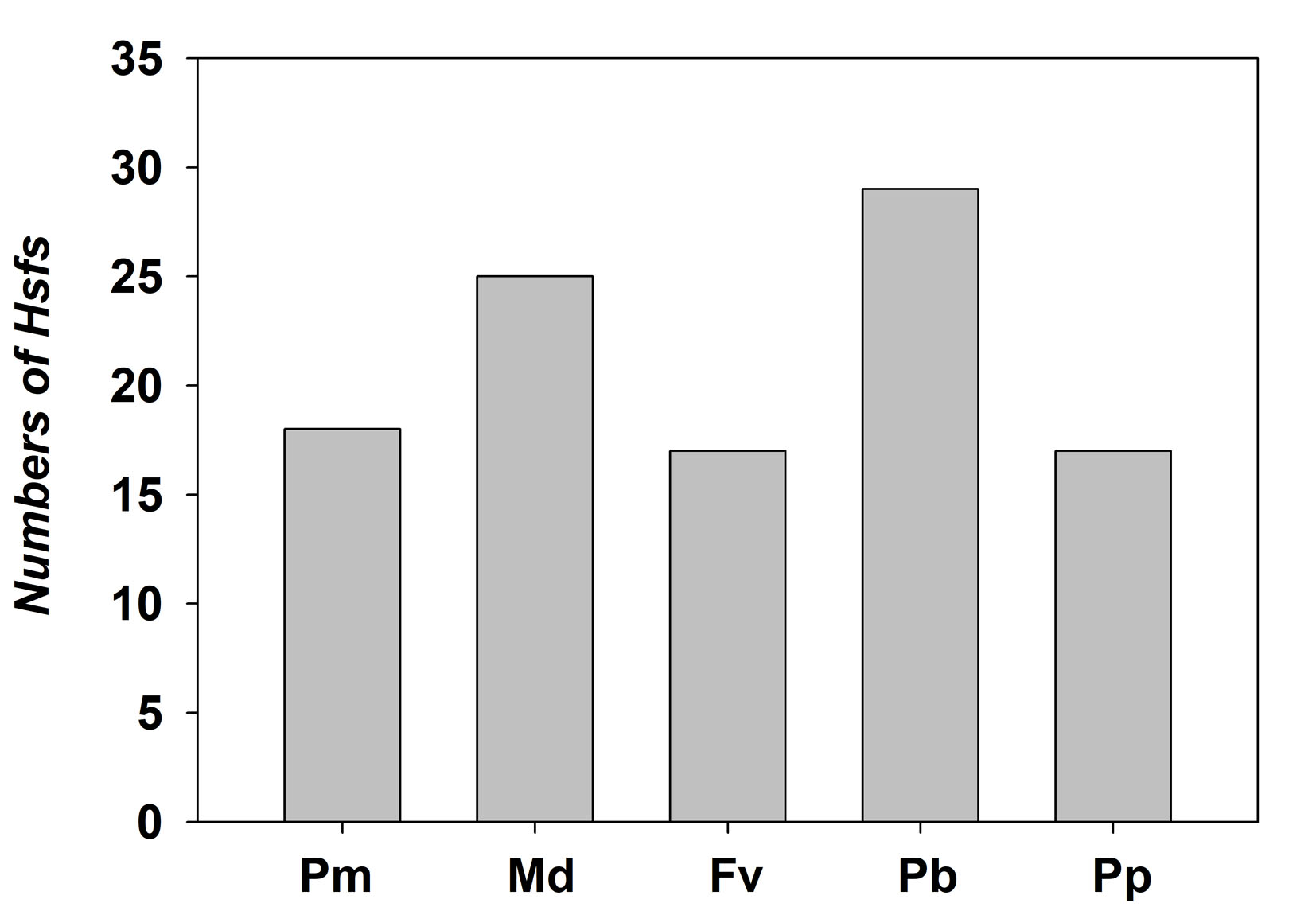

Supplement: Supplemental Information 3 [file peerj-07-7312-s003.png]
