# Supplementary material for: Genome-wide identification and classification of the Hsf and sHsp gene families in Prunus mume, and transcriptional analysis under heat stress
Source: PeerJ. 2019 Jul 29;7:e7312. doi: 10.7717/peerj.7312 (PMC6673427; doi:10.7717/peerj.7312)
Supplement: Supplemental Information 6 [file peerj-07-7312-s006.docx]

>PmHsf8

MEGVEDAATMINLANTPPPPFLNKTYDMVDDPSTNAVVSWSDGNNSFVVWNVPEFSRDLLPKYFKHNNFSSFVRQLNTYGFRKVDPDRWEFANEGFLRGQKHLLKTVSRRKPAHAQSHQQAPPQVQSSQVGACVEVGNLGLEEEVERLKNDKNSLMQELVRLRQKQQATDNQLHNVGQRVQGMEQRQQQMMSFLAKAMHSPGFLSQLVQHQNENNRRITGSNKKRRLPRQEDEILVGKLSTKSLDGQMVKYQPSMNEAAKAMLRQILKMNTSPRLEPSINPDAFLIDNVPSSDALESGDTSNRILGVTFSEVPPTSVECYMPEEESGFPDSCHSTTISEIQYSPYAVTNCVKAAQVLEENMHNFQEDAVMPESTQMQGGVPESTVEIPNANFMSSETGNAEYMDMSAVLDGTLPTETDAFSPEPDVDALLGSNLPGITDIFWEQFLPASPLTGDVDEINLSSTDGGTTDQELKLAEENGWDKTQHMNHITEQMELLAPGSRIG

>PmHsf12

MGGANNNGDDASMAGGGAQQAGLAPAPAPLLNSNAPPPFLSKTYDMVDDPATDQVVSWSPTNNSFVVWNPPEFARDLLPKYFKHNNFSSFVRQLNTYGFRKVDPDRWEFANEGFLRGQKHLLKSINRRKPAHGHSHQQPQPSQGQNSVAACVEVGKFGLEEEVERLKRDKNVLMQELIKLRQQQQSTDNQLQAMVQRLQGMEQRQQQMMSFLAKAVQSPSFLTQFVQQQNESNRRIIEVNKKRRLKQDEGGDSGTSDGQIVKYQPPVNEAAKAMLRQIMTTDTSSSRLESFNDTPDNILTGNGSSSSSSLIDSGSSSSRASGVTLQEVPLTSGLGSSSAISEVQSSLQAANSGTVTRAPFSDINALVGAQEAQSIPISQAGVIIPQLSQVPEMVPECLVDIPEENMAPDAGVGFIENMASDAGDGFIGDILGLDGSMTIDIDSIPPDPDIEALLKNWDQFLQSPEPDEMDSTSAGVPMGNEEQPSTENGWDKTQHNMDNLTEKMERLTSDTKGV

>PmHsf2

MDGVVVKEEEIVTCTVGSSSSSSSSFSPQPIEGLHEVGPPPFLTKTFEMVEDPSTDAIVSWSRARNSFVVWDSHKFSTTLLPRYFKHGNFSSFIRQLNTYGFRKVDPDRWEFANEGFLGGQRHLLKTIKRRRHVSQSMQQEGGGGACVELGQYGLETELERLKRDRNVLMTEIMRLRQQQQNSKEQVMAMEGRLQTTEKKQQQIMAFLAKALNSPSFIQNLVEKKARNKELRGMEIGRKRRLAASPSVENLQEKPKTHVVDYSASQDQGELETMESQIETFFSAAALDNESSSDIVDPHSSSVGGNLGIVNETTWEELWSDELIGGNPEEDVIVVGDESDIDVAVEDLVAEPADWGGPANGDL

>PmHsf14

MNPKDESYPKSPPTSAELNPENPFRPEMSEPLLGSQSIPSFTSPLMEFEAFCALNPSESSWSSGAFEFDEKAPTATSSFMDAGGAEHVAVPQPLECLQDSPVPPFLSKTFDLVDDPSLDSIISWGSGGNSFVVWDPLEFSRLILPRNFKHNNFSSFVRQLNTYGFRKVDTDKWEFGNEAFKRGKRHLLKKIQRRKSPQSLQVGPSAEAGRPRLEGDIETLRKERSMLMQEVGDLQQEQLGTVHHMKVVKERLQSAEQRQKQMVSFLSKLLQNPAFLARLQQKTGQKGIDSPRMKRKFVKQHQHELGKSDSCMQGQIVKYQPAWRNLSVVPEVNPVVPIEQSPDNLSQVMAGKLGLVAESKPYQFADVASDELNLSAEPAVMRGVIKTPEEEGEGASSMGAEDPFQKGKSVLSPEQEVNPEYHVCFQEDFGKNKMFPELFSPGIDSMIKQEDIWSMGFDVSAGMSSSSNELWSNLVNYDVPEMGVTSGLLDIWDIGPLHAAGGSGIDKWPADESAFDEPDSQAGQLKVDTSKRIDP

>PmHsf7

MDEVQGGASSLPPFLSKTYDMVDDASTDSIVSWSASNKSFIVWNPPEFARDLLPKFFKHNNFSSFIRQLNTYGFRKIDPEQWEFANDDFIRGQPNLMKNIHRRKPVHSHSLQNLQVQGNGTSLSESERQSMKDEIERLKHEKERLGVELQRLEQERQGLELQMQFLKERLQHMERQQQTMTSFVARVLQKPGIASNPVPQLEIHGRKRRLPRIGWSYDEASNGNNQVASSQAGIRENADMEKLEQLESFLTFWEDTIFDVGEAHIQVVSNVELDESTSCVESAAISSIQLNVDAQPKSPGIDMNSEPAVVVAPEPAAAVPPEPASSKQQSSKEQTSGITASTPTGVNDVFWEHFLTENPGSVEAQEVQLEKRDSDGRKNESKPGDHGKLWWNMRNVNNLTEQMGHLTPVEKT

>PmHsf9

MEGTQGGSNAPAPFLTKTYDLVDDPSSNRVVSWSETGCSFVVWDPTEFAKEMLPMYFKHNNFSSFVRQLNTYGFRKIDPEQWEFANEEFLRGGRHLLKKIHRRKPIHSHSMQNHEYSSVPLSETEREEYEKKINRLNHDKSLLELELQRHQRENQEFDYQIQILQEQLQKMEHQQKQYTSVLAQLLQKPGLASILMQKSEIHNKKRRLLTSSHVTDDFKMEILNFNTQNENLGTISTPIIKLDQLEKMESSLNFWEDFLHGIGEAMPEEVNDIGLLSQASPIIVTEIQDPGMNSRPCSPRSRLSSPNSMNVSSPEVVGSANFLDILAITSTCHNVDFRPKSSGIDMNSKPDTASAAEALKEMVQEMKNAEPAAANDMFWEQCLTETPGLDDAQEVQSERGDSDGGVSNANPAIQKKLWWNTDVDNFTNQIGRLTSAS

>PmHsf4

MEGASTAGGGGGPAPFLLKTYDMVDDSATDEIVSWSTNKKSFIVWNPPEFARLLLPTYFKHNNFSSFIRQLNTYGFRKIDPERWEFANEDFIQDQKHLLKNIHRRKPIHSHSNPQGSMVDPERAALDDEIEKLSHDKATLEANISRFKQQRSDAKLQLEDLTQRVNSMEQRQKDLLKFLDKNVQNPTFVEHLTRKIEAMDFSACNKKRRLPDVDHLQPVVENSFVDNQSSSRSEFGNIFHQDFSSKLRLELSPAVSDINLVSRSTQSSNEDGYSPTRKISEELKGVQKRTEGLLFAPETLELSDTGTSFAFKMDSLLSRKALTVGNPRLHSLQPGLSSNEEGDGQISCQLKLTLASSPLQVNSSPHSATIPQVGQDISKSLASGLNAIGKESDIRAFTNKNPADEDMHKTCSQEATNNNQGPPPAPVRVNDVFWEQFLTERPGCSENEEASSNYRGNPYDEQDDGRLGHGMSRSAKDAETLTL

>PmHsf5

MNSDQDPVSLSSSGAAPDHPLPMEKLYDQGPPPFLTKTYDIVDDPTTNDIVSWSRDNNSFVVLDPQKFSMRLLPRYFKHNNFSSFVRQLNTYGFRKVDTDRWEFANQGFLRGQKHLLKNIRRRKTSYHPQASQKALDSCVEVGKFGLDGEIDQLRRDKQVLMGELVKLRQQQQTTRVYLHGMENRLKRTEMKQQHLRNFLARAMQNPNFVQQLAQQKDKRNELEEAISKKRRRPIEQGPSSFEVDELGQVGVETFVKVEPQEYDDISDHFENPELDTFAIDMQGITGSQNVHDEEECMEKEEGNESGSKDPGNSFWHELLNESIDEEIGMLGGQEEDEDVDVFVEELVYLASSPK

>PmHsf11

MNYLYPVKEEFPGSSSSQSGPGDPVVMIPPQPMEGLNDIGPPPFLTKTFDMVDDPSTNRIVSWSRGGGSFVVWDPHPFVMNLLPRYFKHSNFSSFVRQLNTYGFRKVDPDRWEFANEGFVRGQKHLLKNIKRKKTPSQPLPAQQALGPCVEVGRFGLDGEIDRLRRDKQVLMMELVKLRQQQQNTRAYLQAMEQRIQGTEMKQQQMMAFLARAMQNPAFMQQLVQQKDKRKELEEAMTKKRRRPIDQGPSGVGGGKSSLKGKGTNLIKCEPLEFGDCDYEMSELEALALEMQGFGKARKEQDEESERFEGDLSIPSAIVGEDEDVIILADRLGYLGSCPK

>PmHsf3

MLKSAGKSGDGSGSGGSVAPFLRKCYEMVDDNDADSIISWSETGDSFVIWDMTQFSILLLPKYFKHSNFSSFMRQLNIYGFRKIDSDRWVFANEGFIRGQKHLLKNISRRKHPQGTDQRKALQQKDNPDGPFENIVENGLWREVENLKTDKVALKQELVKLRQHQEISENNLLLLRNRLRGMEKNQQQMLSFLVMAMQSPGFLVQLLQPKENSWRIAEPGNMLEQGVDDGIPITSDGAIVRYQPPVDEAPKPILAANSGSDKQTEFDSYIDGMNDFVVNPDFMKMLMDEKLSSLENQAPYTLPDISDDGAWEQLLLASPFLEDIEATKEDGKETVDSRMEVESTASELQESQNFDTLIEQMKKSQNFASESTVYGSNVESSQNLEHITEQMGYLASDSNSKRGTQSGK

>PmHsf16

MVVPEGGGGCGGGDGGGGGISRLPLSSATQSPKPSNGLEEAENELNKATEEVAPAKEEQKAVTFKGGNCDRSSSSSSSPALPKPAKEMFCIKEENIDVVVVDDDVDVDGGDDGNFNGGDGGTFSSSSSMALPKPMDGLHEAGPPPFLNKTFQAVDDPETNSVVSWSASGQSFIVWDSYEFSRTLLPKYFKHNNFSSFIRQLNTYGFKKVDPDRWEFANGGFQGGKKHLLKNIKRRIRYNKQPTVGCVDSTKTGLEAEIESLKKDQDFLKLEIMNLRQQQKYSQHQLTAIEQRIRNSECKNQRMLFFLTKTATNSTIVQQLMQKRVIKRELDGSDLRKRRRMPSVQVLESLRDGIDTSLSVDCGTQLEEELVPMQSLLAEQVAEAKVAKQNEGPLPAPMIDKSGNAVQDLKPHVMARTGTEDMPTAYHGMSENFLEENVVFDDDEFEVGYSNFYQELEDLIGKPHDWSGYVSHCLMEQAGVIGAMP

>PmHsf15

MAQRSVPAPFLTKTYQLVDDPSWDDVISWNESGTTFVVWKTVDFARDMLPKYFKHNNFSSFVRQLNTYGFRKTVPDQWEFANDNFRRGQKELLAEIRRRKSVTAGPGKATASEKSGGPSTPSNSGEEMASTSTSSPDSKNPGAVETAAMGQASDLSGENEKLKKENENLSSELAQTKKQCDELVGFLMDYLKVGPDQINRIMRQGSYVSTRDEDENEDDDADDDDDDDGKQKEGLKLFGVWVKGDEKKKSKRTERDEKFGVGVGGTYAKKMKRAEFGAPMLKRGKVCN

>PmHsf6

MAATTSSGQSPRTRSPAPFLSKTYDLLEKGAAEEGDSGKKIVSWNAEGSGFIVWSPAEFSELLLPKYFKHNNFSSFIRQLNTYGFKKTSPKQWEFKHEKFQKGCRHMLVEITRKKCEPSAFPVYLKASEESGSSSTTVAAAEENNRLLLMEENKNLRKQKLELQMQLSQFKALEMKLLDCLAQNMEDHQNKVRC

>PmHsf10

MAPTSVEPNGGESTSSESSHRALPTPFLTKTYQLVDDPTIDDVISWNDDGSSFVVWNPTVFARDLLPKYFKHNNFSSFVRQLNTYGFRKVIPDRWEFSNDCFRRGEKRLLCEIQRRRIMPPAPAVAVSPMATAAVVPNAKPMISPSNSGEEQVISSSSSPIRAPSELMDENEKLRKENMQLTKELADVKSLCNNIFSMVSNYAYAQSESGFPYVKPLDLMPEKRFSGDGEKEEEEASPKLFGVAIGAKRARETVGDGVEEDETGLRLQQPSGGGDVKSEPLDVDRQETPWLNQRHMANQRVCN

>PmHsf13

MASLQADQNGDSGPVNGAGGGGDSQRTLPTPFLTKTYQLVDDPSVDDLISWSEDGSAFIVWRPAEFARDLLPKYFKHNNFSSFVRQLNTYGFRKVVPDRWEFANDCFKRGEKGLLREIQRRKISPSVSASPAAITATLATVSAVAPGVSPSNSGDEQVISSNSSPVAPPATMLSRIRSCTTTSDVLEENERLRKENMQLSHELTQLRGLCNNILALMTNYASGQLEGGGGGGGGGSVMDEGKPLELSPVKEAEPSENGVVREGSKAEASAEEEEDEEEMRPRLFGVSIGVKRVRRDEEEEEQHREGSEAMKSEPSDGSSKRDQDSTWLELGK

>PmHsf18

MEGVCDQKGLLEYVRKSSPPPFLLKTYMLVEDPATDDVISWNDDGSAFVVWQPAEFARDLLPTLFKHSNFSSFVRQLNTYGFRKVSTSRWEFCNDKFRKGEKDQLCEIRRRKAWASKQQPINNIALNQAAQAMPNQDEFDEDQRSNSSTSSSSDYSSLVDENKRLKQENGVLSSELTSMKRKCKELLDLVAKCGDSAEKEEENSERVPKLFGVRLEVEGETERKRKRAEISESASILLSQACK

>PmHsf1

MALMMDNCEGILLSLDSHKSVPAPFLTKTYQLVDDPATDHIVSWGEDDATFVVWRPPEFARDLLPNYFKHNNFSSFVRQLNTYGFRKIVPDRWEFANEFFKKGEKHLLCEIHRRKTAQPHQVGFSHHHHHHHHNPHSPLGINGHHHPSFFPFPSSGSISPSDSDEPPNWCDSDSPPLPSPTGGINNHNNNNNNFMNINNASVTGLAEDNERLRRSNSVLMSELAHMRKLYNDIIYFVQNHVKPVAPSNSYPSSLLLSNPPPNSMAPAATATKPSNFNQLLGYYPAPATNAKQTPHMSTTIHHHVMNSSSPSNTTSKSSSVTILEDQQQPSSNGCKNTKLFGVPLLHSKKRLHPEEYGSNHGTSMMEASKARLILEKDDLGLHLMPPSAC

>PmHsf17

MMMEENNNVIAPFVMKTYQMVNDPTTDKLISWGQANNSFIVVDPLDFSQRLLPAYFKHNNFSSFVRQLNTYGFRKVDPDRWEFANEWFLRGQTHLLRNVVRRKHMGKNSYSNSNSTTCLLQGKHEELDDEEIVMEIARLKQEQKALEEEMENMNKRLEATERRPQQMMAFLHKVAEDPEILPRIMLEKDRTFRAQLGEKKRRVMMITSTSSSSLGMGATNSVETEDEDDGTVGVISSSPEPGFEMDSFYSTSPETSTAREWGRQRRGGGLGRAVQDPYNMNPTVSGHGIGNTSNSGYGYGNRNGGAEVGYLTEEPTPAPPPYPFSLLEGSF

>PmsHsp1

MSIIPSFRRGSIFDPFSLDVWEPFKDFPFPSSSSLSTFPEFSRENSAFLNTRIDWKETPEAHLFKADLPGLKKEEVKVEVEDDRVLQISGERNVEKEDKNDKWHRVERSSGKFLRRFQLPENAKVDEIKAAMENGVLSVTVPKAEVKKADVKAIEISG

>PmsHsp2

MAYLSVLYLKLRLLLFLNYSLLIPYHDSPTGGTICFKILLSTFPEFSQETSAFLNTMINWKETPAAHVFKAYLPGLKKEEVKVELEDGMVLQINGERNVEMVDKNDKWHRVEHSSGKFLRRFQLPENARVDEIKAAMENGVLSVTVPKAGPKPLKSLILECQLSSKTTGTRMPQQGQSFLINAINNKFRISFNKNFAPSSMSSISKAIP

>PmsHsp3

MSIIPSFRRGGIFDPFSLDVWEPFKDFPFPSSSSLSTFPEFSRENSAFLNTRIDWKETPEAHLFKADLPGLKKEEVKVELEENRVLQISGERKIEKEDKNDQWHRVERSSGKFLRRFQLPENAKVDEIKAAMENGVLSVTVPKAEVKKPDVKAIEISG

>PmsHsp4

MSIVPINEQRGSDFDPSLALDLWDPFTDFPFPFPSSLSNVFREFNLGSSVNSRLDWRETRNAHILKAALPAFMNEDVLVELQDERVLQISTDSGSFMTKFKLPDNAKIEQHKAFMSNGVLTVTVPKEEPSRPNIRAIEISGED

>PmsHsp5

MSIIPSFRQGSIFDPFSLDVWEPFKDFPFPSSSSLSTFPKFSRENSAFLNTRIDWKETPEAHLFKADLPGLKKEEVKVEVEDDRVLQISGERNVEKEDKNDKWHRVERSSGKFLRRFQLPENAKVDEIKAAMENGVLSVTVPKAEVKKPDVKAIEISG

>PmsHsp6

MSIIPNFRRGSVFDPFSLDLWEPLKDFPFPSSSSLSTFPEFSRENSAILNTRIDWKETPEAHVFKADLPGLKKEEVKVEVEDDRVLQISGERNVEKEDKNDKWHRVERSSGRFLRRFQLPENAKVDEIKAAMENGVLSVTVPKAEVKKPDVKAIEISG

>PmsHsp7

MSIIPNFRRSSIFDPFALDLWDPFKDFQFPSSSSLSTFPEFSRENSAFLNTRIDWKETPEAHVFKADIPGMKTEEVKVEVEDDRVLQISGEKDDKNDKWHMVERSSGKFLRRFQLPENAKVNEIKAAMENGVLGVTVPKTEMKKPDAKAIEISG

>PmsHsp8

MSIVPINEQRGSDLDPSLALDLWDPFTDSPFPFPSSLSNVFREFNLGSSVNSRLDWRETRNAHILKAALPAFMNEDVLVELQDERVLQISTDSGSFMTKFKLPDNAKIEQLKAFMSNGVLTVTVPKEEPSRPNIRAIEISGED

>PmsHsp9

MALIPRTIFVGHYDPFCHDVWDPFQEFHYGFPREATSFTNSMIDWKETSDGRAYVLKEDLPGFRREEVKVDVEEGRVLRIRGEKNVEREEKKDHWHRIERSSGKFIRRLSLPENAKADKMKVFMENGELTVTVPKEKVNFYPHATRAVQISGH

>PmsHsp15

MELPAFHTYQYVFPSHLLYPYHLAPENYVHWTETPESHIFSADLPGVRKEEIKVEVEDSIYLIIRTQRIDEATEPSRSFMRKFRIPGRVDLERISAGYEDGVLTVTVPRSLRRTFYIDPADVPERLEVLARAA

>PmsHsp18

MAECTRDNSLALLCNLCTWLRKFIFSDCPLKKWGVLFPAVANTRINWKETPEAHVFKADLPGLKEEVKVEVEEGRVLQISGERSREKEEKNKWQRVERSSGKKLFSQLPKQLIPVQLANDSNT*****IPSVFGGRRTNVFDPFSLDIWDPFQDFPLISGGSNAALSGPRSELASETAAVANTRIDWKETPEAHVFKADLPGLKKEEVKVEVEEGRVLQISGARSREKEEKNDKWHRVERSSGNFLRRFRLPENAKVDRVKASLENGVLTVTVPKEEVKKPEVKAVAISG

>PmsHsp19

MALSIFGGRRSNVFDPFSLDIWDPFEGLGTLANIPPSARETTAIASTRIDWKETPEAHIFIADLPGLKKEEVKVEVDDGKVLQISGERRREQEEKNDKWHRIERSTGKFSRMFRLPENAKIDQVKASMENGVLTVTVPKEEEKRPQVKAIDISG

>PmsHsp22

METKVAANDEQCYEVFEPFCRWKKEEGLDILEVHLPGFKRQDVRVQMNNKGILTISGKQSMEEETASPPSRFLKEIKISTNCNTSGIRAKFSHGILSISMPKKVPNLSTQLSGSGDKIKAAEIATWSAINYYLLGLRSKILSKDMVLKMAGVALGMALGGYAIYRYPKSACVQN

>PmsHsp24

MLYFSQKCFFSHTIRTNKPQTSLHHTLKSSVSLIFNSMTTTRKQLEVLTDDQTPHKWCVLLREDVFKKFMSQGSPAVHKVFGGGSLFSPFLFGKFFDPSDAFPLWEFDADILLAGLRSSGQSSTVDWFQTDQDYVLKADLPGDGKNNVQVYAENGKVVEISGQWKQQGGESKSTKDWRSGNWWEHGYVRKLELPQDADWRRIEASVTNDLLLEIKIHKINPLDCDISHLTLKDKEAV

>PmsHsp11

MDVRIAGLDSPLFSTLQHIMDFNDEPDKSFNAPTRTYVRDAKAMASTPADVKEYPNSYVFVVDMPGLKSGDIKVQVEDDNVLVISGERKREEEKEGAKYVRMERRVGKFMRKFVLPENANLEAISAVCQDGVLTVTVEKLPPPEPKKPKTIEVKIA

>PmsHsp12

MSMEVSLRNMGFEPNLLETLHDLLDFSDEQNQSSHHAPSRQYVREAKAMAATPADIKETQNAYIFVVDVPGLRPDMVNVQVEDDNVLVVSGERRREKEKDQGIKYLRLERRLGKYLKKFVLPENADIEKISAECQDGVLTVSVAKKPPPEPKKPKTVQVQISSGQGSGQGKQIGQGGGQGGEGGHGGGQGGEGGHGGDQGGGQGGQGGGQGGQHGSQGGQGGGQDR

>PmsHsp13

MDLRNAGNVFAFLEDMLDLAEQEPEKPRNNNHPSRAYVRDAKAMAATPADVVEYPNAYVFVVDMPGIEAGQIKVQVENDNVLVLSGKRRREEGIKESGVKYVRMERRVGKFMRKFVLPENANLDAISAVAKDGVLTVRVEKLPPPEPKRPKTIHVNVA

>PmsHsp17

MSRVADSSAFNGDFATAVNHLLNFPETIDKFMLPSRAHETNNENKGAASIPVDILDSPKEYIFFLDLPGLSKSDIQVTVEDENTLVIRSNGKRKREDGEEEGCKYLRLERRGPQKLLRKFRLPQNANVGAISAKCENGVLTVVVEKLPPPPKPKTVEVSIS

>PmsHsp10

MPRRIVMRMVAFLGLLLMLMATTIKTHALVPYPTRPSSLSLWDMTDDPFRILEQTPFTIPIDVSAAALQDTLALARADWKETATAHVITLDIPGMKKEDVKIEVEENRVLRISGERKMEKQGEGDKWHRAERTNGKFWRQFRLPANADVDQIKAHLEDGVLRITVPKFAAEKKRQPKLIDIAQHTTSDDDADIKAAKVA

>PmsHsp16

MSLLQSLLDQPNFLSPLRVFNSDIGYNNTYMDWKETSHAHIFEIDLPGLTKEDVKLEVHENRVLHVSAERKAEPEAEDPKNETWHCRERTSDSFSRNFRLPENAKVDEIKASMRDGVLVITVPKEDDLKKKHKHHKKVEISGDDEKHGSKGLGRFVCCKA

>PmsHsp14

MSQAVSNLSIFLPMSSGRRTKNCPSPVFSKPVKNSLRAMARDARDNLDHLQRATTKHQQQPPPQPKKRVAPAPPVGLWDRFPTARTVQQMMETMERMMDDPLAYSGGSGWASPLPTETGGYSRGRTPWEIKEGEADYKMRFDMPGMTKEDVKVWVEEKMLVVKAEKVTKKKENGVQEEEDNGDDEWSAKSYGRYSSRIALPENIQFEKIKAEVKDGVLYITIPKATSSSKILDIHVG

>PmsHsp20

MSSALALSSSSPLLSTKARSSIKTYVTAPCSATFPSRLHRLPVVRAQAGGDGKLDVQVNQGNQGTEVERRPRRLAGDISPFGLLDPISPVRTMRQMLDTVDRLLEDTVTFPGRNRASGEVRAPWDIKDDEHEIKMRFDMPGLSKEDVKVAVEDDVLVIKGEHKKEESGDDSWSSRSFSSYNTRLQLPDNCEKDNIKAELKNGVLYISIPKTKVERKVIDVAIQ

>PmsHsp23

MALARLALKNLHQRVLSPASFSAASVLGHGVNERTAGESRGRGGNEIVKRFSTEANEKVSGEKSENKDVAVSQGKRSRLFPRRQRRRGLWRDSDRNFVPALYEFFPSGLGNALVQATENINRLLDNLNISPWSLTGRVKEKSDSYKLQYDVPGLAKEDVKIIVHDGFLEIKGEYKEEEEEGSEGWRYGYYDTTLQLPDDAKVDDIKAELKDGVLTITIPRTEKPKKDVKEVNVQ

>PmsHsp21

MASSVSILLRRASAPTLFSKLSSPIRSASVSPLVSRSFNSNAQVTSYDQDDRSVPVDRSTTDRSPSRRRDFGPTFFSDVFDPFSPTRSLSQVLNMMDQFMENPFLAGSRRGWDVKENGEALFLRMDMPGLDKEDVKISVEQNTLVVKGEDKDSEDEEGGGRRFSSRLDLPPNLYKLDSIRAEMKNGVLKLAIPKVKEDERKDVFEVKVE
