# Supplementary material for: Genome-wide identification and classification of the Hsf and sHsp gene families in Prunus mume, and transcriptional analysis under heat stress
Source: PeerJ. 2019 Jul 29;7:e7312. doi: 10.7717/peerj.7312 (PMC6673427; doi:10.7717/peerj.7312)
Supplement: Supplemental Information 7 [file peerj-07-7312-s007.docx]

>PmHsf8

ATGGAAGGAGTCGAAGACGCGGCGACAATGATCAACTTGGCCAACACTCCTCCTCCGCCCTTTCTGAACAAAACCTACGACATGGTGGACGACCCGTCGACCAACGCCGTCGTTTCCTGGAGCGACGGCAACAACAGCTTCGTCGTTTGGAACGTGCCCGAGTTCTCCAGGGACCTCCTGCCCAAGTATTTCAAGCACAACAACTTCTCTAGCTTCGTCAGGCAGCTGAATACTTATGGTTTTAGAAAGGTTGATCCAGACCGATGGGAATTTGCAAATGAAGGGTTTCTAAGAGGCCAGAAACACCTTTTGAAGACTGTCAGCAGGCGGAAACCAGCTCATGCACAGAGTCATCAACAAGCACCACCTCAAGTGCAGAGCTCTCAGGTTGGGGCATGTGTTGAGGTGGGAAATCTTGGGCTGGAGGAAGAGGTCGAAAGACTTAAAAATGACAAGAACAGTCTTATGCAGGAACTTGTTAGATTGAGGCAGAAACAGCAAGCAACAGATAATCAGTTACATAATGTTGGACAACGTGTGCAGGGAATGGAGCAGCGGCAGCAGCAAATGATGTCATTTCTTGCAAAGGCTATGCATAGTCCTGGATTTTTATCCCAGCTTGTACAGCATCAAAATGAAAACAACAGGCGAATCACTGGAAGCAATAAGAAAAGGAGACTCCCAAGGCAAGAAGATGAAATTTTGGTTGGGAAGCTTAGCACCAAATCTCTCGATGGACAAATGGTGAAGTACCAACCTTCAATGAATGAAGCAGCAAAAGCAATGCTGCGGCAGATCTTGAAGATGAATACATCTCCTAGGCTGGAACCGTCAATCAATCCTGATGCTTTCCTGATTGACAATGTTCCTTCTTCTGATGCATTAGAAAGCGGTGACACCTCTAACCGGATTTTGGGCGTGACCTTTTCAGAGGTTCCACCAACTTCTGTGGAGTGTTATATGCCTGAAGAAGAGTCTGGATTTCCCGACAGTTGTCATTCTACAACCATTTCTGAGATCCAGTATTCTCCTTATGCGGTTACCAATTGTGTTAAAGCAGCTCAAGTTTTGGAAGAGAACATGCATAATTTTCAAGAAGATGCAGTTATGCCTGAGTCAACGCAAATGCAAGGTGGTGTTCCAGAAAGCACTGTAGAAATCCCCAACGCAAACTTCATGAGCTCTGAGACTGGGAATGCAGAGTACATGGATATGTCTGCGGTTTTGGATGGGACCCTGCCTACAGAAACTGATGCCTTTTCTCCTGAGCCTGATGTAGATGCTTTGCTGGGCTCTAACCTTCCGGGAATTACTGATATCTTCTGGGAACAATTTCTTCCAGCGAGCCCTCTGACTGGTGACGTAGATGAAATTAATTTGAGTTCCACTGATGGGGGGACCACGGATCAAGAGTTGAAGTTGGCGGAGGAGAATGGATGGGACAAAACTCAACATATGAATCATATTACTGAACAAATGGAGCTTCTTGCACCAGGGAGCAGAATTGGTTGA

>PmHsf12

ATGGGAGGTGCTAATAACAACGGCGACGATGCATCAATGGCCGGCGGCGGAGCCCAGCAGGCGGGTTTGGCTCCGGCGCCAGCACCGTTACTGAACTCGAACGCGCCGCCGCCTTTTCTGAGCAAGACGTACGACATGGTGGACGACCCGGCGACCGACCAGGTTGTGTCGTGGAGCCCCACCAACAACAGCTTCGTGGTTTGGAACCCGCCGGAGTTTGCTAGGGACCTTCTGCCCAAGTACTTCAAGCACAATAACTTCTCCAGCTTTGTGAGGCAGTTGAACACCTATGGATTTAGGAAGGTTGACCCAGACCGCTGGGAATTTGCAAATGAGGGCTTTTTGAGGGGTCAGAAGCATCTCCTTAAGAGTATCAACCGGCGAAAACCTGCGCATGGACATAGTCATCAACAGCCACAGCCATCACAAGGACAGAATTCAGTGGCTGCATGTGTAGAGGTTGGGAAGTTTGGCCTTGAGGAAGAAGTTGAGAGGCTAAAAAGGGACAAGAATGTGCTTATGCAGGAACTTATCAAGTTGAGGCAGCAGCAACAGTCTACTGATAACCAGCTGCAAGCAATGGTGCAACGTCTTCAGGGTATGGAACAGCGGCAGCAACAGATGATGTCATTCCTTGCAAAGGCTGTGCAGAGCCCAAGTTTCTTGACTCAGTTTGTACAGCAGCAGAATGAGAGCAATAGGCGCATAATTGAAGTCAACAAAAAACGGAGGCTCAAGCAGGATGAGGGCGGGGACTCTGGTACTTCTGATGGACAGATTGTGAAGTATCAGCCTCCAGTGAATGAAGCTGCAAAGGCAATGCTCAGGCAGATTATGACAACAGATACTTCTTCTTCCCGGCTGGAATCTTTTAATGACACCCCTGATAACATCCTGACTGGGAATGGCTCATCATCATCATCCAGTTTAATAGACAGTGGGAGCTCTTCAAGCCGTGCGTCAGGAGTGACCCTTCAAGAAGTCCCGCTGACTTCAGGGCTTGGTTCGTCATCTGCAATTTCTGAAGTACAATCTTCTCTACAGGCTGCAAACTCTGGAACAGTTACGAGAGCTCCATTCTCAGATATAAATGCCCTCGTTGGAGCACAAGAGGCACAATCAATCCCTATTTCTCAGGCTGGTGTAATCATCCCCCAGCTTTCTCAAGTACCGGAAATGGTACCCGAATGTTTAGTTGATATTCCTGAAGAAAACATGGCACCTGATGCGGGTGTTGGATTTATTGAAAACATGGCATCTGACGCAGGTGATGGATTTATCGGCGACATATTGGGATTGGATGGGTCAATGACCATAGACATTGATAGTATTCCTCCTGATCCTGACATAGAAGCATTGCTAAAGAATTGGGATCAATTTTTACAAAGTCCAGAGCCAGATGAAATGGATTCTACTTCTGCAGGAGTGCCCATGGGAAATGAAGAGCAGCCGTCAACAGAGAATGGATGGGACAAAACCCAGCATAATATGGATAACCTTACTGAAAAAATGGAACGTCTTACTTCAGATACCAAAGGGGTTTGA

>PmHsf16

ATGGTGGTGCCTGAAGGTGGTGGTGGTTGTGGCGGTGGGGATGGTGGTGGTGGTGGTATATCTCGTCTGCCACTGTCATCAGCAACCCAGTCGCCAAAACCCAGCAATGGTTTAGAAGAAGCAGAGAACGAGTTGAATAAAGCCACAGAAGAAGTGGCTCCGGCCAAGGAAGAGCAAAAGGCAGTGACTTTCAAAGGTGGTAATTGTGATCGGTCATCTTCTTCGTCTTCATCCCCTGCTTTGCCAAAACCAGCCAAGGAAATGTTCTGTATTAAAGAAGAAAACATAGATGTTGTAGTTGTGGATGATGATGTTGATGTTGATGGGGGAGATGATGGGAACTTTAATGGTGGTGATGGTGGTACATTTTCTTCATCTTCATCCATGGCGTTGCCTAAACCCATGGACGGTTTGCACGAGGCAGGACCACCACCCTTCTTGAACAAGACTTTTCAAGCGGTGGATGACCCAGAAACCAATTCGGTTGTTTCATGGAGTGCATCTGGTCAGAGCTTCATTGTTTGGGACTCTTACGAGTTCTCCAGGACTCTCTTGCCCAAATACTTCAAGCACAACAATTTCTCAAGCTTCATCCGCCAACTCAACACTTATGGCTTCAAGAAGGTTGATCCGGACAGGTGGGAGTTTGCAAATGGAGGGTTTCAGGGAGGGAAGAAGCACTTGCTGAAGAACATCAAGAGAAGAATCAGATATAACAAGCAGCCAACTGTAGGCTGTGTTGATTCAACCAAAACTGGGTTAGAAGCCGAAATTGAAAGTCTAAAGAAAGACCAAGACTTTTTGAAATTGGAAATCATGAATCTCAGACAACAACAAAAGTACTCACAGCATCAACTGACTGCTATTGAACAACGAATTCGAAATTCAGAGTGTAAGAACCAAAGGATGCTCTTTTTCCTCACCAAAACAGCCACAAATTCCACCATTGTCCAGCAGCTAATGCAGAAGAGAGTGATAAAGAGAGAGCTGGATGGGAGTGATCTACGCAAGAGAAGGAGAATGCCTTCAGTCCAAGTCCTTGAAAGCTTGCGTGATGGGATTGATACCAGCCTCAGTGTGGATTGTGGAACCCAACTTGAGGAAGAACTGGTGCCTATGCAGTCTCTGCTTGCTGAACAAGTGGCAGAGGCAAAGGTTGCCAAACAAAATGAAGGCCCATTGCCAGCTCCCATGATTGATAAATCAGGCAATGCAGTTCAAGATCTTAAGCCCCATGTGATGGCCAGAACCGGCACGGAAGACATGCCCACTGCTTATCATGGCATGTCTGAGAATTTCCTGGAGGAAAATGTAGTCTTTGATGATGATGAATTTGAAGTCGGTTACTCTAATTTTTATCAAGAATTGGAGGATTTGATTGGCAAGCCACATGATTGGAGTGGTTATGTAAGTCACTGCCTGATGGAGCAAGCTGGGGTAATTGGGGCCATGCCTTGA

>PmHsf2

ATGGATGGAGTGGTGGTGAAAGAGGAAGAGATTGTGACATGCACTGTTGGTTCATCATCCTCTTCTTCTTCAAGCTTCTCACCTCAGCCAATAGAGGGCTTACACGAAGTGGGCCCTCCCCCTTTTCTCACAAAGACCTTTGAAATGGTGGAGGATCCTTCTACAGACGCCATTGTCTCCTGGAGCAGAGCTCGCAACAGTTTCGTTGTTTGGGACTCTCATAAGTTCTCCACCACTCTTCTTCCTCGCTACTTCAAGCACGGAAACTTCTCCAGCTTCATTCGTCAGCTTAATACATATGGTTTTAGAAAGGTTGATCCTGACCGATGGGAATTTGCAAACGAAGGGTTTCTGGGAGGGCAGAGGCATTTACTGAAGACCATCAAGAGGAGGAGGCATGTGTCACAGAGTATGCAACAAGAAGGTGGAGGAGGAGCTTGTGTTGAACTGGGCCAGTATGGACTGGAGACTGAGCTTGAAAGATTGAAGAGAGACCGAAATGTTTTAATGACTGAAATAATGAGGCTGAGGCAGCAACAACAAAATTCAAAGGAACAAGTCATGGCAATGGAGGGTCGGTTGCAGACCACAGAGAAAAAACAACAGCAGATTATGGCTTTCCTTGCCAAAGCACTCAATAGCCCATCTTTTATCCAAAACCTTGTTGAGAAGAAGGCTCGGAATAAAGAGTTGCGTGGTATGGAAATTGGTCGAAAGCGGAGACTAGCAGCTAGCCCCAGTGTGGAGAATCTGCAAGAAAAACCTAAAACCCATGTTGTGGACTACTCAGCAAGCCAAGATCAGGGAGAGTTGGAAACTATGGAATCGCAGATTGAGACCTTTTTCTCAGCTGCTGCATTGGATAATGAATCAAGCAGTGATATCGTAGACCCTCATTCAAGTTCAGTTGGTGGCAACTTAGGCATTGTTAATGAGACTACATGGGAGGAGCTGTGGAGCGATGAGCTCATTGGTGGCAATCCGGAGGAAGACGTTATTGTGGTGGGTGATGAATCAGATATTGATGTCGCGGTGGAGGATTTGGTTGCAGAGCCAGCAGATTGGGGCGGACCGGCCAACGGAGACCTTTGA

>PmHsf14

ATGAACCCAAAAGACGAGAGCTACCCAAAGTCTCCACCTACTTCAGCCGAATTGAACCCGGAGAATCCGTTTCGACCCGAAATGTCAGAACCGTTGTTGGGTTCTCAGTCAATTCCTTCGTTTACTTCTCCTCTGATGGAATTTGAAGCCTTTTGTGCTCTAAACCCATCGGAGTCTTCTTGGTCCTCTGGTGCTTTCGAGTTTGATGAGAAAGCTCCAACTGCAACGTCTTCGTTTATGGACGCTGGTGGCGCGGAGCACGTGGCCGTCCCGCAGCCTCTTGAATGTCTACAGGACAGTCCGGTTCCGCCGTTTCTGTCCAAGACTTTCGATCTGGTCGACGACCCGTCGCTTGATTCGATCATATCGTGGGGTTCCGGCGGCAACAGCTTCGTGGTGTGGGACCCATTGGAGTTTTCCAGGCTCATCTTGCCGAGGAATTTCAAGCACAACAACTTCTCAAGTTTTGTCCGGCAACTTAATACTTATGGGTTTCGCAAGGTTGATACAGATAAGTGGGAGTTTGGGAATGAAGCTTTCAAAAGGGGCAAAAGACATTTGTTGAAGAAGATCCAGAGGCGCAAGTCACCTCAATCACTGCAGGTTGGGCCTTCTGCCGAAGCAGGGAGGCCCAGACTGGAAGGTGACATTGAGACTTTGAGGAAAGAGAGAAGTATGTTAATGCAGGAAGTTGGTGACCTGCAGCAGGAACAGCTGGGTACAGTTCACCATATGAAAGTAGTGAAAGAGAGGCTTCAGTCTGCGGAGCAGAGACAGAAGCAGATGGTTTCTTTCTTGTCCAAGTTGCTTCAAAACCCAGCATTCTTAGCCCGCCTTCAACAGAAGACAGGACAGAAAGGTATAGACTCTCCAAGGATGAAGAGGAAATTTGTTAAGCAGCATCAGCATGAACTAGGTAAATCAGATTCTTGTATGCAAGGGCAGATTGTGAAGTACCAACCTGCTTGGAGAAATCTCTCTGTAGTCCCAGAGGTGAATCCAGTAGTTCCTATTGAACAATCTCCTGATAATCTTTCACAAGTTATGGCAGGAAAACTGGGTTTGGTTGCAGAAAGCAAGCCATATCAATTTGCGGATGTAGCATCAGATGAGTTAAATTTATCAGCTGAACCAGCAGTAATGCGAGGGGTTATCAAAACACCAGAGGAAGAAGGTGAAGGGGCGTCAAGCATGGGAGCTGAAGATCCATTTCAAAAAGGGAAGAGTGTTCTGAGCCCAGAACAGGAGGTTAATCCCGAGTATCATGTCTGTTTCCAGGAGGATTTTGGGAAGAATAAGATGTTTCCGGAACTTTTCTCTCCAGGGATCGATAGCATGATTAAACAAGAAGATATATGGAGCATGGGTTTTGATGTCAGTGCTGGTATGTCAAGTTCTAGCAATGAGTTATGGAGTAACCTGGTCAACTATGATGTGCCGGAGATGGGAGTGACAAGCGGATTGTTAGATATCTGGGATATAGGTCCCCTGCATGCAGCAGGAGGATCAGGCATTGATAAGTGGCCAGCTGATGAATCTGCATTTGATGAGCCTGATAGTCAAGCTGGCCAGCTGAAAGTCGATACATCTAAACGTATTGATCCATAG

>PmHsf7

ATGGATGAAGTTCAAGGTGGCGCCAGCTCGTTGCCGCCTTTCCTTTCCAAGACCTATGATATGGTGGATGATGCTTCAACCGATTCAATTGTGTCTTGGAGTGCCAGTAATAAGAGCTTTATTGTTTGGAACCCGCCGGAGTTTGCCAGAGATTTGCTGCCAAAGTTCTTCAAGCACAACAACTTCTCTAGCTTCATCAGGCAGCTCAATACATACGGTTTTAGAAAAATTGATCCGGAACAATGGGAATTTGCCAATGACGATTTTATCAGAGGTCAGCCAAACCTTATGAAGAATATCCATAGACGAAAGCCGGTTCATAGTCACTCTTTGCAGAATCTCCAAGTCCAAGGGAATGGGACTTCCTTATCCGAATCTGAAAGGCAGAGTATGAAGGATGAAATCGAGAGGCTTAAACATGAGAAAGAACGCCTTGGTGTGGAGTTACAGCGGCTTGAGCAGGAGCGGCAGGGATTGGAGTTGCAAATGCAGTTCTTGAAGGAACGTTTGCAACACATGGAAAGGCAGCAGCAAACCATGACCAGTTTCGTGGCCCGGGTTTTGCAGAAACCGGGGATTGCCTCCAATCCTGTTCCGCAATTGGAAATCCATGGGCGAAAGAGAAGGTTGCCAAGAATCGGTTGGTCTTATGATGAAGCGAGCAATGGAAATAATCAGGTGGCAAGTTCACAAGCTGGTATTAGAGAAAATGCGGATATGGAAAAGTTGGAGCAGTTGGAGTCATTCCTAACATTTTGGGAAGATACCATTTTTGATGTTGGAGAGGCTCATATTCAGGTTGTTTCAAACGTGGAGTTGGATGAGTCTACAAGTTGTGTCGAAAGCGCAGCTATATCTTCAATACAACTTAATGTTGACGCTCAGCCTAAATCCCCGGGAATTGACATGAATTCTGAACCTGCTGTAGTCGTTGCTCCTGAGCCTGCTGCAGCTGTACCTCCTGAGCCTGCTTCCTCAAAACAACAATCCTCAAAAGAACAAACATCTGGAATCACTGCTTCCACGCCAACTGGCGTGAATGACGTATTCTGGGAACACTTCTTGACAGAGAATCCTGGTTCAGTGGAAGCTCAGGAAGTTCAGTTGGAAAAAAGGGATTCTGATGGTAGAAAGAACGAAAGCAAACCTGGTGATCATGGCAAGTTGTGGTGGAATATGAGGAATGTAAATAACCTTACAGAACAAATGGGGCATCTTACTCCAGTAGAGAAAACGTGA

>PmHsf9

ATGGAGGGGACTCAAGGTGGTTCTAATGCACCAGCGCCTTTTCTTACAAAAACATATGACTTGGTGGATGACCCCTCATCCAACCGCGTTGTGTCTTGGAGTGAAACTGGTTGTAGTTTCGTGGTTTGGGATCCCACAGAGTTCGCCAAAGAAATGCTTCCCATGTATTTCAAACACAACAACTTTTCAAGCTTCGTGAGGCAGCTGAATACATATGGGTTTAGGAAGATTGATCCTGAACAGTGGGAGTTTGCAAATGAGGAATTCTTAAGAGGAGGAAGACATCTGCTGAAGAAAATTCACCGCCGCAAGCCAATTCATAGTCATTCCATGCAGAATCATGAGTATTCTTCAGTTCCTTTAAGTGAGACAGAAAGGGAAGAATATGAGAAGAAAATCAATAGACTGAATCATGATAAAAGCTTGCTTGAGTTAGAGCTACAAAGGCATCAAAGAGAGAATCAAGAGTTTGACTATCAAATACAGATATTACAAGAACAACTGCAGAAAATGGAACATCAGCAGAAACAGTATACATCCGTCTTGGCTCAACTACTGCAGAAACCAGGATTAGCTTCTATACTTATGCAAAAATCAGAAATCCATAACAAAAAGAGAAGATTGTTGACATCTAGCCACGTTACTGATGATTTCAAGATGGAAATTTTGAATTTTAATACGCAGAACGAAAACCTGGGTACAATTTCAACTCCCATAATAAAGTTGGACCAGCTTGAGAAGATGGAGTCTTCCTTAAACTTTTGGGAAGATTTTCTACATGGGATCGGGGAAGCAATGCCTGAAGAAGTAAATGATATTGGCTTGTTATCTCAGGCCTCTCCCATCATTGTTACAGAAATACAAGACCCTGGCATGAATAGCAGGCCTTGCTCACCTAGATCGCGTTTATCTTCGCCAAATTCAATGAATGTTTCATCCCCAGAGGTGGTTGGGTCTGCAAATTTTCTTGACATCCTTGCTATAACATCAACATGCCACAATGTTGATTTCAGGCCAAAATCTTCAGGGATTGATATGAACTCTAAGCCCGATACTGCTTCTGCGGCTGAGGCCTTGAAAGAAATGGTACAGGAAATGAAAAATGCCGAGCCTGCTGCGGCAAATGATATGTTTTGGGAACAGTGCCTGACAGAGACCCCTGGTTTAGATGATGCGCAGGAAGTACAGTCAGAAAGAGGGGATAGCGATGGCGGAGTGAGCAATGCCAATCCAGCAATTCAGAAAAAGCTTTGGTGGAACACAGATGTAGATAACTTCACAAACCAAATAGGGCGCCTCACTTCAGCTAGTTGA

>PmHsf4

ATGGAAGGAGCTTCGACGGCGGGCGGAGGAGGCGGTCCGGCGCCGTTTTTGCTGAAGACGTACGACATGGTGGATGATTCAGCGACAGACGAGATCGTGTCGTGGAGCACGAACAAGAAGAGTTTCATCGTTTGGAACCCGCCGGAGTTCGCTCGGCTTCTGCTCCCCACTTATTTCAAGCACAACAACTTCTCTAGCTTCATTCGTCAGCTCAATACATACGGATTTCGAAAGATTGATCCAGAGAGATGGGAATTTGCTAATGAAGACTTCATACAAGATCAAAAGCATCTTCTTAAGAATATCCACCGCAGAAAACCCATCCACAGCCACAGTAATCCTCAAGGTTCTATGGTAGATCCGGAGAGAGCGGCTCTTGATGACGAAATAGAGAAGCTTTCGCATGATAAAGCCACACTAGAGGCAAATATTTCAAGGTTCAAGCAGCAGCGATCTGATGCAAAGCTGCAGTTGGAAGACCTAACACAGCGGGTGAATTCCATGGAACAACGGCAGAAGGATTTGCTGAAATTCTTAGATAAGAATGTTCAGAACCCTACTTTTGTTGAACATCTCACTAGAAAAATCGAAGCTATGGATTTCTCAGCATGTAATAAGAAAAGGCGATTGCCTGATGTTGATCACCTACAGCCAGTTGTGGAGAATAGTTTTGTGGATAACCAAAGTAGTTCCAGATCTGAGTTTGGGAATATTTTCCACCAAGACTTTTCAAGTAAACTGAGACTAGAATTATCACCAGCTGTTTCAGACATTAACCTGGTTTCGCGCAGCACACAGAGTTCTAATGAAGATGGGTATAGTCCAACTAGGAAAATATCTGAAGAACTTAAAGGTGTACAGAAAAGAACAGAAGGCCTTTTATTTGCACCTGAAACCTTAGAACTTTCAGATACTGGAACATCTTTTGCATTCAAAATGGATTCGTTGTTATCACGAAAAGCACTAACTGTTGGGAACCCGAGACTTCATTCGCTGCAGCCAGGTTTGTCTTCTAACGAAGAAGGTGATGGCCAGATATCCTGCCAATTAAAGCTCACTCTAGCATCTTCCCCGTTGCAAGTCAATAGTAGTCCTCATTCTGCTACAATACCCCAAGTAGGTCAGGATATCAGCAAATCCCTAGCATCAGGATTAAATGCCATTGGCAAGGAATCAGATATAAGAGCCTTTACAAACAAAAATCCAGCTGATGAAGACATGCATAAAACTTGCTCCCAAGAAGCCACAAATAACAATCAAGGGCCTCCACCTGCTCCAGTTAGAGTAAATGATGTTTTCTGGGAACAGTTCCTAACTGAAAGACCTGGCTGTTCAGAAAATGAAGAAGCAAGTTCTAATTATAGGGGAAATCCATATGATGAGCAGGATGATGGAAGGCTAGGCCACGGAATGTCCAGAAGTGCCAAGGATGCTGAAACACTCACTCTTTGA

>PmHsf5

ATGAATTCTGATCAAGACCCAGTAAGTTTGTCGTCTTCAGGTGCGGCACCTGATCATCCTCTGCCAATGGAGAAACTTTATGACCAAGGCCCCCCACCATTTCTCACCAAAACTTATGACATCGTTGATGACCCAACAACCAATGATATAGTTTCTTGGAGCAGAGACAACAATAGCTTTGTTGTGTTGGATCCCCAGAAATTTTCCATGAGACTTCTCCCCAGATACTTCAAGCACAACAATTTCTCTAGCTTCGTCAGGCAGCTCAACACCTATGGATTTAGAAAGGTTGATACAGATAGATGGGAGTTCGCTAATCAAGGGTTTCTCAGAGGGCAAAAACATCTTTTGAAGAATATCCGGAGGAGGAAGACATCTTACCATCCTCAGGCTTCACAGAAAGCTTTGGACTCTTGTGTTGAAGTTGGAAAGTTTGGATTGGATGGAGAGATTGACCAGCTGAGGCGTGATAAACAGGTTTTAATGGGGGAACTAGTGAAGCTTAGACAACAGCAGCAAACTACTAGAGTTTACCTCCACGGAATGGAAAATAGACTGAAGAGGACAGAGATGAAACAGCAACATCTGAGGAATTTCTTGGCAAGAGCAATGCAGAATCCCAACTTTGTACAACAATTGGCACAACAGAAGGACAAAAGGAATGAACTCGAGGAAGCAATTAGTAAGAAGAGAAGGCGGCCTATTGAGCAAGGGCCTAGTAGTTTTGAGGTGGATGAATTAGGCCAAGTTGGAGTAGAAACTTTTGTTAAAGTTGAACCTCAGGAATACGATGACATATCTGATCATTTTGAAAATCCGGAGTTGGACACATTTGCTATTGACATGCAGGGTATAACTGGAAGCCAAAATGTTCATGATGAGGAAGAATGTATGGAGAAGGAAGAGGGAAATGAAAGTGGAAGCAAAGACCCGGGTAACAGTTTCTGGCACGAATTGTTGAATGAGAGTATTGATGAAGAAATTGGGATGCTAGGTGGTCAAGAAGAAGATGAGGATGTCGATGTGTTCGTCGAGGAGCTTGTTTACTTGGCCTCCAGTCCCAAGTAA

>PmHsf11

ATGAACTATCTGTACCCAGTGAAGGAAGAGTTCCCGGGTTCAAGTTCATCACAATCGGGTCCTGGTGACCCGGTGGTGATGATACCGCCACAGCCAATGGAGGGTCTGAATGACATAGGCCCTCCTCCATTTCTGACCAAGACCTTTGACATGGTGGATGACCCGAGTACCAATCGGATAGTTTCTTGGAGCAGAGGAGGTGGAAGCTTTGTTGTTTGGGATCCTCATCCCTTTGTTATGAATCTCCTTCCTAGATACTTCAAGCACAGTAATTTCTCAAGCTTTGTCAGGCAGCTCAACACTTACGGCTTTAGAAAGGTTGATCCTGACAGATGGGAGTTTGCCAATGAGGGGTTTGTAAGGGGTCAGAAGCATCTCCTTAAGAACATTAAAAGAAAGAAGACACCTTCTCAGCCTCTTCCTGCACAACAAGCTCTAGGCCCTTGTGTGGAAGTAGGGCGGTTTGGGCTAGATGGAGAAATTGATCGTTTGCGGCGCGACAAGCAGGTCCTAATGATGGAGTTGGTGAAGCTTAGACAGCAGCAGCAGAATACTAGAGCTTACCTTCAAGCAATGGAACAAAGGATACAAGGGACCGAAATGAAGCAGCAACAAATGATGGCTTTCTTGGCAAGGGCAATGCAAAACCCAGCTTTTATGCAGCAGCTAGTCCAACAGAAGGATAAAAGGAAGGAGCTTGAGGAAGCCATGACTAAGAAAAGGAGGAGGCCAATTGATCAAGGACCTAGTGGTGTTGGTGGTGGCAAATCGAGCCTAAAGGGCAAGGGAACAAACCTCATTAAATGTGAGCCTCTTGAATTTGGAGATTGTGATTATGAAATGTCAGAGCTAGAAGCACTTGCATTGGAAATGCAAGGATTTGGAAAGGCAAGAAAGGAACAGGATGAAGAGAGTGAGAGATTTGAGGGAGACTTAAGCATTCCAAGTGCAATAGTAGGGGAAGATGAAGATGTGATTATCTTGGCTGATCGCTTAGGTTACTTAGGTTCATGCCCAAAGTAG

>PmHsf3

ATGTTGAAATCGGCGGGGAAGAGTGGAGATGGGTCTGGGTCTGGTGGATCGGTGGCTCCTTTTCTGAGGAAATGCTATGAGATGGTGGATGATAACGATGCAGACTCTATAATCTCGTGGAGTGAAACCGGTGACAGCTTTGTGATATGGGACATGACCCAGTTCTCGATTTTATTGTTGCCCAAGTATTTCAAGCACAGCAACTTTTCTAGCTTCATGAGGCAGCTCAATATCTATGGCTTCAGAAAAATAGATTCAGATCGTTGGGTGTTTGCAAATGAAGGGTTTATTCGAGGTCAAAAGCATTTGTTGAAGAATATTTCTAGAAGGAAACATCCTCAGGGCACAGATCAGAGAAAAGCATTACAGCAGAAAGACAATCCTGATGGGCCTTTTGAAAACATTGTTGAAAATGGTCTATGGAGGGAAGTTGAGAACCTGAAGACTGATAAAGTTGCTCTGAAGCAAGAGTTGGTCAAGCTTAGGCAGCACCAGGAAATTTCAGAAAATAACTTGCTCCTCCTGAGGAACCGCCTTCGTGGAATGGAGAAGAATCAGCAGCAGATGCTGTCATTTCTAGTTATGGCCATGCAAAGTCCTGGGTTTTTAGTTCAGCTTCTTCAGCCAAAAGAAAACAGTTGGCGCATTGCTGAACCTGGAAATATGCTAGAACAAGGTGTAGATGATGGTATACCAATAACTTCTGATGGTGCGATAGTGAGATACCAACCTCCTGTGGATGAAGCCCCGAAGCCTATCCTCGCAGCGAATTCAGGCTCAGACAAACAAACTGAATTTGATTCTTATATAGATGGAATGAACGATTTTGTCGTGAATCCTGATTTCATGAAAATGCTAATGGATGAAAAGTTGAGCTCTCTGGAAAATCAAGCCCCATATACCCTACCGGATATATCTGATGATGGTGCATGGGAGCAGCTTCTTTTAGCTAGTCCTTTCTTAGAAGATATTGAAGCTACAAAGGAAGATGGAAAAGAGACTGTTGACTCTAGAATGGAGGTGGAATCAACCGCATCGGAGCTGCAAGAATCACAGAATTTTGATACTTTAATAGAGCAAATGAAGAAATCTCAGAACTTTGCATCGGAATCAACAGTTTATGGATCTAATGTGGAGAGCTCTCAAAACTTGGAACATATAACCGAACAAATGGGATATTTAGCTTCTGACTCTAACAGCAAACGTGGAACACAATCAGGAAAGTGA

>PmHsf15

ATGGCGCAAAGGTCCGTTCCGGCGCCGTTTTTGACCAAGACGTATCAGTTGGTGGACGATCCGAGCTGGGACGATGTCATCTCGTGGAACGAAAGCGGGACAACGTTTGTTGTTTGGAAGACTGTGGATTTTGCCAGGGATATGTTGCCTAAATATTTCAAGCACAACAATTTCTCAAGCTTCGTCCGCCAGCTTAACACCTATGGCTTTCGAAAGACGGTGCCGGACCAATGGGAGTTCGCGAACGACAACTTCCGGCGAGGGCAGAAGGAGCTCCTCGCCGAAATCCGTCGCCGGAAATCAGTGACGGCAGGGCCGGGGAAGGCTACTGCCAGCGAGAAATCCGGAGGGCCGTCGACTCCATCGAACTCGGGGGAGGAGATGGCGTCGACTTCGACGTCGTCGCCGGACTCGAAGAACCCGGGGGCGGTGGAGACGGCGGCAATGGGTCAAGCGTCTGATTTGTCGGGCGAGAACGAGAAACTGAAGAAGGAGAACGAGAATTTGAGCTCAGAGCTGGCGCAGACGAAGAAGCAGTGCGACGAGCTCGTGGGTTTCCTGATGGATTACCTGAAAGTGGGGCCCGATCAGATCAATCGCATCATGCGGCAAGGAAGCTATGTGTCCACCCGTGATGAAGATGAAAATGAAGATGATGATGCTGATGATGATGATGACGACGACGGAAAACAAAAAGAGGGCTTGAAGCTGTTTGGGGTTTGGGTGAAAGGGGACGAGAAGAAGAAGAGTAAGAGGACGGAGCGAGATGAGAAATTTGGGGTTGGTGTTGGTGGGACCTACGCGAAGAAGATGAAGAGAGCGGAATTCGGCGCGCCGATGTTGAAGAGGGGGAAGGTGTGCAACTGA

>PmHsf6

ATGGCTGCTACAACTTCTTCAGGCCAATCTCCGAGGACAAGAAGCCCTGCCCCTTTCTTGTCCAAGACATATGATTTGCTAGAAAAAGGTGCAGCAGAGGAAGGAGATAGCGGAAAGAAGATTGTGTCTTGGAATGCAGAGGGCTCTGGATTTATAGTTTGGTCTCCTGCTGAGTTCTCAGAGCTCCTGCTGCCTAAATATTTCAAGCACAATAATTTCTCCAGCTTCATCCGCCAGCTTAATACCTACGGGTTCAAGAAAACATCACCAAAACAATGGGAATTTAAGCATGAAAAGTTCCAGAAAGGCTGTAGGCATATGCTGGTGGAGATCACAAGGAAGAAATGTGAGCCAAGTGCATTTCCAGTGTATCTAAAGGCTTCAGAAGAGAGTGGTAGCAGCAGTACAACTGTGGCTGCAGCAGAGGAAAATAATCGCTTGCTGCTAATGGAGGAGAACAAGAACCTCAGGAAACAGAAACTGGAGCTGCAGATGCAACTATCTCAGTTTAAAGCCTTAGAAATGAAGCTGTTGGATTGCCTAGCGCAGAACATGGAAGATCATCAGAATAAAGTTCGATGCTGA

>PmHsf10

ATGGCTCCGACGTCGGTGGAGCCGAACGGCGGTGAGTCCACATCCAGCGAGTCATCCCATAGAGCTTTACCGACACCATTTCTGACCAAGACGTATCAGCTGGTCGATGATCCCACAATCGACGACGTCATCTCCTGGAACGACGACGGATCTAGCTTTGTCGTGTGGAACCCCACCGTCTTCGCCAGAGATTTGCTCCCCAAGTATTTCAAGCACAACAACTTCTCTAGCTTCGTCAGGCAGCTCAACACCTACGGATTTAGAAAGGTTATACCAGACCGTTGGGAATTCTCGAACGATTGCTTTCGAAGAGGCGAAAAGCGGCTTCTCTGCGAGATACAGCGCCGAAGAATCATGCCTCCGGCGCCGGCGGTGGCCGTTTCGCCGATGGCGACGGCAGCGGTGGTTCCGAATGCGAAACCTATGATATCTCCGTCGAACTCCGGCGAGGAGCAGGTGATTTCGTCGAGCTCGTCGCCGATCAGAGCTCCGTCCGAGCTCATGGACGAGAACGAAAAGCTGAGGAAGGAGAACATGCAGCTCACCAAGGAACTGGCTGATGTCAAGTCTCTCTGCAACAACATCTTCAGCATGGTCTCGAATTACGCGTACGCACAATCGGAAAGCGGTTTCCCATATGTGAAACCGCTGGATTTGATGCCTGAGAAGCGGTTCTCCGGCGACGGCGAGAAGGAAGAAGAGGAGGCGAGCCCGAAGCTTTTCGGTGTGGCGATTGGAGCCAAGCGAGCAAGAGAGACCGTCGGCGACGGTGTAGAGGAGGATGAGACCGGCTTACGACTGCAGCAACCGAGTGGTGGTGGCGACGTTAAATCAGAGCCGTTAGATGTGGATCGTCAGGAAACGCCGTGGCTGAATCAGCGCCACATGGCTAATCAGAGGGTGTGTAATTAA

>PmHsf13

ATGGCGTCGTTGCAGGCCGACCAGAACGGCGACTCCGGCCCCGTTAACGGCGCTGGAGGAGGTGGAGACTCACAAAGGACGCTTCCGACGCCGTTTTTGACCAAAACGTATCAGCTGGTGGACGATCCGTCCGTCGACGATTTGATCTCGTGGAGCGAAGACGGATCGGCCTTCATAGTCTGGCGACCCGCCGAATTCGCCAGAGATTTGCTCCCCAAGTATTTCAAGCATAACAACTTCTCCAGCTTCGTCCGCCAGCTCAACACTTACGGATTTCGGAAAGTTGTGCCGGATCGGTGGGAGTTCGCGAACGATTGCTTCAAAAGAGGTGAGAAAGGCCTCCTACGGGAGATTCAGCGCCGGAAAATCTCGCCGTCGGTGTCGGCGTCGCCTGCGGCGATTACCGCGACGTTGGCGACGGTGTCTGCGGTGGCTCCCGGGGTATCTCCGTCGAACTCCGGCGACGAGCAAGTGATCTCGTCGAACTCGTCGCCGGTGGCGCCTCCGGCTACGATGTTGAGCCGAATCCGGAGCTGCACCACGACGTCGGACGTTCTGGAAGAGAACGAGCGGCTCAGGAAAGAGAACATGCAGCTAAGTCACGAGCTGACTCAGCTGCGCGGCTTGTGTAACAATATATTGGCATTGATGACGAACTACGCTTCTGGTCAGTTGGAGGGCGGCGGCGGTGGCGGTGGCGGTGGGAGTGTAATGGATGAGGGTAAGCCTCTGGAGCTGTCGCCGGTGAAGGAGGCTGAGCCGTCTGAAAACGGCGTCGTTCGCGAGGGGTCTAAGGCGGAGGCTTCGGCGGAGGAAGAGGAAGACGAGGAGGAGATGAGGCCGAGGCTGTTTGGGGTGTCGATTGGGGTGAAGCGCGTGCGGAGAGACGAGGAGGAAGAGGAGCAGCATCGGGAAGGGTCTGAGGCGATGAAATCAGAGCCGTCAGATGGGAGTTCGAAGCGCGACCAGGACTCCACGTGGCTGGAGCTCGGAAAGTGA

>PmHsf18

ATGGAGGGTGTGTGTGACCAGAAGGGTTTGCTGGAATATGTGAGGAAGTCAAGCCCTCCACCTTTCTTGTTGAAGACCTACATGCTGGTGGAGGATCCTGCCACCGACGATGTGATCTCCTGGAACGATGACGGGTCGGCGTTCGTGGTGTGGCAGCCGGCGGAGTTTGCCAGAGATCTCCTCCCAACACTCTTCAAGCATAGTAACTTCTCTAGCTTTGTCAGGCAGCTAAATACTTATGGATTTCGGAAAGTTTCAACAAGCAGGTGGGAGTTCTGCAACGACAAGTTCCGAAAGGGTGAAAAGGATCAGCTATGTGAAATCCGCAGAAGAAAAGCATGGGCCAGCAAGCAACAGCCAATCAACAACATTGCTCTAAACCAAGCTGCACAAGCAATGCCAAATCAAGACGAGTTTGATGAAGACCAGAGATCAAACTCCTCAACCTCCTCCTCATCTGATTACAGCTCTCTCGTCGACGAAAACAAACGGCTGAAGCAGGAGAATGGGGTTTTAAGCTCAGAGCTCACCAGCATGAAGAGGAAGTGTAAGGAGCTTCTTGACTTGGTGGCCAAGTGTGGAGACTCAGCTGAGAAAGAAGAGGAGAATAGTGAGAGGGTGCCCAAGTTGTTTGGAGTGAGATTGGAAGTGGAGGGGGAGACGGAGAGGAAGAGGAAGAGAGCTGAAATTAGTGAGAGCGCAAGCATTTTACTATCTCAAGCATGCAAATAA

>PmHsf1

ATGGCTCTCATGATGGACAATTGTGAGGGCATATTGCTTTCCCTGGACTCGCACAAGTCGGTGCCGGCTCCCTTCCTGACCAAAACGTACCAGCTCGTGGATGATCCAGCCACCGACCACATCGTCTCGTGGGGAGAGGACGACGCCACCTTCGTCGTTTGGCGCCCTCCCGAGTTCGCCCGGGACCTCCTCCCCAACTACTTCAAGCACAACAACTTCTCCAGCTTCGTCCGCCAGCTCAACACCTACGGTTTTAGGAAGATTGTACCGGACAGATGGGAGTTTGCGAACGAGTTCTTCAAGAAAGGAGAGAAGCATTTGCTCTGTGAGATCCATAGAAGAAAGACAGCTCAGCCTCATCAGGTGGGTTTCAGCCACCACCACCACCACCACCACCACAACCCGCATTCGCCACTCGGCATCAACGGCCACCATCATCCGAGCTTCTTCCCCTTCCCGAGTAGTGGCAGCATCTCCCCCTCCGACTCGGACGAGCCGCCCAACTGGTGTGACTCGGACTCACCACCACTCCCATCCCCAACCGGAGGTATTAACAATCACAACAACAACAATAATAATTTTATGAATATTAATAATGCGTCGGTGACGGGCTTGGCGGAGGACAATGAGAGGCTGCGGCGGAGCAACTCCGTGCTGATGTCAGAGCTAGCCCACATGAGAAAACTCTACAACGACATCATCTACTTTGTTCAGAACCATGTCAAGCCTGTGGCTCCAAGCAATTCCTACCCTTCTTCTTTGCTTCTCTCTAACCCTCCTCCGAATTCCATGGCTCCAGCTGCTACTGCTACTAAGCCTAGTAATTTCAACCAGCTTCTTGGGTACTATCCAGCTCCTGCTACAAATGCTAAGCAAACCCCTCACATGTCTACGACGATCCACCATCATGTTATGAACTCTTCCAGCCCGAGCAACACCACGTCCAAGAGCAGCTCAGTGACTATTCTTGAAGACCAGCAACAACCCAGTAGCAATGGGTGCAAAAATACCAAGCTGTTTGGGGTGCCGCTGCTTCACTCGAAGAAGCGGTTGCACCCGGAGGAGTATGGCTCGAACCATGGGACCAGCATGATGGAGGCCAGCAAGGCTCGTCTGATTTTGGAAAAAGATGACTTAGGTCTCCATCTCATGCCTCCCTCCGCATGTTAG

>PmHsf17

ATGATGATGGAGGAGAACAACAACGTCATCGCGCCGTTCGTTATGAAGACTTACCAGATGGTCAACGATCCGACGACGGACAAGTTAATCTCTTGGGGCCAAGCCAACAACAGCTTCATCGTCGTCGACCCTTTAGACTTCTCTCAGAGGCTCTTACCCGCTTACTTCAAGCACAACAATTTCTCCAGCTTCGTTCGCCAGCTTAACACATATGGATTTCGAAAGGTCGATCCAGATAGGTGGGAATTTGCGAACGAGTGGTTTCTGCGGGGCCAAACGCATTTGCTGAGGAACGTGGTGAGGAGAAAGCACATGGGTAAGAATTCGTATTCGAATTCGAATTCGACCACGTGCTTATTACAGGGGAAGCACGAGGAGCTTGACGACGAAGAGATAGTGATGGAGATTGCGAGGTTGAAGCAGGAGCAGAAGGCGTTGGAAGAAGAAATGGAGAACATGAACAAGAGATTGGAGGCAACTGAGAGACGACCCCAGCAAATGATGGCTTTTCTGCACAAAGTCGCGGAGGACCCTGAGATTTTGCCACGTATTATGCTCGAGAAGGATCGTACGTTCAGGGCGCAGTTGGGGGAGAAGAAGCGGCGGGTTATGATGATAACCTCAACGTCGTCGTCTTCGTTGGGCATGGGGGCCACCAACTCCGTCGAGACCGAGGATGAGGATGACGGAACCGTAGGGGTAATTTCGTCATCTCCCGAACCGGGTTTTGAGATGGATAGTTTTTATTCCACGTCTCCAGAGACGTCGACGGCCCGAGAGTGGGGGAGGCAGAGGCGGGGCGGGGGGCTGGGTCGGGCTGTGCAAGACCCGTATAATATGAACCCGACGGTGTCGGGTCATGGGATCGGGAATACTAGTAATTCCGGGTACGGGTATGGGAATAGAAATGGTGGTGCGGAAGTGGGTTATCTCACAGAGGAACCTACCCCTGCACCACCGCCTTATCCATTCTCGTTGTTAGAGGGTAGCTTTTAG

>PmsHsp1

ATGTCGATCATCCCCAGCTTCCGACGAGGCAGCATTTTCGACCCTTTCTCTCTCGATGTCTGGGAGCCATTCAAAGATTTCCCATTCCCTTCCTCCTCATCACTCTCCACATTCCCTGAATTTTCCCGGGAAAATTCAGCTTTCCTGAACACGAGGATCGACTGGAAGGAGACCCCGGAAGCCCACTTGTTCAAGGCAGACCTTCCGGGGCTGAAGAAAGAAGAAGTGAAGGTGGAGGTTGAAGACGACAGGGTGCTTCAGATCAGCGGAGAGAGGAACGTAGAGAAGGAGGACAAGAACGACAAGTGGCACAGAGTGGAGCGCAGCAGCGGCAAGTTCTTGAGGAGGTTTCAGCTTCCTGAAAATGCAAAGGTGGACGAGATTAAGGCTGCAATGGAGAATGGGGTTCTGAGTGTCACTGTTCCAAAGGCAGAGGTGAAGAAGGCTGATGTCAAAGCCATTGAAATCTCAGGTTAA

>PmsHsp3

ATGTCGATCATCCCCAGCTTCCGACGAGGCGGCATTTTCGACCCTTTCTCTCTCGATGTCTGGGAGCCGTTCAAGGATTTCCCGTTCCCTTCCTCCTCATCACTCTCCACATTCCCTGAATTTTCCCGGGAAAATTCAGCTTTCTTGAACACGAGGATCGACTGGAAGGAGACCCCGGAAGCCCACTTGTTCAAGGCAGACCTTCCGGGGCTGAAGAAAGAAGAGGTGAAGGTGGAGCTCGAAGAAAACAGGGTGCTGCAGATCAGCGGAGAGAGGAAAATAGAGAAGGAGGACAAGAACGACCAGTGGCACAGAGTGGAGCGCAGCAGCGGCAAGTTCTTGAGGAGGTTTCAGCTTCCTGAGAATGCAAAGGTGGACGAGATTAAGGCTGCAATGGAGAATGGGGTTTTGAGTGTCACTGTTCCAAAGGCAGAGGTGAAGAAGCCTGATGTCAAAGCCATTGAAATCTCTGGTTAA

>PmsHsp5

ATGTCGATCATCCCCAGCTTCCGACAAGGCAGCATTTTCGACCCTTTCTCTCTCGATGTCTGGGAGCCATTCAAAGATTTCCCATTCCCTTCCTCCTCATCACTCTCCACATTCCCTAAATTTTCCCGGGAAAATTCAGCTTTCCTGAACACGAGGATCGACTGGAAGGAGACCCCGGAAGCCCACTTGTTCAAGGCAGACCTCCCGGGGCTGAAGAAAGAAGAGGTGAAGGTGGAGGTTGAAGACGACAGAGTGCTTCAGATCAGCGGAGAGAGGAACGTAGAGAAGGAGGACAAGAACGACAAGTGGCACAGAGTGGAGCGCAGCAGCGGCAAGTTCTTGAGGAGGTTTCAGCTTCCTGAGAATGCAAAGGTGGACGAGATTAAGGCTGCAATGGAGAATGGGGTTCTGAGTGTCACTGTTCCAAAGGCAGAGGTGAAGAAGCCCGATGTCAAAGCCATTGAAATCTCTGGTTAA

>PmsHsp6

ATGTCGATCATCCCCAACTTCCGACGAGGCAGCGTTTTCGACCCTTTCTCTCTCGATCTCTGGGAACCCTTAAAGGATTTTCCATTCCCTTCCTCCTCATCACTCTCCACATTCCCTGAATTTTCCCGGGAAAATTCAGCTATCCTGAACACCAGAATCGACTGGAAGGAGACCCCAGAAGCCCACGTGTTCAAGGCAGACCTCCCGGGGCTGAAGAAAGAAGAGGTGAAGGTGGAGGTTGAAGACGACAGGGTGCTTCAGATCAGCGGAGAGAGGAACGTAGAGAAGGAGGACAAGAACGACAAGTGGCACAGAGTGGAGCGCAGCAGCGGCAGGTTCTTGAGGAGGTTTCAGCTTCCTGAGAATGCAAAGGTGGACGAGATTAAGGCTGCAATGGAGAATGGGGTTCTGAGTGTCACTGTTCCAAAGGCAGAGGTGAAGAAGCCTGATGTCAAAGCCATTGAAATCTCTGGTTAA

>PmsHsp7

ATGTCGATCATCCCCAACTTCCGACGAAGCAGCATTTTCGACCCTTTCGCTCTTGATCTCTGGGACCCCTTCAAGGATTTCCAATTCCCTTCCTCCTCATCACTCTCTACATTCCCTGAATTTTCCCGGGAAAATTCTGCTTTTCTGAACACTAGAATAGACTGGAAGGAGACCCCAGAAGCTCATGTGTTCAAGGCTGACATTCCGGGGATGAAGACGGAAGAGGTGAAGGTGGAGGTTGAAGACGACAGGGTGCTTCAGATCAGTGGAGAGAAGGATGACAAGAACGACAAGTGGCACATGGTGGAGCGCAGCAGCGGCAAGTTCTTGAGGAGGTTTCAGCTTCCTGAGAATGCCAAGGTAAATGAGATTAAGGCTGCAATGGAGAATGGGGTTCTGGGTGTTACTGTTCCCAAGACAGAGATGAAGAAGCCTGATGCCAAAGCCATTGAAATCTCTGGTTGA

>PmsHsp4

ATGTCAATCGTCCCAATCAACGAGCAACGAGGCAGTGACTTCGACCCTTCCTTGGCTCTTGACCTGTGGGACCCATTCACAGATTTCCCTTTCCCATTCCCTTCCTCACTCTCCAACGTTTTCCGGGAATTCAATCTGGGTTCTTCGGTAAACTCAAGACTAGATTGGAGAGAGACCCGAAATGCCCACATCCTAAAGGCGGCTCTTCCTGCGTTCATGAACGAGGACGTGCTTGTGGAGCTTCAAGACGAGCGTGTGCTTCAGATCAGTACAGACAGCGGTAGCTTCATGACAAAGTTTAAGCTTCCGGACAACGCCAAGATTGAGCAGCATAAGGCCTTCATGAGCAATGGGGTTCTCACTGTCACTGTTCCTAAGGAGGAGCCCAGCAGGCCCAATATCAGAGCCATTGAAATTTCTGGCGAAGATTAA

>PmsHsp8

ATGTCAATCGTCCCAATCAACGAGCAACGAGGCAGTGACCTCGACCCTTCCTTGGCTCTTGACCTGTGGGACCCATTCACAGATTCCCCTTTCCCATTCCCTTCCTCACTCTCCAACGTTTTCCGGGAATTCAATCTGGGTTCTTCGGTAAACTCAAGACTAGATTGGAGAGAGACCCGAAATGCCCACATCCTAAAGGCGGCTCTTCCTGCGTTCATGAACGAGGACGTGCTTGTGGAGCTTCAAGACGAGCGTGTGCTTCAGATCAGTACAGACAGCGGTAGCTTCATGACAAAGTTTAAGCTTCCGGACAACGCCAAGATTGAGCAGCTTAAGGCCTTCATGAGCAATGGGGTTCTCACTGTCACTGTTCCTAAGGAGGAGCCCAGCAGACCCAATATCAGAGCCATTGAAATTTCTGGCGAAGATTAA

>PmsHsp2

ATGGCTTATCTCTCAGTTCTCTATTTAAAGTTACGTTTACTTTTGTTTCTGAATTACAGTCTTTTGATACCTTATCATGACTCTCCGACAGGCGGTACTATTTGTTTTAAAATCCTCCTCTCCACATTTCCCGAATTTTCCCAGGAAACTTCAGCTTTCTTGAACACGATGATCAACTGGAAGGAGACCCCAGCAGCCCATGTGTTCAAGGCATACCTTCCGGGGCTGAAGAAAGAAGAGGTGAAGGTGGAGCTCGAGGACGGCATGGTGCTTCAGATCAATGGAGAGAGAAATGTAGAGATGGTGGACAAGAACGACAAATGGCACAGAGTGGAGCACAGCAGCGGCAAGTTCTTGAGGAGGTTTCAGCTTCCAGAGAATGCAAGGGTAGACGAGATTAAGGCTGCAATGGAGAATGGGGTTCTGAGTGTCACTGTTCCCAAGGCAGGGCCAAAGCCATTGAAATCTCTGATTTTGGAGTGTCAGTTGTCATCAAAAACCACAGGGACACGCATGCCCCAACAGGGTCAAAGCTTTCTGATTAATGCAATCAATAATAAGTTTAGAATTTCCTTCAACAAGAACTTTGCGCCATCCTCTATGAGTTCCATAAGCAAGGCCATCCCGTAA

>PmsHsp9

ATGGCACTAATTCCTCGTACCATTTTTGTTGGTCACTATGATCCTTTCTGCCACGATGTTTGGGACCCATTTCAGGAATTCCACTATGGATTTCCAAGAGAAGCAACCTCCTTCACCAACTCCATGATTGATTGGAAGGAGACCTCAGATGGGCGCGCGTATGTACTCAAGGAAGATCTTCCAGGCTTCAGGAGAGAAGAAGTAAAGGTGGATGTGGAGGAGGGTAGGGTTCTTCGCATTAGGGGGGAGAAAAATGTGGAGAGAGAAGAGAAGAAGGACCATTGGCACCGCATCGAAAGGAGCAGCGGCAAGTTCATCAGGCGTTTGAGTTTGCCTGAGAATGCCAAGGCTGACAAGATGAAGGTGTTCATGGAAAATGGAGAGCTCACTGTCACGGTTCCTAAGGAGAAGGTTAATTTCTATCCTCATGCAACCAGAGCCGTTCAAATTTCTGGGCACTAA

>PmsHsp16

ATGTCTCTCTTGCAGTCTCTCTTGGACCAACCCAACTTCTTGTCTCCTCTCAGAGTTTTTAACTCAGACATAGGCTACAACAACACCTACATGGACTGGAAGGAGACTTCACATGCCCACATCTTTGAAATTGATCTTCCAGGCCTTACAAAAGAGGATGTGAAGCTTGAGGTGCATGAAAATAGAGTGCTTCATGTGAGTGCAGAGAGAAAGGCAGAGCCTGAAGCAGAGGACCCCAAGAATGAAACGTGGCACTGCAGGGAAAGGACAAGTGACAGCTTCTCTAGAAACTTTCGGCTGCCTGAAAATGCCAAGGTTGATGAGATTAAGGCTTCCATGCGTGACGGGGTTTTAGTAATTACAGTGCCTAAGGAGGATGACTTGAAGAAAAAACACAAGCATCACAAGAAGGTTGAGATCTCTGGAGATGATGAAAAACATGGCTCCAAAGGGCTCGGACGCTTTGTGTGCTGCAAAGCTTAG

>PmsHsp18

ATGGCTGAGTGCACTAGGGACAACTCATTGGCATTGCTTTGCAATCTATGCACATGGCTACGGAAATTTATATTCTCTGACTGCCCTTTAAAGAAATGGGGTGTTCTTTTCCCGGCGGTGGCGAACACGCGGATCAACTGGAAGGAGACGCCGGAGGCGCACGTGTTCAAGGCGGACCTTCCGGGGCTGAAGGAGGAGGTGAAAGTGGAGGTTGAGGAAGGAAGGGTGCTGCAGATCAGCGGAGAGAGGAGCAGAGAGAAGGAGGAGAAGAACAAGTGGCAAAGGGTGGAGAGGAGCAGCGGCAAGAAGTTGTTTTCTCAACTGCCAAAGCAATTAATACCAGTTCAGTTAGCCAACGATTCAAACACTCTNNNNNNNNNNNNNATTCCAAGCGTATTCGGCGGCCGAAGGACTAACGTCTTCGACCCATTCTCTCTCGACATCTGGGATCCATTCCAGGACTTCCCGTTGATCAGCGGCGGTAGCAACGCAGCCTTGTCAGGCCCGCGGTCGGAGCTGGCGAGCGAGACGGCGGCGGTGGCGAACACGAGGATCGACTGGAAGGAGACGCCGGAGGCGCACGTGTTCAAGGCGGACCTTCCGGGGCTGAAGAAGGAGGAGGTGAAGGTGGAGGTTGAGGAAGGAAGGGTGCTGCAGATCAGCGGAGCGAGGAGCAGAGAGAAGGAGGAGAAGAACGACAAGTGGCACAGGGTGGAGAGGAGCAGCGGCAATTTCCTGCGGCGGTTCAGGCTGCCTGAGAACGCGAAGGTGGATAGGGTGAAGGCTAGTTTGGAGAACGGGGTGTTGACTGTGACTGTGCCCAAAGAGGAGGTGAAGAAGCCTGAAGTTAAGGCCGTTGCGATTTCTGGCTAA

>PmsHsp19

ATGGCTCTCAGCATCTTTGGTGGCCGACGAAGCAACGTCTTCGATCCGTTTTCTCTTGACATCTGGGACCCCTTTGAGGGCCTTGGCACTCTGGCCAACATCCCACCCTCTGCTCGCGAAACGACTGCCATCGCCAGCACTCGCATTGACTGGAAAGAGACCCCAGAAGCCCACATCTTCATAGCTGACCTCCCGGGGTTGAAAAAGGAGGAAGTGAAAGTTGAGGTTGATGACGGGAAGGTGCTTCAGATCAGCGGAGAGAGGAGGAGAGAGCAGGAGGAGAAGAACGACAAATGGCACAGGATTGAGAGGAGCACCGGCAAGTTTTCGAGGATGTTCAGGTTGCCGGAGAATGCGAAAATTGATCAGGTCAAAGCTAGCATGGAGAATGGGGTTCTCACCGTGACTGTGCCCAAGGAGGAGGAGAAGCGGCCACAGGTCAAGGCCATTGACATATCTGGCTAA

>PmsHsp22

ATGGAAACCAAGGTTGCAGCAAACGATGAGCAGTGTTACGAGGTCTTTGAACCGTTTTGCCGGTGGAAGAAAGAGGAAGGACTTGACATTCTTGAGGTTCATCTACCAGGTTTCAAAAGACAAGATGTCAGAGTTCAAATGAACAACAAGGGCATCCTAACCATTAGTGGAAAGCAATCTATGGAAGAAGAAACTGCATCTCCACCCAGCCGCTTCCTCAAAGAGATCAAAATTTCTACAAATTGTAATACGAGTGGAATCCGTGCTAAGTTTTCGCATGGAATTCTTTCCATATCTATGCCTAAGAAAGTGCCAAACCTTTCAACACAATTATCAGGCAGCGGAGACAAGATTAAGGCTGCTGAAATTGCTACATGGTCGGCAATTAATTACTATTTACTTGGTTTAAGAAGCAAAATTTTAAGCAAGGATATGGTTCTGAAGATGGCGGGGGTCGCTCTGGGAATGGCTCTTGGAGGTTATGCAATATATAGATATCCAAAGTCAGCTTGTGTCCAAAACTGA

>PmsHsp11

ATGGACGTGAGAATCGCGGGTTTGGATTCCCCACTCTTCTCTACACTGCAGCACATCATGGACTTCAACGACGAGCCTGACAAATCGTTCAACGCCCCCACCCGCACCTACGTCCGCGACGCCAAGGCCATGGCCTCCACGCCGGCGGACGTCAAGGAGTATCCCAACTCCTATGTCTTCGTCGTGGACATGCCAGGCCTCAAGTCCGGAGACATCAAGGTCCAGGTGGAGGACGATAATGTGCTTGTGATCTCCGGCGAGAGGAAGAGGGAGGAGGAGAAGGAGGGGGCTAAGTATGTCAGGATGGAGAGGAGGGTCGGCAAGTTTATGAGGAAGTTTGTGCTGCCTGAGAATGCCAATCTAGAAGCCATTTCTGCTGTTTGCCAAGATGGGGTCCTGACTGTGACTGTGGAGAAGCTGCCTCCGCCTGAGCCAAAGAAGCCCAAGACCATTGAGGTCAAGATTGCTTGA

>PmsHsp12

ATGAGTATGGAGGTGAGCCTGAGAAACATGGGGTTCGAGCCAAACCTGCTCGAGACTCTGCACGACCTACTGGACTTCTCCGACGAACAGAACCAGTCCAGCCACCATGCGCCTTCGCGCCAGTACGTGCGCGAGGCCAAGGCCATGGCTGCCACGCCAGCCGACATCAAGGAGACTCAAAACGCCTATATTTTTGTTGTGGACGTGCCCGGGCTGAGGCCGGATATGGTCAACGTTCAAGTTGAGGACGACAACGTGCTGGTGGTGAGCGGGGAGAGGAGGAGAGAAAAGGAGAAGGATCAGGGTATTAAGTATTTGAGGCTGGAGAGGAGGCTTGGCAAGTACCTCAAGAAGTTTGTGCTGCCTGAAAATGCTGATATTGAGAAGATCTCTGCCGAGTGTCAGGACGGGGTATTGACTGTGAGTGTGGCGAAGAAGCCGCCGCCTGAGCCTAAGAAGCCCAAGACTGTTCAGGTCCAAATAAGCAGCGGCCAAGGCAGCGGTCAGGGCAAACAAATCGGCCAAGGCGGTGGTCAGGGTGGCGAAGGAGGACATGGAGGTGGTCAGGGCGGCGAAGGAGGACATGGAGGTGATCAAGGCGGCGGGCAGGGAGGCCAAGGTGGCGGACAAGGGGGCCAACATGGCAGTCAAGGTGGACAAGGTGGGGGCCAAGACAGGTAA

>PmsHsp13

ATGGATTTGAGAAACGCGGGCAATGTCTTCGCGTTCTTGGAAGACATGCTAGACTTGGCAGAGCAAGAGCCCGAAAAGCCCCGAAACAACAACCACCCATCTCGGGCGTACGTCCGAGACGCGAAAGCGATGGCGGCGACGCCGGCGGACGTGGTGGAGTACCCGAACGCGTACGTGTTCGTGGTGGACATGCCGGGAATCGAGGCGGGACAAATTAAAGTGCAGGTGGAGAACGACAACGTTTTGGTGCTGAGCGGAAAGCGAAGGCGGGAGGAAGGGATCAAGGAGAGTGGGGTGAAGTACGTGAGGATGGAGAGGAGGGTTGGGAAGTTCATGAGGAAGTTTGTGCTGCCGGAGAATGCCAATTTGGATGCGATTTCGGCGGTGGCTAAAGATGGGGTTCTCACTGTGAGGGTGGAGAAGCTGCCTCCGCCGGAGCCCAAGAGGCCCAAGACCATCCACGTCAATGTTGCTTGA

>PmsHsp17

ATGAGCAGAGTAGCCGATTCGAGTGCCTTCAATGGGGATTTTGCAACAGCAGTGAACCACCTGCTCAATTTCCCTGAAACCATTGACAAGTTCATGCTCCCTTCTCGGGCTCACGAAACCAACAACGAGAACAAAGGAGCGGCTAGCATTCCAGTGGACATTTTGGACTCTCCCAAGGAGTATATATTTTTCTTGGACTTGCCTGGCTTGTCCAAATCTGATATTCAGGTAACAGTTGAAGATGAAAATACTCTGGTGATCCGAAGCAATGGGAAGAGGAAACGTGAAGATGGGGAGGAGGAAGGCTGCAAGTACCTGAGGCTTGAGAGGAGAGGACCCCAGAAGCTGTTGAGGAAGTTTAGGTTGCCCCAAAATGCTAATGTAGGAGCCATATCTGCCAAATGCGAAAATGGGGTTCTCACTGTGGTTGTTGAGAAGCTTCCTCCACCTCCCAAGCCCAAAACAGTTGAAGTTTCTATCTCTTGA

>PmsHsp10

ATGCCTAGGAGGATAGTGATGAGGATGGTGGCCTTCTTAGGCCTCCTCCTCATGCTCATGGCCACCACCATCAAAACCCATGCCTTAGTTCCATACCCAACAAGACCATCATCACTAAGTCTATGGGACATGACCGATGACCCCTTCAGAATTCTCGAACAAACCCCCTTCACCATCCCCATAGACGTCTCAGCCGCCGCCCTCCAAGACACGCTCGCTTTGGCACGTGCCGACTGGAAGGAGACGGCCACTGCGCACGTGATCACATTGGACATTCCGGGGATGAAGAAGGAGGACGTGAAGATAGAGGTGGAGGAGAACAGGGTGCTCAGGATCAGCGGAGAAAGGAAGATGGAGAAACAAGGGGAGGGTGACAAGTGGCACAGAGCTGAGAGGACAAATGGCAAGTTCTGGAGGCAGTTCAGGCTGCCTGCGAATGCTGACGTGGATCAGATCAAGGCTCATCTTGAGGACGGGGTGCTCAGAATTACGGTGCCAAAGTTTGCTGCGGAGAAGAAAAGGCAGCCTAAGCTCATCGACATTGCTCAACACACTACTTCTGATGATGATGCGGATATCAAGGCTGCCAAGGTCGCATGA

>PmsHsp15

ATGGAGCTCCCAGCCTTCCACACTTACCAATATGTCTTCCCTTCTCATCTCCTTTATCCATATCACTTGGCCCCTGAAAACTACGTCCACTGGACTGAGACCCCAGAGTCTCACATTTTCTCTGCTGACCTCCCTGGTGTTAGGAAAGAGGAAATAAAAGTAGAAGTAGAGGATTCAATATACCTCATAATTCGAACACAGAGAATTGATGAAGCCACAGAGCCCAGCAGGAGCTTTATGAGGAAGTTTCGGATTCCAGGTAGGGTTGATCTCGAAAGGATTTCAGCTGGATATGAAGATGGAGTATTGACAGTCACAGTGCCAAGATCTTTAAGGAGGACTTTTTATATTGACCCAGCTGATGTCCCAGAACGGCTTGAAGTTCTTGCAAGGGCTGCTTGA

>PmsHsp24

ATGCTCTACTTTTCTCAAAAGTGCTTCTTCTCCCACACTATAAGAACCAACAAGCCTCAAACCTCTCTCCACCACACACTCAAAAGTTCAGTTTCTCTAATCTTCAATTCAATGACTACTACTCGCAAGCAGCTTGAAGTTCTAACAGATGATCAAACTCCACACAAATGGTGTGTTTTGTTGAGAGAAGATGTGTTCAAGAAGTTCATGTCTCAAGGCAGTCCAGCAGTGCACAAGGTTTTTGGTGGAGGATCATTGTTCAGTCCATTCTTGTTTGGGAAATTTTTTGATCCTTCTGATGCCTTCCCACTGTGGGAGTTTGATGCAGATATCTTGTTGGCTGGTCTCAGAAGCTCTGGTCAAAGCAGTACTGTTGACTGGTTTCAAACAGATCAAGATTATGTACTAAAAGCAGATCTACCAGGAGATGGGAAAAACAATGTTCAGGTCTATGCAGAAAATGGGAAGGTTGTGGAAATTAGTGGGCAGTGGAAGCAGCAAGGAGGAGAGTCCAAGAGCACAAAAGATTGGAGAAGTGGGAATTGGTGGGAACATGGGTATGTTAGGAAGCTTGAGCTCCCACAAGATGCAGATTGGAGAAGAATAGAGGCCTCTGTGACTAATGACCTACTTTTAGAAATCAAAATCCACAAGATCAACCCCTTGGATTGTGATATTAGTCATCTGACCCTGAAAGATAAGGAAGCAGTGTAA

>PmsHsp14

ATGTCTCAGGCCGTATCGAATTTGAGCATTTTTCTTCCAATGTCATCTGGGAGGAGGACCAAAAATTGCCCATCCCCTGTTTTCTCAAAACCAGTTAAGAACAGCCTCAGGGCCATGGCAAGAGATGCAAGGGACAACCTTGACCACTTGCAGAGGGCCACCACCAAGCACCAACAACAACCACCACCCCAGCCCAAAAAGAGAGTCGCGCCGGCACCACCTGTAGGGTTGTGGGACCGGTTTCCGACGGCAAGGACAGTTCAGCAGATGATGGAGACCATGGAGAGGATGATGGACGACCCGTTGGCCTACTCGGGCGGGTCGGGTTGGGCATCCCCATTGCCGACGGAGACAGGCGGCTACAGCAGGGGAAGGACCCCCTGGGAGATCAAAGAAGGTGAGGCTGATTACAAGATGAGATTTGACATGCCTGGGATGACCAAAGAGGACGTCAAGGTGTGGGTTGAGGAGAAAATGCTGGTTGTGAAGGCAGAGAAGGTGACCAAGAAGAAGGAAAATGGGGTGCAAGAAGAAGAAGATAATGGTGATGATGAGTGGTCTGCTAAGAGCTATGGAAGGTACAGCAGTAGAATTGCTTTGCCTGAGAATATTCAGTTTGAGAAGATTAAGGCTGAGGTTAAAGATGGGGTCTTGTATATTACTATTCCTAAGGCTACCAGTAGTTCTAAGATTTTGGACATTCATGTAGGGTGA

>PmsHsp20

ATGTCTTCAGCTTTGGCTTTGTCATCTTCATCACCTTTGCTATCAACCAAAGCTAGGTCTTCAATCAAAACATATGTCACTGCGCCGTGCTCAGCCACCTTTCCTTCGCGGTTGCATAGGCTGCCCGTGGTGAGAGCTCAGGCTGGTGGAGATGGCAAGCTGGACGTGCAAGTCAATCAGGGCAACCAAGGAACTGAAGTCGAGAGGAGGCCAAGGAGGTTGGCTGGCGACATTTCACCTTTTGGTTTATTGGATCCCATCTCCCCAGTGAGGACCATGCGCCAAATGCTGGACACGGTGGACAGGCTCCTGGAGGACACTGTGACATTTCCAGGCAGAAACAGAGCATCAGGGGAAGTACGTGCACCTTGGGACATCAAAGATGATGAACATGAGATCAAAATGAGGTTTGACATGCCCGGCCTCTCCAAGGAGGATGTCAAGGTGGCTGTAGAAGATGATGTTCTTGTTATAAAGGGAGAGCACAAAAAGGAAGAGAGTGGTGATGATTCATGGTCGAGCAGGAGCTTTAGCTCCTATAATACCCGCCTTCAGCTTCCTGATAATTGTGAGAAGGACAACATAAAGGCAGAGCTTAAGAACGGTGTTCTTTACATATCCATTCCCAAAACTAAAGTTGAACGCAAGGTCATTGATGTTGCGATCCAGTGA

>PmsHsp21

ATGGCTTCTTCAGTTTCGATCCTTCTCAGAAGGGCCTCAGCCCCAACCCTCTTCTCCAAGCTCTCCAGCCCTATTCGCTCTGCTTCAGTTTCTCCCCTTGTCTCTCGCTCCTTCAACTCCAACGCCCAGGTCACAAGCTACGATCAAGATGATCGTAGTGTCCCTGTTGATCGCAGCACCACCGACAGGTCTCCCTCTCGCCGTCGTGACTTTGGCCCCACCTTCTTCTCAGATGTGTTTGATCCATTTTCACCAACAAGGAGTCTGAGCCAGGTTCTGAACATGATGGACCAGTTCATGGAGAACCCATTTCTTGCAGGGTCCAGAAGAGGCTGGGACGTGAAGGAAAACGGGGAAGCTCTGTTTCTGCGGATGGACATGCCAGGCTTAGACAAAGAGGATGTGAAGATCTCGGTGGAGCAGAACACGCTGGTTGTGAAAGGAGAAGATAAAGACTCGGAGGATGAAGAAGGTGGAGGCAGGAGGTTCTCTAGCAGATTGGATCTGCCTCCCAATCTTTACAAGCTCGATTCGATTAGGGCTGAGATGAAGAACGGGGTTCTGAAGCTGGCGATTCCTAAGGTGAAAGAGGACGAGAGGAAGGACGTCTTTGAGGTTAAGGTCGAGTGA

>PmsHsp23

ATGGCTTTGGCGCGTTTGGCTTTGAAAAACTTGCACCAGAGGGTGCTTTCTCCAGCTTCTTTTTCTGCTGCTTCTGTGCTGGGGCATGGTGTTAATGAGAGGACTGCTGGTGAGAGCAGAGGCAGAGGTGGCAATGAGATTGTGAAGAGATTTAGTACAGAAGCTAATGAGAAGGTGTCTGGTGAGAAATCAGAGAACAAAGATGTTGCTGTTTCTCAAGGTAAAAGGTCTAGGTTGTTCCCTAGAAGGCAACGTAGGAGGGGACTTTGGAGGGACAGTGACAGAAACTTCGTTCCTGCTCTTTATGAATTCTTTCCCTCGGGCCTTGGAAATGCACTGGTGCAAGCAACAGAGAACATAAACAGGCTGCTTGACAACCTCAACATATCACCATGGTCACTCACCGGGCGTGTCAAAGAGAAAAGTGACAGCTACAAACTGCAGTATGATGTGCCAGGGCTTGCGAAGGAGGATGTGAAGATCATTGTTCATGATGGGTTTTTGGAAATCAAAGGAGAGTACAAAGAAGAGGAGGAAGAAGGATCGGAGGGTTGGAGATATGGCTACTATGACACCACCCTCCAGCTGCCTGATGATGCCAAAGTTGATGATATAAAGGCAGAGCTGAAGGATGGGGTTCTGACCATCACCATTCCTAGAACTGAGAAGCCAAAGAAGGATGTGAAGGAGGTGAACGTACAATGA
