# Supplementary material for: Genome-wide identification and classification of the Hsf and sHsp gene families in Prunus mume, and transcriptional analysis under heat stress
Source: PeerJ. 2019 Jul 29;7:e7312. doi: 10.7717/peerj.7312 (PMC6673427; doi:10.7717/peerj.7312)
Supplement: Supplemental Information 8 [file peerj-07-7312-s008.docx]

>PmHsf8

ATGGAAGGAGTCGAAGACGCGGCGACAATGATCAACTTGGCCAACACTCCTCCTCCGCCCTTTCTGAACAAAACCTACGACATGGTGGACGACCCGTCGACCAACGCCGTCGTTTCCTGGAGCGACGGCAACAACAGCTTCGTCGTTTGGAACGTGCCCGAGTTCTCCAGGGACCTCCTGCCCAAGTATTTCAAGCACAACAACTTCTCTAGCTTCGTCAGGCAGCTGAATACTTATGTGGGTATTTCTTCCCTTTTCCCAATTTCGATTCTTCTGCTTTTTTCTTTGTTCTTTGTTTCTGCATTTTATTGTGGGTTTTTGGAGGTTTAGATAGAAACAAGAAGATGGTTTACGCGGTACTTCCGTTTAGAACTGTGAAATTTGGAGTAGTTACGCAAGACCAATGTTCAATTAGGTTTGATCATTGTTCTTTTGGTAAAGGTTTATAGAATTGGAGGTTGTAAATAGATAAACCATGAAGATAGATTCTGTTTGTGTGGATTTTATATCACAAGATTTTAGCCATTGTTGTTGAATTGGATTCGTGGTCATTCAGTGGGTTCCTTCAGGGCTATGTGGTCACTGTATCATGTAATCAAGTGGATAAATTTCAATTTGTTTTGTTTTGTTCGGAACTTGTAGAAAACCTAGCCCGAAAAAAAAAAGACAGTAAAAGATCAGAAGTACTTTGAGATTTATAATCGGAGCCTAAACCCTAATACTTATGTGCAAATTGTCTCATTTAACGTGCTTCTTTGACAAGTTTGTTCCTATGATTCATTCTTATCTTTACGGATTAATTATACAATTAATTTTTTTTTCTTCATAGTAAGACCGTAGGGTTGTTTTATTGTAAAGAAACCATTTTGTATAAAGTGGAACATAAAAGTTCCTTAGGCATTTGCTGATATCCCAGAAAATTTCAAATGATTGGCATGTTTAGCTTTCTTTAGCCTCTAAATTCCCATCTTATTCAGATGAAGACAGTTCTTTAAAACTTGGTGACACTGACAGGCAACTTGGTCTTATAAGCTCAGTAGTTTGATCTTTTTACATGTCAACGTAGTTAGTGATTGAAAGTGTGATGTGTTGAAAATGTTTTGCACTACAAGGGCGATGTAGTTATATGGCATAATCCAAAATGTAGTAATTCATGTTGTCTCAACTCTTACAGATGACAGGTATCAAGTAGTGATCTAGTTTTGGTAAATACTTATTGGCATTGTTAATAGAAACTAGCGGGATGTAAAATGAATGGGTATGTACACCTGAGGTTTGTTCCTTTCCCCTTCTCTTACTTCCAGATATTATAAGTTAGGAATACTTCTTTCTTCCTTTTCAATTCATACATTCCCACCAGGAGTTAGTGTTTTAGAAGATACATGAAATCAGTTTCTTTTTTTTTCTTTTTTAAGTTTTGTTTAGTAAGACAAGAAAGGTGAAGTTAAGCATTTTTTTTTTTTTGGCAGTTGCATTATCCATGTGTTTGGATATGACACTTTTGTTAGTTTTTGTCTTATTTGTTTAATATCATATTATTTGGAACTTTTAATAGGGTTATAAATTTAAACCATAGAAGGACGATCGCAAATGACTATACTTCTGCATATATGTATATAATATGCATACATACACAAACCTGGGTGTGTGTATCCGACATAATCTGGTGAGTGGTTCGAGCAGTTTCCTACGCACCAGTTAACAAGAAAAATCCATGTTCACCGACCAATAAATCAAAATCAAATAGCTAGCATGCATGTCAAAGAAAAGTGCTTTGTCATAATTTGAACATCAGTCGTTGGATTTTGATCCTATGACTGGTGAGCATGAATTCTTTGCTCACTGGTAATATGAGATCAAAATTGCATATATACGATATTTGTATTTATCTATATCTATATATGTATGTATTTTTGTACATGGATGTGTTTTTGTGTATATGGAATTACTCTAATAAGGTGTCTGTCCTTAAACGAGAACTTGTTTCTTGGTTGTTAAATCAGATTTGAACAGTTGAGATGTATCACCTCTTAGTTGTTTAGGGATCTGCCATTTTCAACCTCTAGTAAGACTGGCAAGCTGTAGTGGGTTAATATCGATAAATGGCTATTGTATCATTATTTTGTTGTTTCTAATGCGGACATCTTTTTCACTATGTTGTAATTTATTGTTATGATTTAATTATTGCAACAGTCCCTGAACTTATATCCAAGTTCCACTTTAGTCCCTCGATTCAGTTTTTAAATTTTGTGGTGCCTGGGTACAAGTTCAGTGACCATTGCAATAATATTGTTTATTCAGCAATAAAATTGTCTCCTAATATCTGAGAAACAAGCCTTTTATCATCAGCAATTGCAATTCGCAGAAAGTAGAAGTTTTACTTGTGTTGGTTATTGGAAATAAATAAAGAAATGATAAAGAGTGGAAAAGGAGGCAAAAGCTAATAAGTTCCTTTGCAGCATTTGGCTGTTGGAGGGTTGATACTGCAATTATGCCTATGAATGGTGCTTGTTATCTGAGGCTGGATTATGAGACGAGAGATTCTGGCTCTTTGTCCTATGCTCCAAAAAAAAAAAAGTTTAAGATGTATGCGTAACTTTTGACTTATATTTTCATTATATAATTTTAGTATTTATGGTGTTGCATTCAATTTGCAGGGTTTTAGAAAGGTTGATCCAGACCGATGGGAATTTGCAAATGAAGGGTTTCTAAGAGGCCAGAAACACCTTTTGAAGACTGTCAGCAGGCGGAAACCAGCTCATGCACAGAGTCATCAACAAGCACCACCTCAAGTGCAGAGCTCTCAGGTTGGGGCATGTGTTGAGGTGGGAAATCTTGGGCTGGAGGAAGAGGTCGAAAGACTTAAAAATGACAAGAACAGTCTTATGCAGGAACTTGTTAGATTGAGGCAGAAACAGCAAGCAACAGATAATCAGTTACATAATGTTGGACAACGTGTGCAGGGAATGGAGCAGCGGCAGCAGCAAATGATGTCATTTCTTGCAAAGGCTATGCATAGTCCTGGATTTTTATCCCAGCTTGTACAGCATCAAAATGAAAACAACAGGCGAATCACTGGAAGCAATAAGAAAAGGAGACTCCCAAGGCAAGAAGATGAAATTTTGGTTGGGAAGCTTAGCACCAAATCTCTCGATGGACAAATGGTGAAGTACCAACCTTCAATGAATGAAGCAGCAAAAGCAATGCTGCGGCAGATCTTGAAGATGAATACATCTCCTAGGCTGGAACCGTCAATCAATCCTGATGCTTTCCTGATTGACAATGTTCCTTCTTCTGATGCATTAGAAAGCGGTGACACCTCTAACCGGATTTTGGGCGTGACCTTTTCAGAGGTTCCACCAACTTCTGTGGAGTGTTATATGCCTGAAGAAGAGTCTGGATTTCCCGACAGTTGTCATTCTACAACCATTTCTGAGATCCAGTATTCTCCTTATGCGGTTACCAATTGTGTTAAAGCAGCTCAAGTTTTGGAAGAGAACATGCATAATTTTCAAGAAGATGCAGTTATGCCTGAGTCAACGCAAATGCAAGGTGGTGTTCCAGAAAGCACTGTAGAAATCCCCAACGCAAACTTCATGAGCTCTGAGACTGGGAATGCAGAGTACATGGATATGTCTGCGGTTTTGGATGGGACCCTGCCTACAGAAACTGATGCCTTTTCTCCTGAGCCTGATGTAGATGCTTTGCTGGGCTCTAACCTTCCGGGAATTACTGATATCTTCTGGGAACAATTTCTTCCAGCGAGCCCTCTGACTGGTGACGTAGATGAAATTAATTTGAGTTCCACTGATGGGGGGACCACGGATCAAGAGTTGAAGTTGGCGGAGGAGAATGGATGGGACAAAACTCAACATATGAATCATATTACTGAACAAATGGAGCTTCTTGCACCAGGGAGCAGAATTGGTTGA

>PmHsf12

ATGGGAGGTGCTAATAACAACGGCGACGATGCATCAATGGCCGGCGGCGGAGCCCAGCAGGCGGGTTTGGCTCCGGCGCCAGCACCGTTACTGAACTCGAACGCGCCGCCGCCTTTTCTGAGCAAGACGTACGACATGGTGGACGACCCGGCGACCGACCAGGTTGTGTCGTGGAGCCCCACCAACAACAGCTTCGTGGTTTGGAACCCGCCGGAGTTTGCTAGGGACCTTCTGCCCAAGTACTTCAAGCACAATAACTTCTCCAGCTTTGTGAGGCAGTTGAACACCTATGTATGGCACTCTCTCTCTCTCTCTCTCTCTCTCTCTCTCTCTGTGTGTGTCTTTTCTACTCATGGTTTATGCTTTTATTGGTGATTGATTTGTTTCAATGGTTGATGGAAACACAGATTGGTCTTATCTGGGGAGTGTGGTATATAGTTTACTGAAATGTAAATCGTTTAGATTGTGTGGTTAATGGGAATGCTCAAGATTGGAAAAAGAAAAAGATAGTCTGTCTGTTGCAATCTTGCGGTAGTAGTGTAGTTGAGTGGCAATTCTGAAGTGCTTTGCACTCACTTTGTCGATACTGATTGTTCGAGGGGTGAATGGCTTAAAATCTCCCATTCGGTCCCTCGGAAATTTGAGGGATACTTGAAACGTTAGGAACTCGCGTTTTAACAAAATACTTGTTTTTGGAGTTGAAAGCTTTTGGTTTTGGTTTTTTCTTTTGCTTTCTTTTTTGGAACTATACATATGTTAGAAGGGTGTAAAAAAGTTGTCCTTTCAAATGGAGGTAAGTCAGATTCAAAACACTTAAAGTTGTAAAGAGGTTTGAGAATGTTATGAAGGATTTTTATTCTTCGACTGTTTTGAATAGAGGTAGAAGGGCCACTTGATCAGGTGGTATCTTAGAGTAGGTAGAATGTTGTTTGGGGGTTGGTAATTTGGTCATGAAGGATGAAGACTAAATGGGAAGTCTGTTGGTAGAGATTTCCTTGAGAGGGAGATTCTTTACGGTATGCTGTTAGCATTTTATGGAGTGAAAAAAGGGCAGGAATTGTTAAACAAAGTGAACAATAATTATTTTTATGGTTGTTTCCTGATGTACAAAGCTTCTTGATGAGAACATCAAAATTTTGATTTGAGGAAAAGTGAGGGGAAAAAGAAACAGGGTTGAGAGTGGGATCTGGGGTTCAATACCAATTTTCTGTGCGTGTTTAATTCACCAAGCATAATAGAGTATCCCGTGCATGCTTAATTCACCAAGCATAATAGAGTAGCCTATGCAGCATGCTGATTCGTCCCGATGTCTGGATATGTCTTTAAGCACTCTTTTCCATGAGCTTCTCACTGCCTTACACAACATGTTCTATACCATAAACTCGAATTTTTAAGCGCAATGTTGCATAAAAGTTTGACTATGGACCTACAAATACCACTCAAAAGCTGCTTGAAACTTTTGATATGTGAGATAAGATATTTCAGGTTTCTTATTTACATTACAAATCTTGTTTGGGAGATGGTTGCTGTTATTTGGTTTTATGTTTTGATTGTGCTATATGGTTTTCTCCAGTAAAGTTGTGTGGGGTATGTGGGCTTGCATCAAAGACACGCTTAGAAAGAACTGGTAGAGAATGTCTTCATAGCTACTCATTGTTTATTTTGGCTGAGCCACATTTCGAATGATATTATGTAAAGGTACTAGTTATAAGTTATGTGAACTCTGCATATGAAATTGCTTGAATAGAGAATCAAAGATTCTAAACTTGAGTGTAGTGTGGTATCAATGTGAAGACTTTTTGGTGGTTATGGGGAGTGTACTTGTTGAGTGGTGTCACTAGTAACTTAGACTCTGGGAATAAGCACACTATTGGTTCCTGACATGTACAAGGTGCCCATGTGAAGTTATTTTTTAACTTTTTGGGAATACATTAATTGGCTTGAGATAAGTCTTGTGAACTCCATTGATCAACTTTGTAGTTGTTTTTCTTATAAATGGGTAGATAATCGATCCATTTTTCTTCATCAATGTTTTCCTGCCTGCTTGTCCTGATATTGTTTTTGTTTCTTTCTTTGTTTTGCTGAATCATGCATTTTTTCCTTTAATTTTCATTATGTTGATTGAACCCCTACTTCATTTTGGTTCAAACAGGGATTTAGGAAGGTTGACCCAGACCGCTGGGAATTTGCAAATGAGGGCTTTTTGAGGGGTCAGAAGCATCTCCTTAAGAGTATCAACCGGCGAAAACCTGCGCATGGACATAGTCATCAACAGCCACAGCCATCACAAGGACAGAATTCAGTGGCTGCATGTGTAGAGGTTGGGAAGTTTGGCCTTGAGGAAGAAGTTGAGAGGCTAAAAAGGGACAAGAATGTGCTTATGCAGGAACTTATCAAGTTGAGGCAGCAGCAACAGTCTACTGATAACCAGCTGCAAGCAATGGTGCAACGTCTTCAGGGTATGGAACAGCGGCAGCAACAGATGATGTCATTCCTTGCAAAGGCTGTGCAGAGCCCAAGTTTCTTGACTCAGTTTGTACAGCAGCAGAATGAGAGCAATAGGCGCATAATTGAAGTCAACAAAAAACGGAGGCTCAAGCAGGATGAGGGCGGGGACTCTGGTACTTCTGATGGACAGATTGTGAAGTATCAGCCTCCAGTGAATGAAGCTGCAAAGGCAATGCTCAGGCAGATTATGACAACAGATACTTCTTCTTCCCGGCTGGAATCTTTTAATGACACCCCTGATAACATCCTGACTGGGAATGGCTCATCATCATCATCCAGTTTAATAGACAGTGGGAGCTCTTCAAGCCGTGCGTCAGGAGTGACCCTTCAAGAAGTCCCGCTGACTTCAGGGCTTGGTTCGTCATCTGCAATTTCTGAAGTACAATCTTCTCTACAGGCTGCAAACTCTGGAACAGTTACGAGAGCTCCATTCTCAGATATAAATGCCCTCGTTGGAGCACAAGAGGCACAATCAATCCCTATTTCTCAGGCTGGTGTAATCATCCCCCAGCTTTCTCAAGTACCGGAAATGGTACCCGAATGTTTAGTTGATATTCCTGAAGAAAACATGGCACCTGATGCGGGTGTTGGATTTATTGAAAACATGGCATCTGACGCAGGTGATGGATTTATCGGCGACATATTGGGATTGGATGGGTCAATGACCATAGACATTGATAGTATTCCTCCTGATCCTGACATAGAAGCATTGCTAAAGAATTGGGATCAATTTTTACAAAGTCCAGAGCCAGATGAAATGGATTCTACTTCTGCAGGAGTGCCCATGGGAAATGAAGAGCAGCCGTCAACAGAGAATGGATGGGACAAAACCCAGCATAATATGGATAACCTTACTGAAAAAATGGAACGTCTTACTTCAGATACCAAAGGGGTTTGA

>PmHsf16

ATGGTGGTGCCTGAAGGTGGTGGTGGTTGTGGCGGTGGGGATGGTGGTGGTGGTGGTATATCTCGTCTGCCACTGTCATCAGCAACCCAGTCGCCAAAACCCAGCAATGGTTTAGAAGAAGCAGAGAACGAGTTGAATAAAGCCACAGAAGAAGTGGCTCCGGCCAAGGAAGAGCAAAAGGCAGTGACTTTCAAAGGTGGTAATTGTGATCGGTCATCTTCTTCGTCTTCATCCCCTGCTTTGCCAAAACCAGCCAAGGAAATGTTCTGTATTAAAGAAGAAAACATAGATGTTGTAGTTGTGGATGATGATGTTGATGTTGATGGGGGAGATGATGGGAACTTTAATGGTGGTGATGGTGGTACATTTTCTTCATCTTCATCCATGGCGTTGCCTAAACCCATGGACGGTTTGCACGAGGCAGGACCACCACCCTTCTTGAACAAGACTTTTCAAGCGGTGGATGACCCAGAAACCAATTCGGTTGTTTCATGGAGTGCATCTGGTCAGAGCTTCATTGTTTGGGACTCTTACGAGTTCTCCAGGACTCTCTTGCCCAAATACTTCAAGCACAACAATTTCTCAAGCTTCATCCGCCAACTCAACACTTATGTAAAAATTATATCTTTTCCTTGAAATCTTTCATCTCTCACATTTGATTTGTGTGTTGCATGTCCTTTTTTACTTCTTCTTTAATTTGTTTGAATGATTTATAGTTTTTTCTCATTTGAACATATGGTTTCTGCTTAGTTTGTTATGCATAAATCTTCCTGGTCATTGAGTTCTTGATGGATCTTATTATGCATTCAGGGCTTCAAGAAGGTTGATCCGGACAGGTGGGAGTTTGCAAATGGAGGGTTTCAGGGAGGGAAGAAGCACTTGCTGAAGAACATCAAGAGAAGAATCAGATATAACAAGCAGCCAACTGTAGGCTGTGTTGATTCAACCAAAACTGGGTTAGAAGCCGAAATTGAAAGTCTAAAGAAAGACCAAGACTTTTTGAAATTGGAAATCATGAATCTCAGACAACAACAAAAGTACTCACAGCATCAACTGACTGCTATTGAACAACGAATTCGAAATTCAGAGTGTAAGAACCAAAGGATGCTCTTTTTCCTCACCAAAACAGCCACAAATTCCACCATTGTCCAGCAGCTAATGCAGAAGAGAGTGATAAAGAGAGAGCTGGATGGGAGTGATCTACGCAAGAGAAGGAGAATGCCTTCAGTCCAAGTCCTTGAAAGCTTGCGTGATGGGATTGATACCAGCCTCAGTGTGGATTGTGGAACCCAACTTGAGGAAGAACTGGTGCCTATGCAGTCTCTGCTTGCTGAACAAGTGGCAGAGGCAAAGGTTGCCAAACAAAATGAAGGCCCATTGCCAGCTCCCATGATTGATAAATCAGGCAATGCAGTTCAAGATCTTAAGCCCCATGTGATGGCCAGAACCGGCACGGAAGACATGCCCACTGCTTATCATGGCATGTCTGAGAATTTCCTGGAGGAAAATGTAGTCTTTGATGATGATGAATTTGAAGTCGGTTACTCTAATTTTTATCAAGAATTGGAGGATTTGATTGGCAAGCCACATGATTGGAGTGGTTATGTAAGTCACTGCCTGATGGAGCAAGCTGGGGTAATTGGGGCCATGCCTTGA

>PmHsf2

ATGGATGGAGTGGTGGTGAAAGAGGAAGAGATTGTGACATGCACTGTTGGTTCATCATCCTCTTCTTCTTCAAGCTTCTCACCTCAGCCAATAGAGGGCTTACACGAAGTGGGCCCTCCCCCTTTTCTCACAAAGACCTTTGAAATGGTGGAGGATCCTTCTACAGACGCCATTGTCTCCTGGAGCAGAGCTCGCAACAGTTTCGTTGTTTGGGACTCTCATAAGTTCTCCACCACTCTTCTTCCTCGCTACTTCAAGCACGGAAACTTCTCCAGCTTCATTCGTCAGCTTAATACATATGTAAGTGATTCTCAAAAAACATGAACTTTATTGCATCGTCTTCATATTGAATGCAGAATTCTACATGGGTTCTTTTCTTTTTTGCAGTTCCCTTTTAGTTTCTGGGCTGACATGAAAAAATTGAGAAATCAACTAATAATAAAGTTGGTTGCTTGCTAATTACTGTAACATTTTTGGTTCAGTGTAAGAATTTCAATTATTGATTGTCATGCTTTTTTTATTCTTTCTGCAACTCTGTTAACTAGTTTCGATGCTCTCTTTGAATTTCTTATAAAAGTAATTCCCTTTCTTCTGATGGTCATGAATCATGATTTGATTTCATTGCTCCTCAGTCGGGTTTGCTAATTACATACTTGTTTAGTAAATTTCAATGCTTGCTCATCAACAGATTGCATGCAGGGTTTTAGAAAGGTTGATCCTGACCGATGGGAATTTGCAAACGAAGGGTTTCTGGGAGGGCAGAGGCATTTACTGAAGACCATCAAGAGGAGGAGGCATGTGTCACAGAGTATGCAACAAGAAGGTGGAGGAGGAGCTTGTGTTGAACTGGGCCAGTATGGACTGGAGACTGAGCTTGAAAGATTGAAGAGAGACCGAAATGTTTTAATGACTGAAATAATGAGGCTGAGGCAGCAACAACAAAATTCAAAGGAACAAGTCATGGCAATGGAGGGTCGGTTGCAGACCACAGAGAAAAAACAACAGCAGATTATGGCTTTCCTTGCCAAAGCACTCAATAGCCCATCTTTTATCCAAAACCTTGTTGAGAAGAAGGCTCGGAATAAAGAGTTGCGTGGTATGGAAATTGGTCGAAAGCGGAGACTAGCAGCTAGCCCCAGTGTGGAGAATCTGCAAGAAAAACCTAAAACCCATGTTGTGGACTACTCAGCAAGCCAAGATCAGGGAGAGTTGGAAACTATGGAATCGCAGATTGAGACCTTTTTCTCAGCTGCTGCATTGGATAATGAATCAAGCAGTGATATCGTAGACCCTCATTCAAGTTCAGTTGGTGGCAACTTAGGCATTGTTAATGAGACTACATGGGAGGAGCTGTGGAGCGATGAGCTCATTGGTGGCAATCCGGAGGAAGACGTTATTGTGGTGGGTGATGAATCAGATATTGATGTCGCGGTGGAGGATTTGGTTGCAGAGCCAGCAGATTGGGGTGAGGACTTGCAAGAACTTGTTGATCAAATGGGTTATCTAATCAGGTCCAAGCCTTGATGATAAGAAAATGTCTGTGATGGTGAAGTGGTTGTGTGCTGAGTTGGTCCCTGCAACCCCATTCCTATCTTTAATCCAGATATTGGTATATGATTTAAGAACTTCGGTTCCATTAATTATTTGTATGTGTGTGTTATTTGTTTTGTGAGTGAAAATAATATATGATTTGTGATGCAGCATGGGGGATGGTCAAAGTTGCAAAGGGGCCAACGATGTAGTAGCTGTTTTTTTCAGTATCCTTTAATAATTATTCATATCTAGTGTGTTAAATATTTTATTGGCACTTTCAAAGTTGTTTCAATGGTTTTCATTTGCTGTTTCTTCTTTGATGCTTTCCAGGCGGACCGGCCAACGGAGACCTTTGA

>PmHsf14

ATGAACCCAAAAGACGAGAGCTACCCAAAGTCTCCACCTACTTCAGCCGAATTGAACCCGGAGAATCCGTTTCGACCCGAAATGTCAGAACCGTTGTTGGGTTCTCAGTCAATTCCTTCGTTTACTTCTCCTCTGATGGAATTTGAAGCCTTTTGTGCTCTAAACCCATCGGAGTCTTCTTGGTCCTCTGGTGCTTTCGAGTTTGATGAGAAAGCTCCAACTGCAACGTCTTCGTTTATGGACGCTGGTGGCGCGGAGCACGTGGCCGTCCCGCAGCCTCTTGAATGTCTACAGGACAGTCCGGTTCCGCCGTTTCTGTCCAAGACTTTCGATCTGGTCGACGACCCGTCGCTTGATTCGATCATATCGTGGGGTTCCGGCGGCAACAGCTTCGTGGTGTGGGACCCATTGGAGTTTTCCAGGCTCATCTTGCCGAGGAATTTCAAGCACAACAACTTCTCAAGTTTTGTCCGGCAACTTAATACTTATGTGGGTATTTGCAGCCAGTATTGGCTGCCGCTATGATCTGTATGTAATAAATGTTGTTGTTTTGTTGTTCTCTTTTATGTTTTTTTATTTGGTTTTGTTTCTTTTTTTATGATGATGCTGTTTATCGTCTAAATTGGGTTTGATGAGTCTAATTCTGTAGAGATGTAGTCATGTATCTGAGGCCATTAGTATTGTATCTTTTAAACTCACCATGTAAGACTTTGAAGGAGGTGATGGCTTTGTAGCCTTAACTCCCAATTTTTTGTATTGAAGAGATTCATATGTAATTGACCAAAACTAAATAAAAGAGGGTTCATTTTTATGTAAACAGTTTCTTGTCGTGAAATGCTTGGCAATTTGTTTCTCTTTTTCATTAATGGTTTACACAGGTGAAGTGATAGACATTTGTTCTTGATGATGATATGTATTATGGTTTCTGTGGTTCTAGAAGATGTATGTGTTGAATTTATCAGGATAATATATAACAGTTTTGTAAGACTTGAATGTATCATGAAACTTAGTATAGAGAATCTAATTGGAATCGTAAAGGTTCTTTCATTGACTTCGTAGTTAGGACTGGAGGCTTACTTGTACGAAAAGAACATCCTCGTCTTTTGCAGGTAGAATTCAGGTTCCTATTGTTCAGATCTCTCTATATTATCATCTCAACTGCTCCCCTCCTCCCAACCACCACTTGTTAATGCTTCAAGAAGCTTTTCTCTATTTGTATTTGGTGATTTGCATATTGCACAGTGAGGTTCAGCTCCTCATATGAATGAAAGTTGATGGATTCAAATTGTCCTGTGTGATCATTTGTGTACTACTTGAATAATTATATCTATCTTTTGATTTTTGTACTGTTTCGTTTCCATTCATTATTGAAATTCTTTGGGTACTGTGTTCAGGGGTTTCGCAAGGTTGATACAGATAAGTGGGAGTTTGGGAATGAAGCTTTCAAAAGGGGCAAAAGACATTTGTTGAAGAAGATCCAGAGGCGCAAGTCACCTCAATCACTGCAGGTTGGGCCTTCTGCCGAAGCAGGGAGGCCCAGACTGGAAGGTGACATTGAGACTTTGAGGAAAGAGAGAAGTATGTTAATGCAGGAAGTTGGTGACCTGCAGCAGGAACAGCTGGGTACAGTTCACCATATGAAAGTAGTGAAAGAGAGGCTTCAGTCTGCGGAGCAGAGACAGAAGCAGATGGTTTCTTTCTTGTCCAAGTTGCTTCAAAACCCAGCATTCTTAGCCCGCCTTCAACAGAAGACAGGACAGAAAGGTATAGACTCTCCAAGGATGAAGAGGAAATTTGTTAAGCAGCATCAGCATGAACTAGGTAAATCAGATTCTTGTATGCAAGGGCAGATTGTGAAGTACCAACCTGCTTGGAGAAATCTCTCTGTAGTCCCAGAGGTGAATCCAGTAGTTCCTATTGAACAATCTCCTGATAATCTTTCACAAGTTATGGCAGGAAAACTGGGTTTGGTTGCAGAAAGCAAGCCATATCAATTTGCGGATGTAGCATCAGATGAGTTAAATTTATCAGCTGAACCAGCAGTAATGCGAGGGGTTATCAAAACACCAGAGGAAGAAGGTGAAGGGGCGTCAAGCATGGGAGCTGAAGATCCATTTCAAAAAGGGAAGAGTGTTCTGAGCCCAGAACAGGAGGTTAATCCCGAGTATCATGTCTGTTTCCAGGAGGATTTTGGGAAGAATAAGATGTTTCCGGAACTTTTCTCTCCAGGGATCGATAGCATGATTAAACAAGAAGATATATGGAGCATGGGTTTTGATGTCAGTGCTGGTATGTCAAGTTCTAGCAATGAGTTATGGAGTAACCTGGTCAACTATGATGTGCCGGAGATGGGAGTGACAAGCGGATTGTTAGATATCTGGGATATAGGTCCCCTGCATGCAGCAGGAGGATCAGGCATTGATAAGTGGCCAGCTGATGAATCTGCATTTGATGAGCCTGATAGTCAAGCTGGCCAGCTGAAAGTCGATACATCTAAACGTATTGATCCATAG

>PmHsf7

ATGGATGAAGTTCAAGGTGGCGCCAGCTCGTTGCCGCCTTTCCTTTCCAAGACCTATGATATGGTGGATGATGCTTCAACCGATTCAATTGTGTCTTGGAGTGCCAGTAATAAGAGCTTTATTGTTTGGAACCCGCCGGAGTTTGCCAGAGATTTGCTGCCAAAGTTCTTCAAGCACAACAACTTCTCTAGCTTCATCAGGCAGCTCAATACATACGTAAGTCAATCAAGTTTCTACTTTCATTCAGCTTCAGTTTTTTTTACATTGGTATTTATCAATCTTTATAATTTTTTAGTTCCTGAATTTGATTTAAATGCTTACAATAGGGTTTTAGAAAAATTGATCCGGAACAATGGGAATTTGCCAATGACGATTTTATCAGAGGTCAGCCAAACCTTATGAAGAATATCCATAGACGAAAGCCGGTTCATAGTCACTCTTTGCAGAATCTCCAAGTCCAAGGGAATGGGACTTCCTTATCCGAATCTGAAAGGCAGAGTATGAAGGATGAAATCGAGAGGCTTAAACATGAGAAAGAACGCCTTGGTGTGGAGTTACAGCGGCTTGAGCAGGAGCGGCAGGGATTGGAGTTGCAAATGCAGTTCTTGAAGGAACGTTTGCAACACATGGAAAGGCAGCAGCAAACCATGACCAGTTTCGTGGCCCGGGTTTTGCAGAAACCGGGGATTGCCTCCAATCCTGTTCCGCAATTGGAAATCCATGGGCGAAAGAGAAGGTTGCCAAGAATCGGTTGGTCTTATGATGAAGCGAGCAATGGAAATAATCAGGTGGCAAGTTCACAAGCTGGTATTAGAGAAAATGCGGATATGGAAAAGTTGGAGCAGTTGGAGTCATTCCTAACATTTTGGGAAGATACCATTTTTGATGTTGGAGAGGCTCATATTCAGGTTGTTTCAAACGTGGAGTTGGATGAGTCTACAAGTTGTGTCGAAAGCGCAGCTATATCTTCAATACAACTTAATGTTGACGCTCAGCCTAAATCCCCGGGAATTGACATGAATTCTGAACCTGCTGTAGTCGTTGCTCCTGAGCCTGCTGCAGCTGTACCTCCTGAGCCTGCTTCCTCAAAACAACAATCCTCAAAAGAACAAACATCTGGAATCACTGCTTCCACGCCAACTGGCGTGAATGACGTATTCTGGGAACACTTCTTGACAGAGAATCCTGGTTCAGTGGAAGCTCAGGAAGTTCAGTTGGAAAAAAGGGATTCTGATGGTAGAAAGAACGAAAGCAAACCTGGTGATCATGGCAAGTTGTGGTGGAATATGAGGAATGTAAATAACCTTACAGAACAAATGGGGCATCTTACTCCAGTAGAGAAAACGTGA

>PmHsf9

ATGGAGGGGACTCAAGGTGGTTCTAATGCACCAGCGCCTTTTCTTACAAAAACATATGACTTGGTGGATGACCCCTCATCCAACCGCGTTGTGTCTTGGAGTGAAACTGGTTGTAGTTTCGTGGTTTGGGATCCCACAGAGTTCGCCAAAGAAATGCTTCCCATGTATTTCAAACACAACAACTTTTCAAGCTTCGTGAGGCAGCTGAATACATATGTGAGTTCTTTAGTGTTTCTTTACTTTTGGAGCTTATTGATTTATTTTCAATTTTCTGTCTTCGTGTCTTGTTTTTGTTTTTATTTCCTTGGGAAATTATCCATTTAGATATAGCATGTCATTTTCTTTTGTGCTTGATTTTTGGTGTCATGGGTTAAACTCAAACAAATGATAGACTAGTCGCATTACAAAATTCAGAAATGATTAGTATTTCTGCAGATTATATGTTTTGTTTGAACTTTCTTGTAAAGTTTTGTGCTTGTACACGCTTTATTGGCTTCCAAGTTCCAACTAGGAATGTTTATTTTGCTTCGTAAATCTATCTGTTTATCTGGTCTGTTGGAGCATTTCTGACTTAACTCCGTTAGCAGTTGCAAGCATGCTAGTGTTGTTTTGGTTTGTTTATGACCAAAATTTCTGGATGCTTGTAGTTGTTTGTCTGTCAGAGCTAATCTTGATTAGCAGGTGGTCCTTAGAACTTGGTGGAGTTCATAGAATCAAAATGGAAAAGCGTTTAGTTTATGAGAAGACTGGAATATGGCCGCCACCATTAGCCCCTCTAGCTCTGAGCCACAAAAACTTTTTCAATATATGCGAAACAGGCAAAAAATTCTTAGATTGGGAACAAGAGTAAATGGGGTCTGAACTGTGAAGCTGACAAATGGACAAACCTTGTCTTGGAGAAGGGCTCTTTGAAGATGATGGAGAATTCCATAAACACTTCATTTGATTGGCAATCGCAAAGCAGTGGTTTTTTATTCGGTACCATTTGGATCAACTGTGATAGTTCATGGCTCTTATTATTGAGGATGCTGTTCAGCCTGAAAGGACATGATTTGAAACTTGATCAATGAGGTTATTTAAAAGGCTTGTATAGCAGTGTTTATTTAGCTTGAAGTGCTGAGAGACATTGAGTAGGTTGGTTTGCAGTTGGCATACATGGTTGGGCCTCACTGGAAAGGTTTTCAAGACATGCTACCATGTTGGGGCTGGATTTCTGAGGTAGATTATAGATTAGGTATCTTTGTGGTTTGTAGGTCCTTGTGTTTCCTATGCTGTGTTACTATTAAAAATTCAGGGGCACTGTCTGATTTTAGAGTCGTATGGTCACCGCTGCTAAGAGGAGTTCAATAATTGGGGTTATTGCCCTGGTTTGCTGAGATTTGTTGGAGGGATTTTCAGGGTGATGGAGAAAGTATACTTAAGCTATAGAAAGATTATACATTCTCTCCCATCCAAAAAGGGAGGTGGGCTGAAGTTGAAGAGTTTGTTGAAATTAGTTTTCCTATACTACCTGAATTTTTCCCTTCAATTCCCAAAACCTGAATTAGATTCTGTCTAAACTTACCTTTATTTTCTATTTAATTAAAGGAAAAAGGGTTTTAGTTGCAATTGATTAGGCTAATGCTACTGTATGGTTGAGAGATTAGAGAGCTTATCCAGGGAGAATTTAAACAATTACTCAAGCGATTTTCGAAGCTTAAAATAGTTTATAATGGTCTTAACCTGTGTGCTTGATTTTATATAAAAGCCAGCCATCTAGTATTGTCTAATCTAGCACCATCGGTTCAGTTTTTTTGTTTTTCTATGTTGCACCTGCATTTTGTTTTTCGGTTATTAATGTGTGTATGGTTCTTAATTGTTGAAAGTTTGTGTTCTGTATGAGCAGGGGTTTAGGAAGATTGATCCTGAACAGTGGGAGTTTGCAAATGAGGAATTCTTAAGAGGAGGAAGACATCTGCTGAAGAAAATTCACCGCCGCAAGCCAATTCATAGTCATTCCATGCAGAATCATGAGTATTCTTCAGTTCCTTTAAGTGAGACAGAAAGGGAAGAATATGAGAAGAAAATCAATAGACTGAATCATGATAAAAGCTTGCTTGAGTTAGAGCTACAAAGGCATCAAAGAGAGAATCAAGAGTTTGACTATCAAATACAGATATTACAAGAACAACTGCAGAAAATGGAACATCAGCAGAAACAGTATACATCCGTCTTGGCTCAACTACTGCAGAAACCAGGATTAGCTTCTATACTTATGCAAAAATCAGAAATCCATAACAAAAAGAGAAGATTGTTGACATCTAGCCACGTTACTGATGATTTCAAGATGGAAATTTTGAATTTTAATACGCAGAACGAAAACCTGGGTACAATTTCAACTCCCATAATAAAGTTGGACCAGCTTGAGAAGATGGAGTCTTCCTTAAACTTTTGGGAAGATTTTCTACATGGGATCGGGGAAGCAATGCCTGAAGAAGTAAATGATATTGGCTTGTTATCTCAGGCCTCTCCCATCATTGTTACAGAAATACAAGACCCTGGCATGAATAGCAGGCCTTGCTCACCTAGATCGCGTTTATCTTCGCCAAATTCAATGAATGTTTCATCCCCAGAGGTGGTTGGGTCTGCAAATTTTCTTGACATCCTTGCTATAACATCAACATGCCACAATGTTGATTTCAGGCCAAAATCTTCAGGGATTGATATGAACTCTAAGCCCGATACTGCTTCTGCGGCTGAGGCCTTGAAAGAAATGGTACAGGAAATGAAAAATGCCGAGCCTGCTGCGGCAAATGATATGTTTTGGGAACAGTGCCTGACAGAGACCCCTGGTTTAGATGATGCGCAGGAAGTACAGTCAGAAAGAGGGGATAGCGATGGCGGAGTGAGCAATGCCAATCCAGCAATTCAGAAAAAGCTTTGGTGGAACACAGATGTAGATAACTTCACAAACCAAATAGGGCGCCTCACTTCAGCTAGTTGA

>PmHsf4

ATGGAAGGAGCTTCGACGGCGGGCGGAGGAGGCGGTCCGGCGCCGTTTTTGCTGAAGACGTACGACATGGTGGATGATTCAGCGACAGACGAGATCGTGTCGTGGAGCACGAACAAGAAGAGTTTCATCGTTTGGAACCCGCCGGAGTTCGCTCGGCTTCTGCTCCCCACTTATTTCAAGCACAACAACTTCTCTAGCTTCATTCGTCAGCTCAATACATACGTAAGTTTCTTCCCTTTTAAGATTTGATTTAGGTGAATTGTTAAATTCTTGAAAAATTGAAAGCATCGAGCATTTTCCTCCCAATTTCCTTTGTTTGGATGTAGCCTGCAAAATTTTCGTGTTTTACTAATTTTGTGTTTGGGTGATTGTAAATTACCGGTTCAAAAATTGGTATACATAGTATTTAACAAAATCCATACAGATGACTTACAGCAACCGCAAATTTTGAATTTTAGTAGTTCACCGAAAAAATTAATAATTTTTAATATTAGTTGAGTATTGCTATTGAATATCAACTTAGAAGACTCCGTAGTTAAGCTGAATCATTGTTTGGTTTCTTTCTTTTTTTTCGGTTTCTAAGTACTAGCATTTTTAAGAAATTCCAGGAGCTCGGTAGTTCTGAGTTTAGGGAGATCATGTTTTTTTCCCTGTTTTTGCAGATTGTCAAGCAGTTTTTTTTAGCTTGTATTTAATTGTTTTTTAACCCTTCCTGTATGGTGTTTATGGTTTTGAGCTGAATTTTTTTATAGTGAATATGTTGCCAATTTTGAATGTAATTTGTATTTTCTTATTGGTTCCAATAAAAATTTGTTTTTCATTAATATGGAAGAAAAAAGAGGGAGCTTTTAGGTGGGGCTCATTCAAAGTGTTCAATGAATTTCTTAGTCTGGCTCAACCAAACTGTACAGAAAGTTCTTGGTTTTCTTTTTCTTTTTCCAGTTTGAGAAAAAAGATATTGCTTTCAACCATAAAACCTTTACCAAATCTTTAGCGATACATATGAAATTTGGATTATTGAAGGGGTTTTGGCTGCCTGTTGAGGTTTTCAAGCAAGAAGAGCTTATGTGAAGGCCATGCTGTGGCTAAGGTGTCATGTCCCAAATGTGCCGCATTGAAAAGTGGAACATTTTGGGACCACATATGAGCTCTTGGGCGGCCTTCCCTTGCAGTTTGATTTTGTAGGTTTTAGGGCATGATATTTGGTATCAGAGCACTTTCACCATGCAACTAGGTTAGAGTCCAGGAAAGGGTCGACCTGTAGGCTAGATTATGATGCATCATTATGTATGGTCATGATGGTAAGCCATTGGATACTGGAGGAAGGGCTACTGTGGAGCTATCAGGAATGTGTCAACAAAATGTGCAGACGGGCTTGGGGTACTCTGTTGTTTCATATCCTAGATATCCAACATTGAAAGATGAGACAAGACTTCAGGTAATGTATCTTCTAAAATGCTGGTATTGCAAGGGAAAACTATATGGGCCATGACACTAGGATGGGAGCTGAAATTTTTTGAAGCAATTGTTTTTTGCTGCTAGGGAAGTATTTTAACTGGAATAAGAGGCAAATTTGACTTAACTGACCTTGATTAAAGGCAAGGAGTTTTGAAAGATGAGTGTCTTGATGCCTTGAAAAATGCCCTAATTTAAGTAAGAGTCAATTAAAGTAAATAAAAAATTAGCATCCTCACAAAGAGATTTCACTGTTTTTAGTGATTAATTTGTGTTTGTTTCGATATGTATAGAAAGTCTTGCTCATCAAACATTTGTGTTATGAAGCTTTCTAAGTATTTTTTCGACATGCATTGCTTGTGAAATGTGCCAGGCCATCTTTAGTCTGAAAACAAAATGCAGTGCACCGTTTTTTCATATCCAAGTGTTTCCATATAAAACATATGAACCACCTTGCTTTAGGTTGATTGCTACATTGTGGCAAAAGTTTCAATACATATGTGTGTGTATATATATATATATATTTTCTCTCATCCGGATGTCCACACTTTATTACTGCGCGGATTCCATAGTAAATAGGGAGGAAAACAGGGAGGGGTAGTAGAAAATACATGGCGTCTACACAGTAATAACGTCCGGACATCTGCATAGGAGAATTTCTGTATATATATATATATATTTATTTATTTATTTATTTATTTATTTGTAAAATAAAATGAATGGTATTTTCCTACATGTAAGATTACTAATGCTCAATTTGTATATAGTTAGTTGACCAGTTTTCTTACAGTTGAATACCTAACCAATGAGCTTGAACAATATTTTGAAGTTTCAATTGCTTCTAATGTGTCCACTTGACCATTAAGAATTATTTGACTATGAAACTTGAAAGTAGTATGATCCTCCTGTTTTATCCACTACTAGCACTACCTTTAATTTGTTTGTCAATCCTATTTACCATCAACTCCATGGTACTTTGGACATTTAGGAACATATTATAGTCTCTTCGTAGTCAGCATTAATTAATACCAAATCACCGAAAGTGGAATTTTGAGCAGAAATCGTCTTTTAAAAACTTAAAAGCAAGTACTATCTAATTGATATAACTGTTGCTAAATATTACTTTGATGATTTTGTTTTCAGTCTCTTTACCTTCTTAGGGCCATCCATAGTTGCGTTTGGGTTGTGCACCAAGTTGAAGGCAGAACGTTGATATTTTTCTTTCAAATGATTAGATTTTGCAATTTTAGGATTTTTAACGTTTAGGCCTGGAATGTGACAATGGATGACGAGTGTTATGGTTTTGATGGTTATTTCCCTAGGAGTCTGAATCAAAACAGTTTTTAGAATTGAGTCTTGTGTGTGTTATAAGATTTTATGTGCAGGGACAGATCTAAAGTTTCACAACTTTTTTTGCTTAGAGCATATTCAGTGTTTTTCTATAATTTCATGTTATGTCATATTTAACATCAAACAGCAAAAAAATGTGCTCCAACATAATGCCAAATGGCACCCTTTTACAATTTCTCAAATAAAGTATATTTTTTTTTCTTTTTTTCATTTTCTGTGCAGTTGCTACATTGCATTGTATTTATTGAAGTCCTCTGATTTCTTTGTTTTTATAATGTGGATTTGCATGTATTAACGAAAGAGATGTATTTTTCATGGAGGTAATATATTTAATTTAACTATAGGGCCTTCCTCGGTTGTGTTTTCCAATGCATGAATTTTCTTGATATGTTCCATGGCTTATGGTGAGCAACTCATAATTGAGGAATTCATTTCTTGTTCATTGAGTTTTATCTCTCGCATGAATCATACTTTCAATATTCCCCCCGCTCACCCACTTTCAAATACATTGTGCATAATTTTGTATCATCTCTAATTGATCGCTGTTTTTACATGGCGGAACTGAGTCTCAATATAATGCAACTAAATACGTTGGGACATAAATTTGTCACAGGGATTTCGAAAGATTGATCCAGAGAGATGGGAATTTGCTAATGAAGACTTCATACAAGATCAAAAGCATCTTCTTAAGAATATCCACCGCAGAAAACCCATCCACAGCCACAGTAATCCTCAAGGTTCTATGGTAGATCCGGAGAGAGCGGCTCTTGATGACGAAATAGAGAAGCTTTCGCATGATAAAGCCACACTAGAGGCAAATATTTCAAGGTTCAAGCAGCAGCGATCTGATGCAAAGCTGCAGTTGGAAGACCTAACACAGCGGGTGAATTCCATGGAACAACGGCAGAAGGATTTGCTGAAATTCTTAGATAAGAATGTTCAGAACCCTACTTTTGTTGAACATCTCACTAGAAAAATCGAAGCTATGGATTTCTCAGCATGTAATAAGAAAAGGCGATTGCCTGATGTTGATCACCTACAGCCAGTTGTGGAGAATAGTTTTGTGGATAACCAAAGTAGTTCCAGATCTGAGTTTGGGAATATTTTCCACCAAGACTTTTCAAGTAAACTGAGACTAGAATTATCACCAGCTGTTTCAGACATTAACCTGGTTTCGCGCAGCACACAGAGTTCTAATGAAGATGGGTATAGTCCAACTAGGAAAATATCTGAAGAACTTAAAGGTGTACAGAAAAGAACAGAAGGCCTTTTATTTGCACCTGAAACCTTAGAACTTTCAGATACTGGAACATCTTTTGCATTCAAAATGGATTCGTTGTTATCACGAAAAGCACTAACTGTTGGGAACCCGAGACTTCATTCGCTGCAGCCAGGTTTGTCTTCTAACGAAGAAGGTGATGGCCAGATATCCTGCCAATTAAAGCTCACTCTAGCATCTTCCCCGTTGCAAGTCAATAGTAGTCCTCATTCTGCTACAATACCCCAAGTAGGTCAGGATATCAGCAAATCCCTAGCATCAGGATTAAATGCCATTGGCAAGGAATCAGATATAAGAGCCTTTACAAACAAAAATCCAGCTGATGAAGACATGCATAAAACTTGCTCCCAAGAAGCCACAAATAACAATCAAGGGCCTCCACCTGCTCCAGTTAGAGTAAATGATGTTTTCTGGGAACAGTTCCTAACTGAAAGACCTGGCTGTTCAGAAAATGAAGAAGCAAGTTCTAATTATAGGGGAAATCCATATGATGAGCAGGATGATGGAAGGCTAGGCCACGGAATGTCCAGAAGTGCCAAGGATGCTGAAACACTCACTCTTTGA

>PmHsf5

ATGAATTCTGATCAAGACCCAGTAAGTTTGTCGTCTTCAGGTGCGGCACCTGATCATCCTCTGCCAATGGAGAAACTTTATGACCAAGGCCCCCCACCATTTCTCACCAAAACTTATGACATCGTTGATGACCCAACAACCAATGATATAGTTTCTTGGAGCAGAGACAACAATAGCTTTGTTGTGTTGGATCCCCAGAAATTTTCCATGAGACTTCTCCCCAGATACTTCAAGCACAACAATTTCTCTAGCTTCGTCAGGCAGCTCAACACCTATGTAAGCATGACTTTCTAGGCTGTCCTAATTGCTCATTTATATGATTTTTTTTCAAAGAAATTGTCATGTTGTTCTTTTCAATTTGGATATTGTATTCAAATCTATGATATATCTGTGGGGTTCTTTTGGGTTTGACTACTTTGTACATTCATGCTCATGAAACAGATAGAAATGTGTCATTTTTTTAATCTTTTCTGTCTTTGTTTAATCAGGTCCTTTTTTGTGTATTTGTTTAAGGGGTTACTTAATGTGGACATATTGTTTGTTAAATGGCTTCAAAAGGAAAAGTGCAGAAACCAATGTATAAAATATGCATGATACTATTGCAGAGATAGGGTCAGTTTTTGTTTGGTTTTTGTTCTTGTTTTTCGTTCTGTTGGATTTGTCAACTTCAAAGCTTCTTAGGGGTGATCTGATGCAAACTAAAAAAATTAGTAGACGCAGCCTTTTTATGGATTTGATCAAGAAGAAGTTATTTAATTAATGTAGGGATTTAGAAAGGTTGATACAGATAGATGGGAGTTCGCTAATCAAGGGTTTCTCAGAGGGCAAAAACATCTTTTGAAGAATATCCGGAGGAGGAAGACATCTTACCATCCTCAGGCTTCACAGAAAGCTTTGGACTCTTGTGTTGAAGTTGGAAAGTTTGGATTGGATGGAGAGATTGACCAGCTGAGGCGTGATAAACAGGTTTTAATGGGGGAACTAGTGAAGCTTAGACAACAGCAGCAAACTACTAGAGTTTACCTCCACGGAATGGAAAATAGACTGAAGAGGACAGAGATGAAACAGCAACATCTGAGGAATTTCTTGGCAAGAGCAATGCAGAATCCCAACTTTGTACAACAATTGGCACAACAGAAGGACAAAAGGAATGAACTCGAGGAAGCAATTAGTAAGAAGAGAAGGCGGCCTATTGAGCAAGGGCCTAGTAGTTTTGAGGTGGATGAATTAGGCCAAGTTGGAGTAGAAACTTTTGTTAAAGTTGAACCTCAGGAATACGATGACATATCTGATCATTTTGAAAATCCGGAGTTGGACACATTTGCTATTGACATGCAGGGTATAACTGGAAGCCAAAATGTTCATGATGAGGAAGAATGTATGGAGAAGGAAGAGGGAAATGAAAGTGGAAGCAAAGACCCGGGTAACAGTTTCTGGCACGAATTGTTGAATGAGAGTATTGATGAAGAAATTGGGATGCTAGGTGGTCAAGAAGAAGATGAGGATGTCGATGTGTTCGTCGAGGAGCTTGTTTACTTGGCCTCCAGTCCCAAGTAA

>PmHsf11

ATGAACTATCTGTACCCAGTGAAGGAAGAGTTCCCGGGTTCAAGTTCATCACAATCGGGTCCTGGTGACCCGGTGGTGATGATACCGCCACAGCCAATGGAGGGTCTGAATGACATAGGCCCTCCTCCATTTCTGACCAAGACCTTTGACATGGTGGATGACCCGAGTACCAATCGGATAGTTTCTTGGAGCAGAGGAGGTGGAAGCTTTGTTGTTTGGGATCCTCATCCCTTTGTTATGAATCTCCTTCCTAGATACTTCAAGCACAGTAATTTCTCAAGCTTTGTCAGGCAGCTCAACACTTACGTAAGTGTTTTCACCCCAAGCTGAAATCTTTAATTGGTTAGTGAAACCTTCAATCCTCTCATCAAGTTTTAGATCTTCAGTTTAAATGTATATGGAAATAATCCAGTTCATGTGGATGACCCTTCATTGATCTCTGGTATGTTGTTTGTCTGAGTCCCTTTCAATTTTCTTACCTATTTATTTTCAGCTAATTTATCAAACAAATAGAAATTTTTAAAAAAAATCTTCCCATTGACCTAAATGTTCTTTCGTCATGTTGAATTATTTCTCTTGAATCTGCTTAGAATTCATTTGGGTTGCTGAAGTTTCAATGTACTGGTGGTTTTCTGTAAATTATGAATGTTTTTTTCTTTCTTCTTTCTTTTTTGGTGAAAATAAAAGTTTACATATCTTTTGTTTGTGTATGGATGTTGTTGTAGGTTCTGGAATTTCATATGATTGTACTGTGTGCTCATGGAAATAAATTATTCTGTGTTTATCATCTGCTGTGTTTTCCCCTCCACCCTCCATTTTTTTTTAAATTTATGTTACTTTCCACAGTGCTTTCTTTATTTAATACTCTTGATTCTTTAATATCTCCTCCCAATGGCTCACCTTGCTCAATTTCTACTGGTGTCAAATCTCAAAAGTTAGTAATACAATATTCAGCACTATCTGAGCTCCTTTGTCAGGTGCAGGCTAATTGATTCCCTTAATTTCATTCCACCAATAATGATAGATATATTTATTTTTTGTGTCTATCAATTACTTGATGGGTATTGGGTGAAAGCGTTAATGGAGAAGCTTTAATAGATTTCTGAATCTGATTGGGCAATTAGGTTTGTAAAGTCATTGTTTCTAGGGAATTTAGTCCTAAATGTAATTGAACTTCTGCAGGGCTTTAATTGATTCCCTTAATTTCATTCCACCAATAATGATAGATTTATATATTTTTTGTGTCTATCAATTACTTGACGGGTATTGGGTGAAAGCGGTAATGGAGAAGCTTTAATAGATTTCTGAATCTGATTGGGCAATTAGGTTTGTAAAGTCATTGGTTCTTGGGAATTTAGTCCTAAATGTAATTGAACTTCTGCAGGGCTTTAGAAAGGTTGATCCTGACAGATGGGAGTTTGCCAATGAGGGGTTTGTAAGGGGTCAGAAGCATCTCCTTAAGAACATTAAAAGAAAGAAGACACCTTCTCAGCCTCTTCCTGCACAACAAGCTCTAGGCCCTTGTGTGGAAGTAGGGCGGTTTGGGCTAGATGGAGAAATTGATCGTTTGCGGCGCGACAAGCAGGTCCTAATGATGGAGTTGGTGAAGCTTAGACAGCAGCAGCAGAATACTAGAGCTTACCTTCAAGCAATGGAACAAAGGATACAAGGGACCGAAATGAAGCAGCAACAAATGATGGCTTTCTTGGCAAGGGCAATGCAAAACCCAGCTTTTATGCAGCAGCTAGTCCAACAGAAGGATAAAAGGAAGGAGCTTGAGGAAGCCATGACTAAGAAAAGGAGGAGGCCAATTGATCAAGGACCTAGTGGTGTTGGTGGTGGCAAATCGAGCCTAAAGGGCAAGGGAACAAACCTCATTAAATGTGAGCCTCTTGAATTTGGAGATTGTGATTATGAAATGTCAGAGCTAGAAGCACTTGCATTGGAAATGCAAGGATTTGGAAAGGCAAGAAAGGAACAGGATGAAGAGAGTGAGAGATTTGAGGGAGACTTAAGCATTCCAAGTGCAATAGTAGGGGAAGATGAAGATGTGATTATCTTGGCTGATCGCTTAGGTTACTTAGGTTCATGCCCAAAGTAG

>PmHsf3

ATGTTGAAATCGGCGGGGAAGAGTGGAGATGGGTCTGGGTCTGGTGGATCGGTGGCTCCTTTTCTGAGGAAATGCTATGAGATGGTGGATGATAACGATGCAGACTCTATAATCTCGTGGAGTGAAACCGGTGACAGCTTTGTGATATGGGACATGACCCAGTTCTCGATTTTATTGTTGCCCAAGTATTTCAAGCACAGCAACTTTTCTAGCTTCATGAGGCAGCTCAATATCTATGTGAGCTTTTCTGGGCAATTTTTTACTTTTTCTTCTATCTCGGTTATTGCATTTTGAGGTTTCTTTTTCATTATATGGAGGCGTTCTTTAGCTGCGTTACAGTTGTGGGTTGAATTGGTTTTTGTCAAATTGTGCTTATATTTATATTGGATTTTTAGACTTTTTTCTTCTTGTTATTATTGCGGCAATCCCAAATATAAATAATAAAAGAAAATTGCGCATTCGTAAATGATTGTAAACTAGAAGAAATCTCTTAAATCAAGTTTTCTTTCTTGCGTAGATGATTAGTTTGCTCTTTTGAGCCTAGACTTGTGCTCAATTGGTCCAAATCAAGTTTCGTATTTCCTTCATTTGGAAGTTTTCTGTGGTTATCCATTTAGGTGGGACTCAAAAGGGAAGGGTCTGTCTAAGGTGTAAGCCTTGGCCATGAGTTAAGACTTTGTAAAATATTTTGAATGGTGAAGTTCTCTTTTCGAAGCCTGATCCGTCTCCCTTCTTTTTGTCTCAGTTGGCCCTGAGCTAATTAAATGGTTGTGCTTCAGTCTCAGTTGCCCAGAAATGCTATATTGAGAAAATAGCAATGTATATAACTATATGCATTGTATATAGTATATAGCTATATGCATATGTATGTATATATCTGCACTACAGGTGCACTTGTGTGATTCTCTGTGAGTGTAAATGTCATGTTATGTTATTTGAATTGTCAACCAATAGTCAAGTATTCAAGAATCTTATTGTTTCAGGGCTTCAGAAAAATAGATTCAGATCGTTGGGTGTTTGCAAATGAAGGGTTTATTCGAGGTCAAAAGCATTTGTTGAAGAATATTTCTAGAAGGAAACATCCTCAGGGCACAGATCAGAGAAAAGCATTACAGCAGAAAGACAATCCTGATGGGCCTTTTGAAAACATTGTTGAAAATGGTCTATGGAGGGAAGTTGAGAACCTGAAGACTGATAAAGTTGCTCTGAAGCAAGAGTTGGTCAAGCTTAGGCAGCACCAGGAAATTTCAGAAAATAACTTGCTCCTCCTGAGGAACCGCCTTCGTGGAATGGAGAAGAATCAGCAGCAGATGCTGTCATTTCTAGTTATGGCCATGCAAAGTCCTGGGTTTTTAGTTCAGCTTCTTCAGCCAAAAGAAAACAGTTGGCGCATTGCTGAACCTGGAAATATGCTAGAACAAGGTGTAGATGATGGTATACCAATAACTTCTGATGGTGCGATAGTGAGATACCAACCTCCTGTGGATGAAGCCCCGAAGCCTATCCTCGCAGCGAATTCAGGCTCAGACAAACAAACTGAATTTGATTCTTATATAGATGGAATGAACGATTTTGTCGTGAATCCTGATTTCATGAAAATGCTAATGGATGAAAAGTTGAGCTCTCTGGAAAATCAAGCCCCATATACCCTACCGGATATATCTGATGATGGTGCATGGGAGCAGCTTCTTTTAGCTAGTCCTTTCTTAGAAGATATTGAAGCTACAAAGGAAGATGGAAAAGAGACTGTTGACTCTAGAATGGAGGTGGAATCAACCGCATCGGAGCTGCAAGAATCACAGAATTTTGATACTTTAATAGAGCAAATGAAGAAATCTCAGAACTTTGCATCGGAATCAACAGTTTATGGATCTAATGTGGAGAGCTCTCAAAACTTGGAACATATAACCGAACAAATGGGATATTTAGCTTCTGACTCTAACAGCAAACGTGGAACACAATCAGGAAAGTGA

>PmHsf15

ATGGCGCAAAGGTCCGTTCCGGCGCCGTTTTTGACCAAGACGTATCAGTTGGTGGACGATCCGAGCTGGGACGATGTCATCTCGTGGAACGAAAGCGGGACAACGTTTGTTGTTTGGAAGACTGTGGATTTTGCCAGGGATATGTTGCCTAAATATTTCAAGCACAACAATTTCTCAAGCTTCGTCCGCCAGCTTAACACCTATGTGAGTAATTAATTTCCATTATTTACCTTTTATTTTTGCGAGTACAAAACCAAATTATGTCTTAGAGTATTAGGCGAGTAGTCTACACCAAACGAAATTTAGTGTTATTTATCATCTTAAATTACATTGGTTTGGTTTTTTATTTTATTTTTAAATTTACTGTTTTTACATTTGCGCCAATATATGTTCAATATTACATATTAGTTCCAAAGATGAGATCAAAACTAGAAATTGAAAATTTTCCATAGAGATTCTTATTATTTTTTTAAAAATAGTTGGTGAAGCTTTCTTGGCAATTTAAAAAGAAAAAGAAAAACGTTGAAAATAATTGCCAATGGTCAATACAATTGTTTATTTGCAACTGGGTATTGGTTGAGCCTTTCCATTTTTTGTTGGTGTTGAAATTGTGACCAGGAATAAAATACAAATAATTATCAATCCATTAACGGGGAAACTGCGACTAAGTATTTTTTTAGATAAGAAAAAAAAAATTATGGTTGATTTTTTTGTAAAAAACATTTATATTAATCAAAGATGACATCAATTATGCAAAATTATTTGATGTAAATTGTAAGATAGTAGGATCTAGAAGAAAGAAAAAAATAAATTATGTTGTCTATATTATTCTATAATTTCTCTAAATTATCGGTTGTGCAACTCAATAATATAAAAAATTGCAAACGTGGCCCATCTGCCTCCATTTTTCCAAATACGACGGCGGAAAATCGAAGTTTCCAAATTAATCAAATATTCCGTTAACGTGTTAAATTTTAATTATCAATTAACTAAGCGTGCGTTTTGCTTCAATTACAGGGCTTTCGAAAGACGGTGCCGGACCAATGGGAGTTCGCGAACGACAACTTCCGGCGAGGGCAGAAGGAGCTCCTCGCCGAAATCCGTCGCCGGAAATCAGTGACGGCAGGGCCGGGGAAGGCTACTGCCAGCGAGAAATCCGGAGGGCCGTCGACTCCATCGAACTCGGGGGAGGAGATGGCGTCGACTTCGACGTCGTCGCCGGACTCGAAGAACCCGGGGGCGGTGGAGACGGCGGCAATGGGTCAAGCGTCTGATTTGTCGGGCGAGAACGAGAAACTGAAGAAGGAGAACGAGAATTTGAGCTCAGAGCTGGCGCAGACGAAGAAGCAGTGCGACGAGCTCGTGGGTTTCCTGATGGATTACCTGAAAGTGGGGCCCGATCAGATCAATCGCATCATGCGGCAAGGAAGCTATGTGTCCACCCGTGATGAAGATGAAAATGAAGATGATGATGCTGATGATGATGATGACGACGACGGAAAACAAAAAGAGGGCTTGAAGCTGTTTGGGGTTTGGGTGAAAGGGGACGAGAAGAAGAAGAGTAAGAGGACGGAGCGAGATGAGAAATTTGGGGTTGGTGTTGGTGGGACCTACGCGAAGAAGATGAAGAGAGCGGAATTCGGCGCGCCGATGTTGAAGAGGGGGAAGGTGTGCAACTGA

>PmHsf6

ATGGCTGCTACAACTTCTTCAGGCCAATCTCCGAGGACAAGAAGCCCTGCCCCTTTCTTGTCCAAGACATATGATTTGCTAGAAAAAGGTGCAGCAGAGGAAGGAGATAGCGGAAAGAAGATTGTGTCTTGGAATGCAGAGGGCTCTGGATTTATAGTTTGGTCTCCTGCTGAGTTCTCAGAGCTCCTGCTGCCTAAATATTTCAAGCACAATAATTTCTCCAGCTTCATCCGCCAGCTTAATACCTACGTAAGTCGTCGTCTTTTCCTTGTTATTCAACAATTGTCGTCTATGAGATTTGCGCTAGGCCCCTCTTTCTTTTACTAATCTTTGAAGTACTTCTGATTACTTAGAATGCTTTTTGGTTATTTTTGATCAGTTTCTCAAGAACCCAGTATTTCATATGTAAATCATAATATTTTGAGTGATGGGTTTTCTTTCTTTTCTTGTGTGTGCCTCACCATGTACACAGCAGTCAAACTAAGTTAACAGAAAATATAATAATGTGGAGAAAGTCAAAACATGTGAAAGTTGTAATTGGTCAAAATAATCACGCTCTGCAGACTGATTTATAGTATCATACAGCATCACAGCTCCTTCATCTATAAACAGTAACATAAAAAAAGCGTGCACCTCTCTCTGCCATATCCTGTTTTTACTGTTTTCCTTTTTCTGCTTATTTGACCTACCAATAATGTACTTGATATCTTAATGATTTTCAATCATTTATGTTCCTATCAATGTGCATCCGTGTGTGTATTTTAAATACAGGACAGTCTAATTAGGAAGCATTAGCAACAATGGTCAAATTTGGTTTTGGAATAAACAATTCTATGCACCTTCGAAACAACGTTTGTCTGATGCTAAAAGTCCAAAAACAAAATTGTTGTCTCAATCCATGCATAATTAATTGGAGAGTTTGGTTGGTTTTTTGATTTATGAATCTGACCCTTGAAGCCAAAAGCAGGGGTTCAAGAAAACATCACCAAAACAATGGGAATTTAAGCATGAAAAGTTCCAGAAAGGCTGTAGGCATATGCTGGTGGAGATCACAAGGAAGAAATGTGAGCCAAGTGCATTTCCAGTGTATCTAAAGGCTTCAGAAGAGAGTGGTAGCAGCAGTACAACTGTGGCTGCAGCAGAGGAAAATAATCGCTTGCTGCTAATGGAGGAGAACAAGAACCTCAGGAAACAGAAACTGGAGCTGCAGATGCAACTATCTCAGTTTAAAGCCTTAGAAATGAAGCTGTTGGATTGCCTAGCGCAGAACATGGAAGATCATCAGAATAAAGTTCGATGCTGA

>PmHsf10

ATGGCTCCGACGTCGGTGGAGCCGAACGGCGGTGAGTCCACATCCAGCGAGTCATCCCATAGAGCTTTACCGACACCATTTCTGACCAAGACGTATCAGCTGGTCGATGATCCCACAATCGACGACGTCATCTCCTGGAACGACGACGGATCTAGCTTTGTCGTGTGGAACCCCACCGTCTTCGCCAGAGATTTGCTCCCCAAGTATTTCAAGCACAACAACTTCTCTAGCTTCGTCAGGCAGCTCAACACCTACGTAAGTCCTCTATTTGTCTTCTTCAATTTCCGATTGAATTTATAAAAATAATAATATTTCAGTTATTTATTTGATTATTTTTGGATTTTTAGGGATTTAGAAAGGTTATACCAGACCGTTGGGAATTCTCGAACGATTGCTTTCGAAGAGGCGAAAAGCGGCTTCTCTGCGAGATACAGCGCCGAAGAATCATGCCTCCGGCGCCGGCGGTGGCCGTTTCGCCGATGGCGACGGCAGCGGTGGTTCCGAATGCGAAACCTATGATATCTCCGTCGAACTCCGGCGAGGAGCAGGTGATTTCGTCGAGCTCGTCGCCGATCAGAGCTCCGTCCGAGCTCATGGACGAGAACGAAAAGCTGAGGAAGGAGAACATGCAGCTCACCAAGGAACTGGCTGATGTCAAGTCTCTCTGCAACAACATCTTCAGCATGGTCTCGAATTACGCGTACGCACAATCGGAAAGCGGTTTCCCATATGTGAAACCGCTGGATTTGATGCCTGAGAAGCGGTTCTCCGGCGACGGCGAGAAGGAAGAAGAGGAGGCGAGCCCGAAGCTTTTCGGTGTGGCGATTGGAGCCAAGCGAGCAAGAGAGACCGTCGGCGACGGTGTAGAGGAGGATGAGACCGGCTTACGACTGCAGCAACCGAGTGGTGGTGGCGACGTTAAATCAGAGCCGTTAGATGTGGATCGTCAGGAAACGCCGTGGCTGAATCAGCGCCACATGGCTAATCAGAGGGTGTGTAATTAA

>PmHsf13

ATGGCGTCGTTGCAGGCCGACCAGAACGGCGACTCCGGCCCCGTTAACGGCGCTGGAGGAGGTGGAGACTCACAAAGGACGCTTCCGACGCCGTTTTTGACCAAAACGTATCAGCTGGTGGACGATCCGTCCGTCGACGATTTGATCTCGTGGAGCGAAGACGGATCGGCCTTCATAGTCTGGCGACCCGCCGAATTCGCCAGAGATTTGCTCCCCAAGTATTTCAAGCATAACAACTTCTCCAGCTTCGTCCGCCAGCTCAACACTTACGTAAGCCCTCTCTGTTTCCCTCACTTTCCTCCTCTTTCTCGGCAGCCAAACAAGCTTAAGCACGTAAATGTTCTATTTTCTAATCGATTTCTATTGTACTTACAGGGATTTCGGAAAGTTGTGCCGGATCGGTGGGAGTTCGCGAACGATTGCTTCAAAAGAGGTGAGAAAGGCCTCCTACGGGAGATTCAGCGCCGGAAAATCTCGCCGTCGGTGTCGGCGTCGCCTGCGGCGATTACCGCGACGTTGGCGACGGTGTCTGCGGTGGCTCCCGGGGTATCTCCGTCGAACTCCGGCGACGAGCAAGTGATCTCGTCGAACTCGTCGCCGGTGGCGCCTCCGGCTACGATGTTGAGCCGAATCCGGAGCTGCACCACGACGTCGGACGTTCTGGAAGAGAACGAGCGGCTCAGGAAAGAGAACATGCAGCTAAGTCACGAGCTGACTCAGCTGCGCGGCTTGTGTAACAATATATTGGCATTGATGACGAACTACGCTTCTGGTCAGTTGGAGGGCGGCGGCGGTGGCGGTGGCGGTGGGAGTGTAATGGATGAGGGTAAGCCTCTGGAGCTGTCGCCGGTGAAGGAGGCTGAGCCGTCTGAAAACGGCGTCGTTCGCGAGGGGTCTAAGGCGGAGGCTTCGGCGGAGGAAGAGGAAGACGAGGAGGAGATGAGGCCGAGGCTGTTTGGGGTGTCGATTGGGGTGAAGCGCGTGCGGAGAGACGAGGAGGAAGAGGAGCAGCATCGGGAAGGGTCTGAGGCGATGAAATCAGAGCCGTCAGATGGGAGTTCGAAGCGCGACCAGGACTCCACGTGGCTGGAGCTCGGAAAGTGA

>PmHsf18

ATGGAGGGTGTGTGTGACCAGAAGGGTTTGCTGGAATATGTGAGGAAGTCAAGCCCTCCACCTTTCTTGTTGAAGACCTACATGCTGGTGGAGGATCCTGCCACCGACGATGTGATCTCCTGGAACGATGACGGGTCGGCGTTCGTGGTGTGGCAGCCGGCGGAGTTTGCCAGAGATCTCCTCCCAACACTCTTCAAGCATAGTAACTTCTCTAGCTTTGTCAGGCAGCTAAATACTTATGTATGTACAAATTTATTAACCATATATTTTGCATGCTTTTTCTAACCAATATCAAATTCTTGCACTTTGCCATCTCAAATGCATTTTTTCTTGCCATATATTTTCCATCATCATCATCATCATGTCCAAAAAAAAAAAAAGTCTTCATTCCAAGTTGACCGAGTTCTAAGATTTGATTTCGAGTTAATATGAAAAGGTCCACCGGTCTTGTCTAACCTAACTTAAAATATACTAGTTTTTCGGTGTTTTGATGGACCAACCAAATTAAATTAAATATATATATTTATATATATTTGTGTTGGGTTTGATTTGAACTTTAATTGGAGTAGGCTGAGCCATAAAAGATAAAAAATGAACTGTAGACTCATGAGTATGACAGCGGCCAATGTCCCATAAGAAAACCATAATGTCACACAACCTACTTACGGGCACAGAAGCATTTTTCTTTCATCATTTATTTGGTTGAAAAGTAGTTACGCTGCTGATAATTTCTTCATATATCTTATTCTATGTTCAAGATTATTTTTAACAACTATAGATGGAAGACTTGAATTAGCGCCTCTTTATAGATTATGAAGAGAAATACTATTTAGATTAGTGATTTCAGACATAGGCTCGTTTAGTGCCTACCTAAGATTGGATTAGATTGGACAGCTCAACGCATGTCAATTGAAGAAATAAAATTGTCCAAGTTGGAGGTAGAAGTAGAGGACTTAATCATGAATAAAACTTATCTCATTCATTCCCCAAAAAATGGTAGAATATCAATATCTCATAACGTATCCCTTCTAATCTTCCAAATGAAAAATCATTCACCTCTATCATCATTTCCTTTTTCAACATATACTTGAATCTAATGAGTCATCTAATCCTAGACACCAAATGAGAAAATATGATTTTACCCTCCTTCACATATCTCTCTTCTAATATAACTTATCTCTTATATTTCATTTCTCTATCTTTACTTAAAACCAAAACAAAAAATTTACGCTGAATTTTAATAATTTTGCACATATGTCTAGGGATTTCGGAAAGTTTCAACAAGCAGGTGGGAGTTCTGCAACGACAAGTTCCGAAAGGGTGAAAAGGATCAGCTATGTGAAATCCGCAGAAGAAAAGCATGGGCCAGCAAGCAACAGCCAATCAACAACATTGCTCTAAACCAAGCTGCACAAGCAATGCCAAATCAAGACGAGTTTGATGAAGACCAGAGATCAAACTCCTCAACCTCCTCCTCATCTGATTACAGCTCTCTCGTCGACGAAAACAAACGGCTGAAGCAGGAGAATGGGGTTTTAAGCTCAGAGCTCACCAGCATGAAGAGGAAGTGTAAGGAGCTTCTTGACTTGGTGGCCAAGTGTGGAGACTCAGCTGAGAAAGAAGAGGAGAATAGTGAGAGGGTGCCCAAGTTGTTTGGAGTGAGATTGGAAGTGGAGGGGGAGACGGAGAGGAAGAGGAAGAGAGCTGAAATTAGTGAGAGCGCAAGCATTTTACTATCTCAAGCATGCAAATAA

>PmHsf1

ATGGCTCTCATGATGGACAATTGTGAGGGCATATTGCTTTCCCTGGACTCGCACAAGTCGGTGCCGGCTCCCTTCCTGACCAAAACGTACCAGCTCGTGGATGATCCAGCCACCGACCACATCGTCTCGTGGGGAGAGGACGACGCCACCTTCGTCGTTTGGCGCCCTCCCGAGTTCGCCCGGGACCTCCTCCCCAACTACTTCAAGCACAACAACTTCTCCAGCTTCGTCCGCCAGCTCAACACCTACGTACGTGATAACATACATAAAATTCATCACACAATTTTTTTTGGTTTTGTTCCTGTGTTTTATTTGATTTTCACTGACGGAGTCGTTTTGTTGTTATTGGGGTTTCTAGGGTTTTAGGAAGATTGTACCGGACAGATGGGAGTTTGCGAACGAGTTCTTCAAGAAAGGAGAGAAGCATTTGCTCTGTGAGATCCATAGAAGAAAGACAGCTCAGCCTCATCAGGTGGGTTTCAGCCACCACCACCACCACCACCACCACAACCCGCATTCGCCACTCGGCATCAACGGCCACCATCATCCGAGCTTCTTCCCCTTCCCGAGTAGTGGCAGCATCTCCCCCTCCGACTCGGACGAGCCGCCCAACTGGTGTGACTCGGACTCACCACCACTCCCATCCCCAACCGGAGGTATTAACAATCACAACAACAACAATAATAATTTTATGAATATTAATAATGCGTCGGTGACGGGCTTGGCGGAGGACAATGAGAGGCTGCGGCGGAGCAACTCCGTGCTGATGTCAGAGCTAGCCCACATGAGAAAACTCTACAACGACATCATCTACTTTGTTCAGAACCATGTCAAGCCTGTGGCTCCAAGCAATTCCTACCCTTCTTCTTTGCTTCTCTCTAACCCTCCTCCGAATTCCATGGCTCCAGCTGCTACTGCTACTAAGCCTAGTAATTTCAACCAGCTTCTTGGGTACTATCCAGCTCCTGCTACAAATGCTAAGCAAACCCCTCACATGTCTACGACGATCCACCATCATGTTATGAACTCTTCCAGCCCGAGCAACACCACGTCCAAGAGCAGCTCAGTGACTATTCTTGAAGACCAGCAACAACCCAGTAGCAATGGGTGCAAAAATACCAAGCTGTTTGGGGTGCCGCTGCTTCACTCGAAGAAGCGGTTGCACCCGGAGGAGTATGGCTCGAACCATGGGACCAGCATGATGGAGGCCAGCAAGGCTCGTCTGATTTTGGAAAAAGATGACTTAGGTCTCCATCTCATGCCTCCCTCCGCATGTTAG

>PmHsf17

ATGATGATGGAGGAGAACAACAACGTCATCGCGCCGTTCGTTATGAAGACTTACCAGATGGTCAACGATCCGACGACGGACAAGTTAATCTCTTGGGGCCAAGCCAACAACAGCTTCATCGTCGTCGACCCTTTAGACTTCTCTCAGAGGCTCTTACCCGCTTACTTCAAGCACAACAATTTCTCCAGCTTCGTTCGCCAGCTTAACACATATGTGAGCATATATAACACATATAATTGTACACTGATTTGATTAATTATCGGTGAAAATGAAAACCCATTTCTCAATTTTGATTATACGTGCAGGGATTTCGAAAGGTCGATCCAGATAGGTGGGAATTTGCGAACGAGTGGTTTCTGCGGGGCCAAACGCATTTGCTGAGGAACGTGGTGAGGAGAAAGCACATGGGTAAGAATTCGTATTCGAATTCGAATTCGACCACGTGCTTATTACAGGGGAAGCACGAGGAGCTTGACGACGAAGAGATAGTGATGGAGATTGCGAGGTTGAAGCAGGAGCAGAAGGCGTTGGAAGAAGAAATGGAGAACATGAACAAGAGATTGGAGGCAACTGAGAGACGACCCCAGCAAATGATGGCTTTTCTGCACAAAGTCGCGGAGGACCCTGAGATTTTGCCACGTATTATGCTCGAGAAGGATCGTACGTTCAGGGCGCAGTTGGGGGAGAAGAAGCGGCGGGTTATGATGATAACCTCAACGTCGTCGTCTTCGTTGGGCATGGGGGCCACCAACTCCGTCGAGACCGAGGATGAGGATGACGGAACCGTAGGGGTAATTTCGTCATCTCCCGAACCGGGTTTTGAGATGGATAGTTTTTATTCCACGTCTCCAGAGACGTCGACGGCCCGAGAGTGGGGGAGGCAGAGGCGGGGCGGGGGGCTGGGTCGGGCTGTGCAAGACCCGTATAATATGAACCCGACGGTGTCGGGTCATGGGATCGGGAATACTAGTAATTCCGGGTACGGGTATGGGAATAGAAATGGTGGTGCGGAAGTGGGTTATCTCACAGAGGAACCTACCCCTGCACCACCGCCTTATCCATTCTCGTTGTTAGAGGGTAGCTTTTAG

>PmsHsp1

ATGTCGATCATCCCCAGCTTCCGACGAGGCAGCATTTTCGACCCTTTCTCTCTCGATGTCTGGGAGCCATTCAAAGATTTCCCATTCCCTTCCTCCTCATCACTCTCCACATTCCCTGAATTTTCCCGGGAAAATTCAGCTTTCCTGAACACGAGGATCGACTGGAAGGAGACCCCGGAAGCCCACTTGTTCAAGGCAGACCTTCCGGGGCTGAAGAAAGAAGAAGTGAAGGTGGAGGTTGAAGACGACAGGGTGCTTCAGATCAGCGGAGAGAGGAACGTAGAGAAGGAGGACAAGAACGACAAGTGGCACAGAGTGGAGCGCAGCAGCGGCAAGTTCTTGAGGAGGTTTCAGCTTCCTGAAAATGCAAAGGTGGACGAGATTAAGGCTGCAATGGAGAATGGGGTTCTGAGTGTCACTGTTCCAAAGGCAGAGGTGAAGAAGGCTGATGTCAAAGCCATTGAAATCTCAGGTTAA

>PmsHsp3

ATGTCGATCATCCCCAGCTTCCGACGAGGCGGCATTTTCGACCCTTTCTCTCTCGATGTCTGGGAGCCGTTCAAGGATTTCCCGTTCCCTTCCTCCTCATCACTCTCCACATTCCCTGAATTTTCCCGGGAAAATTCAGCTTTCTTGAACACGAGGATCGACTGGAAGGAGACCCCGGAAGCCCACTTGTTCAAGGCAGACCTTCCGGGGCTGAAGAAAGAAGAGGTGAAGGTGGAGCTCGAAGAAAACAGGGTGCTGCAGATCAGCGGAGAGAGGAAAATAGAGAAGGAGGACAAGAACGACCAGTGGCACAGAGTGGAGCGCAGCAGCGGCAAGTTCTTGAGGAGGTTTCAGCTTCCTGAGAATGCAAAGGTGGACGAGATTAAGGCTGCAATGGAGAATGGGGTTTTGAGTGTCACTGTTCCAAAGGCAGAGGTGAAGAAGCCTGATGTCAAAGCCATTGAAATCTCTGGTTAA

>PmsHsp5

ATGTCGATCATCCCCAGCTTCCGACAAGGCAGCATTTTCGACCCTTTCTCTCTCGATGTCTGGGAGCCATTCAAAGATTTCCCATTCCCTTCCTCCTCATCACTCTCCACATTCCCTAAATTTTCCCGGGAAAATTCAGCTTTCCTGAACACGAGGATCGACTGGAAGGAGACCCCGGAAGCCCACTTGTTCAAGGCAGACCTCCCGGGGCTGAAGAAAGAAGAGGTGAAGGTGGAGGTTGAAGACGACAGAGTGCTTCAGATCAGCGGAGAGAGGAACGTAGAGAAGGAGGACAAGAACGACAAGTGGCACAGAGTGGAGCGCAGCAGCGGCAAGTTCTTGAGGAGGTTTCAGCTTCCTGAGAATGCAAAGGTGGACGAGATTAAGGCTGCAATGGAGAATGGGGTTCTGAGTGTCACTGTTCCAAAGGCAGAGGTGAAGAAGCCCGATGTCAAAGCCATTGAAATCTCTGGTTAA

>PmsHsp6

ATGTCGATCATCCCCAACTTCCGACGAGGCAGCGTTTTCGACCCTTTCTCTCTCGATCTCTGGGAACCCTTAAAGGATTTTCCATTCCCTTCCTCCTCATCACTCTCCACATTCCCTGAATTTTCCCGGGAAAATTCAGCTATCCTGAACACCAGAATCGACTGGAAGGAGACCCCAGAAGCCCACGTGTTCAAGGCAGACCTCCCGGGGCTGAAGAAAGAAGAGGTGAAGGTGGAGGTTGAAGACGACAGGGTGCTTCAGATCAGCGGAGAGAGGAACGTAGAGAAGGAGGACAAGAACGACAAGTGGCACAGAGTGGAGCGCAGCAGCGGCAGGTTCTTGAGGAGGTTTCAGCTTCCTGAGAATGCAAAGGTGGACGAGATTAAGGCTGCAATGGAGAATGGGGTTCTGAGTGTCACTGTTCCAAAGGCAGAGGTGAAGAAGCCTGATGTCAAAGCCATTGAAATCTCTGGTTAA

>PmsHsp7

ATGTCGATCATCCCCAACTTCCGACGAAGCAGCATTTTCGACCCTTTCGCTCTTGATCTCTGGGACCCCTTCAAGGATTTCCAATTCCCTTCCTCCTCATCACTCTCTACATTCCCTGAATTTTCCCGGGAAAATTCTGCTTTTCTGAACACTAGAATAGACTGGAAGGAGACCCCAGAAGCTCATGTGTTCAAGGCTGACATTCCGGGGATGAAGACGGAAGAGGTGAAGGTGGAGGTTGAAGACGACAGGGTGCTTCAGATCAGTGGAGAGAAGGATGACAAGAACGACAAGTGGCACATGGTGGAGCGCAGCAGCGGCAAGTTCTTGAGGAGGTTTCAGCTTCCTGAGAATGCCAAGGTAAATGAGATTAAGGCTGCAATGGAGAATGGGGTTCTGGGTGTTACTGTTCCCAAGACAGAGATGAAGAAGCCTGATGCCAAAGCCATTGAAATCTCTGGTTGA

>PmsHsp4

ATGTCAATCGTCCCAATCAACGAGCAACGAGGCAGTGACTTCGACCCTTCCTTGGCTCTTGACCTGTGGGACCCATTCACAGATTTCCCTTTCCCATTCCCTTCCTCACTCTCCAACGTTTTCCGGGAATTCAATCTGGGTTCTTCGGTAAACTCAAGACTAGATTGGAGAGAGACCCGAAATGCCCACATCCTAAAGGCGGCTCTTCCTGCGTTCATGAACGAGGACGTGCTTGTGGAGCTTCAAGACGAGCGTGTGCTTCAGATCAGTACAGACAGCGGTAGCTTCATGACAAAGTTTAAGCTTCCGGACAACGCCAAGATTGAGCAGCATAAGGCCTTCATGAGCAATGGGGTTCTCACTGTCACTGTTCCTAAGGAGGAGCCCAGCAGGCCCAATATCAGAGCCATTGAAATTTCTGGCGAAGATTAA

>PmsHsp8

ATGTCAATCGTCCCAATCAACGAGCAACGAGGCAGTGACCTCGACCCTTCCTTGGCTCTTGACCTGTGGGACCCATTCACAGATTCCCCTTTCCCATTCCCTTCCTCACTCTCCAACGTTTTCCGGGAATTCAATCTGGGTTCTTCGGTAAACTCAAGACTAGATTGGAGAGAGACCCGAAATGCCCACATCCTAAAGGCGGCTCTTCCTGCGTTCATGAACGAGGACGTGCTTGTGGAGCTTCAAGACGAGCGTGTGCTTCAGATCAGTACAGACAGCGGTAGCTTCATGACAAAGTTTAAGCTTCCGGACAACGCCAAGATTGAGCAGCTTAAGGCCTTCATGAGCAATGGGGTTCTCACTGTCACTGTTCCTAAGGAGGAGCCCAGCAGACCCAATATCAGAGCCATTGAAATTTCTGGCGAAGATTAA

>PmsHsp2

ATGGCTTGTAACTTGTTCGTTTTTAATATAAATGGATCGTAATTGAGGCTTTGACAGGTTCCTGTTTTGAATTGCATTTGTATGTGTTGCGTTTCTGTTTTCAGATCTCTCAGTTCTCTATTTAAAGTTACGTTTACTTTTGTTTCTGAATTACAGTCTTTTGATACCTTATCATGACTCTGTTCGCTTTTGCTTTCCCCAAATCTTAAAAACATTTGGAAAACCGAGTAACGCGGAACGGCTATACTATTATAGAACATTTTTAAAATGACGTATAGCCGTTCGGCTATACTATTTATTTAAAAAATAAAATTAGCCGAGTACAGCCGGCCGGCTGTACGATTTCTCTAAAAAAATTGAAAAAACCCCTGTATAGCCGACAGGCGGTACTATTTGTTTTAAAATCCTCCTCTCCACATTTCCCGAATTTTCCCAGGAAACTTCAGCTTTCTTGAACACGATGATCAACTGGAAGGAGACCCCAGCAGCCCATGTGTTCAAGGCATACCTTCCGGGGCTGAAGAAAGAAGAGGTGAAGGTGGAGCTCGAGGACGGCATGGTGCTTCAGATCAATGGAGAGAGAAATGTAGAGATGGTGGACAAGAACGACAAATGGCACAGAGTGGAGCACAGCAGCGGCAAGTTCTTGAGGAGGTTTCAGCTTCCAGAGAATGCAAGGGTAGACGAGATTAAGGCTGCAATGGAGAATGGGGTTCTGAGTGTCACTGTTCCCAAGGCAGGGCCAAAGCCATTGAAATCTCTGGTTAATATCGTCTCTGTATTCTCTACTGCAATTCACATTATGTATTTCTTTGTTAGATTTTGGAGTGTCAGTTGTCATCAAAAACCACAGGGACACGCATGCCCCAACAGGTAAAAAAGGAAAGGGATTGAACTGTCCAGAGGCAGCCCCGCCTCCCAAAGCTTGGAAAGATTTAGCCCGTGGCCCAAGCTAGCAATAGCATCAGTCGTGAAATTAGCCTCACGGAATACATGACTGAAAGACCAAGAATCACAAAAGGAGCTGATAAGCACTATATCTTTAATCAAGAGGTTGATACTCCACAGGGTCAAAGCTTTCTGATTAATGCAATCAATAATAAGTTTAGAATTTCCTTCAACAAGAACTTTGCGCCATCCTCTATGAGTTCCATAAGCAAGGCCATCCCGTAA

>PmsHsp9

ATGGCACTAATTCCTCGTACCATTTTTGTTGGTCACTATGATCCTTTCTGCCACGATGTTTGGGACCCATTTCAGGAATTCCACTATGGATTTCCAAGAGAAGCAACCTCCTTCACCAACTCCATGATTGATTGGAAGGAGACCTCAGATGGGCGCGCGTATGTACTCAAGGAAGATCTTCCAGGCTTCAGGAGAGAAGAAGTAAAGGTGGATGTGGAGGAGGGTAGGGTTCTTCGCATTAGGGGGGAGAAAAATGTGGAGAGAGAAGAGAAGAAGGACCATTGGCACCGCATCGAAAGGAGCAGCGGCAAGTTCATCAGGCGTTTGAGTTTGCCTGAGAATGCCAAGGCTGACAAGATGAAGGTGTTCATGGAAAATGGAGAGCTCACTGTCACGGTTCCTAAGGAGAAGGTTAATTTCTATCCTCATGCAACCAGAGCCGTTCAAATTTCTGGGCACTAA

>PmsHsp16

ATGTCTCTCTTGCAGTCTCTCTTGGACCAACCCAACTTCTTGTCTCCTCTCAGAGTTTTTAACTCAGACATAGGCTACAACAACACCTACATGGACTGGAAGGAGACTTCACATGCCCACATCTTTGAAATTGATCTTCCAGGCCTTACAAAAGAGGATGTGAAGCTTGAGGTGCATGAAAATAGAGTGCTTCATGTGAGTGCAGAGAGAAAGGCAGAGCCTGAAGCAGAGGACCCCAAGAATGAAACGTGGCACTGCAGGGAAAGGACAAGTGACAGCTTCTCTAGAAACTTTCGGCTGCCTGAAAATGCCAAGGTTGATGAGATTAAGGCTTCCATGCGTGACGGGGTTTTAGTAATTACAGTGCCTAAGGAGGATGACTTGAAGAAAAAACACAAGCATCACAAGAAGGTTGAGATCTCTGGAGATGATGAAAAACATGGCTCCAAAGGGCTCGGACGCTTTGTGTGCTGCAAAGCTTAG

>PmsHsp18

ATGGCTGAGTGCACTAGGGACAACTCATTGGCATTGCTTTGCAATCTATGCACATGGCTACGGAAATTTATATTCTCTGACTGCCCTTTAAAGAAATGGGGTGTTCTTTTCCGTGCGTTGTAGTTGTTGGAAATGCCTTATTTTTTATTATAGAGAAATGTGGTAGTATTTCGTTGGAGAAAAATTAACTAGTACTTTAAACGAGTTATATCGTTGAAATGAACTTAGTTGTCGGCCTAACTAGAGAGCCTTATTTTTATGTTTTTTCAATTGTGCCTTTGGCCAGAGAGATATCAAAGAGGAGATGGGCAATCCAAGCAATTCATTGGTAAGAGAAACATTGGAGAGGGTTTAAAGAGAAATAAACAAAGAAGACAATAGAAGAAAGTTGAAATTAGAACCTTAAGATATGGGTGGCATAATATTTAAAAATAACATCATCCTATATATCAAAAGTGTTGATACGTGAATACGAAACACTTACAGTTCAAGTGGTTAGAGCCCGCATTTTATGTCTTATAGTTCAAGTGGTTAAAGCCCACACTTCATGTCTTTAGGTTCGATTTCTCCTCTCGCAATATCACTTGTATCAAAATGTATTTTAATTATTTTAAATAAAGTTATATATGTGAGGTGGTTATAGCAATTTGGGCTTCTTTCTCTCTTGGGCTATTTCCCCTGGTTTTGTTAACTTTGTTTTTTTTTTGTTGGGCATTCCCGTTGGGCTTTAGCCTCCAGTTTGTTGCCTTGTTTCTTGAATATGGGCTGGACCCTTATTCCACAACAGTCAACAAGTAAAAAGTCACAATAGCCCGTATATTTGCATGTAAGTGGATCTTATGCCACTTGTCTCTGGATTGCTATAACCACTTCACATATATCTTCTTTTTTTTCGCCACGACAGTCAACAAGTAAAAACTCTCTGGATTACGAAAATGCCCGTGTCTTCTCCCTACAGATTCCTCATCTCCCCTCCCAACGTACCAACGCAGAAGAGCAATTAGCCAAAGCAATTCAAACGAGTTCAATTAGCCAACAAATCAAACACTCTTNNNNNNNNNNNNNNNNNNNNNNNNNTCCAAGCTTCTTCGGCGGCCGAAGGGCTAACGTCTTCGACCCATTCTCTCTCCACATCTGGGATCCATTCCAGGACTTCCCGTTGATCAGCGGCGGTGGCGAACACGCGGATCAACTGGAAGGAGACGCCGGAGGCGCACGTGTTCAAGGCGGACCTTCCGGGGCTGAAGGAGGAGGTGAAAGTGGAGGTTGAGGAAGGAAGGGTGCTGCAGATCAGCGGAGAGAGGAGCAGAGAGAAGGAGGAGAAGAACAAGTGGCAAAGGGTGGAGAGGAGCAGCGGCAAGAAGTTGTTTTCTCAACTGTAATTTGCAGCATTTGCTAGGTTTCCTTACGCCCTCCCCACCGACATTTTTCAAGCACAACCCAACTACATTTTATGATACAAAAGATGGACCATCATTTATTAAAAGGAACAGGGAAACAGAGTCTCACTTTCAATCGGTTTGCTGTTCGCTTCTGGTTTTGTAATAAAGCACACATACAAACAAATTCATGATCACTCACTCTGCTTTCCAAAAGCAAAGGATTTTATTGATAAAATATGTTTAACCAAGCCATCCAAAGATTGGTATGAAGAGCCACCTTCTTTGAGGCAAAGATTTGCCTTCTCCATTAGCTCCATTGCTCTGTCTCTGATCTCTTCCCCTTCTTTCTCGACCATTAACAAATTAATGGTTCTTTCAATCTCAGCTCTCTCGATGCCATGCTCTATCTGCACCCCAACCTTCCAAACATGGCTCACATATCTTGCATTGGCCATTTGATCACCGAAACATGGCATGCAAATCATAGGGACCCCCTCACAGATACTTTCCAATGTAGAATTCCAGCCGCTGTGAGTCCAAAACACTGCAACAGATGGATGGGCCAGCACTTCTTTTTGTGGAGCCCATTTCACAATGTGACCCCTCCCATTCAAACTCTCGAGAAACCCATCAGGCAATGGTTCTGATCCATGGACTAATCCGGGTCGAACCACCCACAAGAATGGATGGTTGCTGTTGGCTAGTCCCCAAGCTATCTCCAAAAACTGAGCCTCTTTGACTGCAGCAATGCTCCCAAAGCCAACATACACAACAGATTTCGGTGCTTGAGTGTTTAGCCATGAAATGCAACTCTGGTCTTCTGCTAATAAGCCAGTCGAAGAAGACGGTGCAGAGTCACACTTGTGAAATGGGCCTATTGGGAAAATTGGAATATTGGGGTAAGTTTCGTGGCGAAGTGTGGCAAGTGCATGTTGTTCAAGGTCTTCGAAAGTGTTGAAAATCAGTCCAGATGAGGCCTTGGGTTCATTTGCCATGTTGGTTATCAGTTGGTAAATACTCTCAGGGTCAGAATTGTCCTTCCCGGGCAGGTCTTTAACTTTGAGAGGTGACAGCTCTGTCACCGGCTCTTCCAGCCGAGAATCTAATCAAGGTCAAAAACAGAAACTAGAAATTATATAAGTTTGTCAAGTCCATGCAATTAGGCAATGAACAAAGAAAACCAAGAAAGAAACTTTGAAATTGTACCTTGAATTGGGAGGTAACCCTTTTCCTTCAAGAGTGGAAAAGCAGCATAAACAACAAGGGAAGTGGCGCCCCCAGTCCTTAGCAAAATCCTTGGGAGCTCAAAGCTCTCTGCAACTGATCGAGTGAAGTGAAAGAAAGGGTCTGAGATCAAGCAAGCAACAGGATCCTCCTCTGCATCGGATAACAAGGTAGCCAAGCATTCCCTGAAAGGTTCAACACATTTAGCGTTTAGAAGAGAAACAGGACGGCCAGCTTTTTCCGATTCAGATAAGTCGACAGGGATTGAGTGGAAGGTGAAGTGTGGGTGGCTTGAAGGGTTGAGAGAGTTGAACTTGGTGTGGACGATGGTTATGGAAAAGCCTTTGGAGTGAAGAAGGTTGCCCAGTTCTAGCATGGGGTTTATATGGCCTTGGAATGGCAGTGGGAGAAGTATCAATCTCAGGCCTCTGCTTTGCTCCATTAGAATTCAGAATCACTGACTCTGTTTGCTTGGCTTCTGATTCTGAGATTGTACTTGTGAGTTCTTAGAGGCAATATGTTTTTCTTTATTGTGAGATTCATATGACTGGTCTACGGGTTTGAATTCAAAGTAAGATATTTTTCTCAGCCGCTGAGCTCACGACTTACAAATTTTAGGTCCTTAGATTAATACGTACTTTTGGATCAATTTGAGAGCCATACTTATCCTTCATAAACATTTAGTCACCCACAGATGGCAATGTTTGCTAACACTTGAATATGTCAACGAGGTGGAAGAATACAAGTTGGCTGTCAATTCTCTTTCGCTATAATAATTTTTTTTTTAAACATCCTTCTCCTCCCGCAATTATTATAGCGATATGGGCTTCTTTCTCCCTTCTTCTTTCTTGAATATGGGCTGGATCCTTATTCCACAACAGTCAACAAGAAGTCACAGTCGCTCCTTTATACCACTTGTCACAATCCAGAAAGGTCTCTCTCTGTCTCTGGAAACCTCTTCCCCCCTACAGAAAATACCAGACACTTCTCTTCGCTCCCTCCATCCTCCTGAAATATCAGGAAGGGACGAGGAACAGTAACACAATTACGAAAATGCCACTGTCTTCTCCCTATAAATATCTCATCTTCCCTCCCAACGTACCAACACAGAAAAGCAATTAGCCAAAGCAATTAATACCAGTTCAGTTAGCCAACGATTCAAACACTCTNNNNNNNNNNNNNATTCCAAGCGTATTCGGCGGCCGAAGGACTAACGTCTTCGACCCATTCTCTCTCGACATCTGGGATCCATTCCAGGACTTCCCGTTGATCAGCGGCGGTAGCAACGCAGCCTTGTCAGGCCCGCGGTCGGAGCTGGCGAGCGAGACGGCGGCGGTGGCGAACACGAGGATCGACTGGAAGGAGACGCCGGAGGCGCACGTGTTCAAGGCGGACCTTCCGGGGCTGAAGAAGGAGGAGGTGAAGGTGGAGGTTGAGGAAGGAAGGGTGCTGCAGATCAGCGGAGCGAGGAGCAGAGAGAAGGAGGAGAAGAACGACAAGTGGCACAGGGTGGAGAGGAGCAGCGGCAATTTCCTGCGGCGGTTCAGGCTGCCTGAGAACGCGAAGGTGGATAGGGTGAAGGCTAGTTTGGAGAACGGGGTGTTGACTGTGACTGTGCCCAAAGAGGAGGTGAAGAAGCCTGAAGTTAAGGCCGTTGCGATTTCTGGCTAA

>PmsHsp19

ATGGCTCTCAGCATCTTTGGTGGCCGACGAAGCAACGTCTTCGATCCGTTTTCTCTTGACATCTGGGACCCCTTTGAGGGCCTTGGCACTCTGGCCAACATCCCACCCTCTGCTCGCGAAACGACTGCCATCGCCAGCACTCGCATTGACTGGAAAGAGACCCCAGAAGCCCACATCTTCATAGCTGACCTCCCGGGGTTGAAAAAGGAGGAAGTGAAAGTTGAGGTTGATGACGGGAAGGTGCTTCAGATCAGCGGAGAGAGGAGGAGAGAGCAGGAGGAGAAGAACGACAAATGGCACAGGATTGAGAGGAGCACCGGCAAGTTTTCGAGGATGTTCAGGTTGCCGGAGAATGCGAAAATTGATCAGGTCAAAGCTAGCATGGAGAATGGGGTTCTCACCGTGACTGTGCCCAAGGAGGAGGAGAAGCGGCCACAGGTCAAGGCCATTGACATATCTGGCTAA

>PmsHsp22

ATGGAAACCAAGGTTGCAGCAAACGATGAGCAGTGTTACGAGGTCTTTGAACCGTTTTGCCGGTGGAAGAAAGAGGAAGGACTTGACATTCTTGAGGTTCATCTACCAGGTACTCCTAGATCATGTTGATTCTTGTCTGAATATGTAAGCATTTGACTAACATAAGTGCGACAACAGAACATTATGTTCAGCGGGTTACATTCCATGTCACGTTTCTCTTTTATTTTGTTTTACTTGTTGATTCATGTAGGTTTCAAAAGACAAGATGTCAGAGTTCAAATGAACAACAAGGGCATCCTAACCATTAGTGGAAAGCAATCTATGGAAGAAGAAACTGCATCTCCACCCAGCCGCTTCCTCAAAGAGATCAAAATTTCTACAAATTGTAATACGAGTGGAATCCGTGCTAAGTTTTCGCATGGAATTCTTTCCATATCTATGCCTAAGAAAGTGCCAAACCTTTCAACACAATTATCAGGCAGCGGAGACAAGATTAAGGCTGCTGAAATTGCTACATGGTCGGCAATTAATTACTATTTACTTGGTTTAAGAAGCAAAATTTTAAGCAAGGATATGGTTCTGAAGATGGCGGGGGTCGCTCTGGGAATGGCTCTTGGAGGTTATGCAATATATAGATATCCAAAGTCAGCTTGTGTCCAAAACTGA

>PmsHsp11

ATGGACGTGAGAATCGCGGGTTTGGATTCCCCACTCTTCTCTACACTGCAGCACATCATGGACTTCAACGACGAGCCTGACAAATCGTTCAACGCCCCCACCCGCACCTACGTCCGCGACGCCAAGGCCATGGCCTCCACGCCGGCGGACGTCAAGGAGTATCCCAACTCCTATGTCTTCGTCGTGGACATGCCAGGCCTCAAGTCCGGAGACATCAAGGTCCAGGTGGAGGACGATAATGTGCTTGTGATCTCCGGCGAGAGGAAGAGGGAGGAGGAGAAGGAGGGGGCTAAGTATGTCAGGATGGAGAGGAGGGTCGGCAAGTTTATGAGGAAGTTTGTGCTGCCTGAGAATGCCAATCTAGAAGCCATTTCTGCTGTTTGCCAAGATGGGGTCCTGACTGTGACTGTGGAGAAGCTGCCTCCGCCTGAGCCAAAGAAGCCCAAGACCATTGAGGTCAAGATTGCTTGA

>PmsHsp12

ATGAGTATGGAGGTGAGCCTGAGAAACATGGGGTTCGAGCCAAACCTGCTCGAGACTCTGCACGACCTACTGGACTTCTCCGACGAACAGAACCAGTCCAGCCACCATGCGCCTTCGCGCCAGTACGTGCGCGAGGCCAAGGCCATGGCTGCCACGCCAGCCGACATCAAGGAGACTCAAAACGCCTATATTTTTGTTGTGGACGTGCCCGGGCTGAGGCCGGATATGGTCAACGTTCAAGTTGAGGACGACAACGTGCTGGTGGTGAGCGGGGAGAGGAGGAGAGAAAAGGAGAAGGATCAGGGTATTAAGTATTTGAGGCTGGAGAGGAGGCTTGGCAAGTACCTCAAGAAGTTTGTGCTGCCTGAAAATGCTGATATTGAGAAGATCTCTGCCGAGTGTCAGGACGGGGTATTGACTGTGAGTGTGGCGAAGAAGCCGCCGCCTGAGCCTAAGAAGCCCAAGACTGTTCAGGTCCAAATAAGCAGCGGCCAAGGCAGCGGTCAGGGCAAACAAATCGGCCAAGGCGGTGGTCAGGGTGGCGAAGGAGGACATGGAGGTGGTCAGGGCGGCGAAGGAGGACATGGAGGTGATCAAGGCGGCGGGCAGGGAGGCCAAGGTGGCGGACAAGGGGGCCAACATGGCAGTCAAGGTGGACAAGGTGGGGGCCAAGACAGGTAA

>PmsHsp13

ATGGATTTGAGAAACGCGGGCAATGTCTTCGCGTTCTTGGAAGACATGCTAGACTTGGCAGAGCAAGAGCCCGAAAAGCCCCGAAACAACAACCACCCATCTCGGGCGTACGTCCGAGACGCGAAAGCGATGGCGGCGACGCCGGCGGACGTGGTGGAGTACCCGAACGCGTACGTGTTCGTGGTGGACATGCCGGGAATCGAGGCGGGACAAATTAAAGTGCAGGTGGAGAACGACAACGTTTTGGTGCTGAGCGGAAAGCGAAGGCGGGAGGAAGGGATCAAGGAGAGTGGGGTGAAGTACGTGAGGATGGAGAGGAGGGTTGGGAAGTTCATGAGGAAGTTTGTGCTGCCGGAGAATGCCAATTTGGATGCGATTTCGGCGGTGGCTAAAGATGGGGTTCTCACTGTGAGGGTGGAGAAGCTGCCTCCGCCGGAGCCCAAGAGGCCCAAGACCATCCACGTCAATGTTGCTTGA

>PmsHsp17

ATGAGCAGAGTAGCCGATTCGAGTGCCTTCAATGGGGATTTTGCAACAGCAGTGAACCACCTGCTCAATTTCCCTGAAACCATTGACAAGTTCATGCTCCCTTCTCGGGCTCACGAAACCAACAACGAGAACAAAGGAGCGGCTAGCATTCCAGTGGACATTTTGGACTCTCCCAAGGAGTATATATTTTTCTTGGACTTGCCTGGCTTGTCCAAATCTGATATTCAGGTAATTTCCCACTTTCCCACTTGTCCAAATCTGACCTATTTTCTACTTCCTCTCCTGAAATTCTCAGTCTTTCGGAGAACGAATTGTGTGAGTGGTTGGTTTTTTATTTTTGTACATAAATTTTTCATGGTTTTTGTTGATGAATTATGTGAGTGGTAACAATTTTGTGGTTGCAATGCAGGTAACAGTTGAAGATGAAAATACTCTGGTGATCCGAAGCAATGGGAAGAGGAAACGTGAAGATGGGGAGGAGGAAGGCTGCAAGTACCTGAGGCTTGAGAGGAGAGGACCCCAGAAGCTGTTGAGGAAGTTTAGGTTGCCCCAAAATGCTAATGTAGGAGCCATATCTGCCAAATGCGAAAATGGGGTTCTCACTGTGGTTGTTGAGAAGCTTCCTCCACCTCCCAAGCCCAAAACAGTTGAAGTTTCTATCTCTTGA

>PmsHsp10

ATGCCTAGGAGGATAGTGATGAGGATGGTGGCCTTCTTAGGCCTCCTCCTCATGCTCATGGCCACCACCATCAAAACCCATGCCTTAGTTCCATACCCAACAAGACCATCATCACTAAGTCTATGGGACATGACCGATGACCCCTTCAGAATTCTCGAACAAACCCCCTTCACCATCCCCATAGACGTCTCAGCCGCCGCCCTCCAAGACACGCTCGCTTTGGCACGTGCCGACTGGAAGGAGACGGCCACTGCGCACGTGATCACATTGGACATTCCGGGGATGAAGAAGGAGGACGTGAAGATAGAGGTGGAGGAGAACAGGGTGCTCAGGATCAGCGGAGAAAGGAAGATGGAGAAACAAGGGGAGGGTGACAAGTGGCACAGAGCTGAGAGGACAAATGGCAAGTTCTGGAGGCAGTTCAGGCTGCCTGCGAATGCTGACGTGGATCAGATCAAGGCTCATCTTGAGGACGGGGTGCTCAGAATTACGGTGCCAAAGTTTGCTGCGGAGAAGAAAAGGCAGCCTAAGCTCATCGACATTGCTCAACACACTACTTCTGATGATGATGCGGATATCAAGGCTGCCAAGGTCGCATGA

>PmsHsp15

ATGGAGCTCCCAGCCTTCCACACTTACCAATATGTCTTCCCTTCTCATCTCCTTTATCCATATCACTTGGCCCCTGAAAACTACGTCCACTGGACTGAGACCCCAGAGTCTCACATTTTCTCTGCTGACCTCCCTGGTAAGTTTAAAAATCCCTCTTACTTTTTCTTTTATGGGTTTTTATGCAAGCGATAGTCTAAATAACTCTAAATTAATCTAATTTAGTAGGAGGCAATTCGAACTTGGGTGTAAGAAATGGGCTCACTGCCCTAATCCACTGGCATGACTCACGTCTGACCTCTTTTAGTCCTTTTTAATTCACCTTTCTTGTTATGTTTCCGTTGGAATGGTTATGAAGCTTTGTTTTTGGTTGTTGTACTATGCATATGAGCAGGTGTTAGGAAAGAGGAAATAAAAGTAGAAGTAGAGGATTCAATATACCTCATAATTCGAACACAGAGAATTGATGAAGCCACAGAGCCCAGCAGGAGCTTTATGAGGAAGTTTCGGATTCCAGGTAGGGTTGATCTCGAAAGGATTTCAGCTGGATATGAAGATGGAGTATTGACAGTCACAGTGCCAAGATCTTTAAGGAGGACTTTTTATATTGACCCAGCTGATGTCCCAGAACGGCTTGAAGTTCTTGCAAGGGCTGCTTGA

>PmsHsp24

ATGCTCTACTTTTCTCAAAAGTGCTTCTTCTCCCACACTATAAGAACCAACAAGCCTCAAACCTCTCTCCACCACACACTCAAAAGTTCAGTTTCTCTAATCTTCAATTCAATGACTACTACTCGCAAGCAGCTTGAAGTTCTAACAGATGATCAAACTCCACACAAATGGTGTGTTTTGTTGAGAGAAGATGTGTTCAAGAAGTTCATGTCTCAAGGCAGTCCAGCAGTGCACAAGGTTTTTGGTGGAGGATCATTGTTCAGTCCATTCTTGTTTGGGAAATTTTTTGATCCTTCTGATGCCTTCCCACTGTGGGAGTTTGATGCAGATATCTTGTTGGCTGGTCTCAGAAGCTCTGGTCAAAGCAGTACTGTTGACTGGTTTCAAACAGATCAAGATTATGTACTAAAAGCAGATCTACCAGGTAAGATTTCCAAATACTTCTAATATATATCTTAGTGATTCAAATCATCATGAACCAAGGAGACTGCATAGTTGAATTGTGTATGTTATTTTGGCAGGCTGCAATATACAAACCTAAGAAATATAGGTGGTATTATTCCTTAATTCACAAGTAAACCTTTTGAGTTTTGAGAGACAAGGAAAGGAGGCCTTTTTATCAAACATTTATGTTGGGTTTTGTTGCTTTTGGTAATTGAACATTTAATGTTGGGCTCCAAATGGGTCTTTTTTTAGTACAAGCAATAGTCTAAACTAGAGGAAAGGAGATTTTTCGCACACACACACAAATGATGTCATGGGAGTTCGAACTTAAGACCCTTTTCCACTGGGCTAGACCCCGTTAGTAAAATGGATCTTTTTAAAATCTGATACATCATATGGGATCAAAAATCATAACTTCATCTTAGTCCAAATCAGTTTCCTTGCCCCCTCACCACCTAACTAGCCCAATAGGCTTATTTATAAAGAGTTTTGAACTATAAACTAAAAATTCAAGAGTGCAATGAAACAGAACAGTATTTTTTATTTGGTGAAACAGGAGATGGGAAAAACAATGTTCAGGTCTATGCAGAAAATGGGAAGGTTGTGGAAATTAGTGGGCAGTGGAAGCAGCAAGGAGGAGAGTCCAAGAGCACAAAAGATTGGAGAAGTGGGAATTGGTGGGAACATGGGTATGTTAGGAAGCTTGAGCTCCCACAAGATGCAGATTGGAGAAGAATAGAGGCCTCTGTGACTAATGACCTACTTTTAGAAATCAAAATCCACAAGATCAACCCCTTGGATTGTGATATTAGTCATCTGACCCTGAAAGATAAGGAAGCAGTGTAA

>PmsHsp14

ATGTCTCAGGCCGTATCGAATTTGAGCATTTTTCTTCCAATGTCATCTGGGAGGAGGACCAAAAATTGCCCATCCCCTGTTTTCTCAAAACCAGTTAAGAACAGCCTCAGGGCCATGGCAAGAGATGCAAGGGACAACCTTGACCACTTGCAGAGGGCCACCACCAAGCACCAACAACAACCACCACCCCAGCCCAAAAAGAGAGTCGCGCCGGCACCACCTGTAGGTAAATTAATCACCCACTTATCCAAATTTGAAAATCTCACGTTAAAGTTAGAAACTTTAATTTGATATTGATAAATAAACAAAAACCATTGAATTTTTGCAGGGTTGTGGGACCGGTTTCCGACGGCAAGGACAGTTCAGCAGATGATGGAGACCATGGAGAGGATGATGGACGACCCGTTGGCCTACTCGGGCGGGTCGGGTTGGGCATCCCCATTGCCGACGGAGACAGGCGGCTACAGCAGGGGAAGGACCCCCTGGGAGATCAAAGAAGGTGAGGCTGATTACAAGATGAGATTTGACATGCCTGGGATGACCAAAGAGGACGTCAAGGTGTGGGTTGAGGAGAAAATGCTGGTTGTGAAGGCAGAGAAGGTGACCAAGAAGAAGGAAAATGGGGTGCAAGAAGAAGAAGATAATGGTGATGATGAGTGGTCTGCTAAGAGCTATGGAAGGTACAGCAGTAGAATTGCTTTGCCTGAGAATATTCAGTTTGAGAAGATTAAGGCTGAGGTTAAAGATGGGGTCTTGTATATTACTATTCCTAAGGCTACCAGTAGTTCTAAGATTTTGGACATTCATGTAGGGTGA

>PmsHsp20

ATGTCTTCAGCTTTGGCTTTGTCATCTTCATCACCTTTGCTATCAACCAAAGCTAGGTCTTCAATCAAAACATATGTCACTGCGCCGTGCTCAGCCACCTTTCCTTCGCGGTTGCATAGGCTGCCCGTGGTGAGAGCTCAGGCTGGTGGAGATGGCAAGCTGGACGTGCAAGTCAATCAGGGCAACCAAGGAACTGAAGTCGAGAGGAGGCCAAGGAGGTTGGCTGGCGACATTTCACCTTTTGGTAAGTGTCACAATCTCTCGTAAGTTGTTCTTTACTTGAATTCTGACCTGTTTGAACAGAGGAAACAGAGGAAAATTAGACAGGAGGTTCTTAATGTTTATTTAGTGGTTTCTTTGTTTCTTTAAAAAGCACCATCAAACTTGTTTGCTTAAAGATGAGTTTCAATTTGTTTTCAGGTTTATTGGATCCCATCTCCCCAGTGAGGACCATGCGCCAAATGCTGGACACGGTGGACAGGCTCCTGGAGGACACTGTGACATTTCCAGGCAGAAACAGAGCATCAGGGGAAGTACGTGCACCTTGGGACATCAAAGATGATGAACATGAGATCAAAATGAGGTTTGACATGCCCGGCCTCTCCAAGGAGGATGTCAAGGTGGCTGTAGAAGATGATGTTCTTGTTATAAAGGGAGAGCACAAAAAGGAAGAGAGTGGTGATGATTCATGGTCGAGCAGGAGCTTTAGCTCCTATAATACCCGCCTTCAGCTTCCTGATAATTGTGAGAAGGACAACATAAAGGCAGAGCTTAAGAACGGTGTTCTTTACATATCCATTCCCAAAACTAAAGTTGAACGCAAGGTCATTGATGTTGCGATCCAGTGA

>PmsHsp21

ATGGCTTCTTCAGTTTCGATCCTTCTCAGAAGGGCCTCAGCCCCAACCCTCTTCTCCAAGCTCTCCAGCCCTATTCGCTCTGCTTCAGTTTCTCCCCTTGTCTCTCGCTCCTTCAACTCCAACGCCCAGGTCACAAGCTACGATCAAGATGATCGTAGTGTCCCTGTTGATCGCAGCACCACCGACAGGTCTCCCTCTCGCCGTCGTGACTTTGGCCCCACCTTCTTCTCAGGTACTGGTCATCTGGGCTCGTTTCATATCTTTTTTTGGCATAATATTGAGGGTTATCTCAATTGGGGTTTTTTTCTCCTGGTCAGATGTTTCGAAGTCCCAACTGGGTTTTTCGTTTTAGTCAAATATCTCAATTGGGTTTTTGTGTTTTTGGGAATTGCAGATGTGTTTGATCCATTTTCACCAACAAGGAGTCTGAGCCAGGTTCTGAACATGATGGACCAGTTCATGGAGAACCCATTTCTTGCAGGGTCCAGAAGAGGCTGGGACGTGAAGGAAAACGGGGAAGCTCTGTTTCTGCGGATGGACATGCCAGGCTTAGACAAAGAGGATGTGAAGATCTCGGTGGAGCAGAACACGCTGGTTGTGAAAGGAGAAGATAAAGACTCGGAGGATGAAGAAGGTGGAGGCAGGAGGTTCTCTAGCAGATTGGATCTGCCTCCCAATCTTTACAAGCTCGATTCGATTAGGGCTGAGATGAAGAACGGGGTTCTGAAGCTGGCGATTCCTAAGGTGAAAGAGGACGAGAGGAAGGACGTCTTTGAGGTTAAGGTCGAGTGA

>PmsHsp23

ATGGCTTTGGCGCGTTTGGCTTTGAAAAACTTGCACCAGAGGGTGCTTTCTCCAGCTTCTTTTTCTGCTGCTTCTGTGCTGGGGCATGGTGTTAATGAGAGGACTGCTGGTGAGAGCAGAGGCAGAGGTGGCAATGAGATTGTGAAGAGATTTAGTACAGAAGCTAATGAGAAGGTGTCTGGTGAGAAATCAGAGAACAAAGATGTTGCTGTTTCTCAAGGTAAAAGGTCTAGGTTGTTCCCTAGAAGGCAACGTAGGAGGGGACTTTGGAGGGACAGTGACAGAAACTTCGTTCCTGCTCTTTATGGTACTTTTTCACTCCCTTATAATCTAGATCCTTGATCATTTTAGACTTTAATCATGTTTAAACCTAATGATTTAATTAGGTTCAAGTGTACAATTTAATTATAGTATATTATGTTTTCAGACTATAAATCTGAATCATCGATTATTTGCACTCTTAAACCTGTGATTCAGATTGACAGTTTGACACGACTTTCAGTTTGACCCAAATGTGACTCTAACTAAAACTATTGAACAAGCAAAGCTCAAAGTGAGAATTAATTTGCTAACATATTCTTTTCTTCTTAGAATTCTTTCCCTCGGGCCTTGGAAATGCACTGGTGCAAGCAACAGAGAACATAAACAGGCTGCTTGACAACCTCAACATATCACCATGGTCACTCACCGGGCGTGTCAAAGAGAAAAGTGACAGCTACAAACTGCAGTATGATGTGCCAGGGCTTGCGAAGGAGGATGTGAAGATCATTGTTCATGATGGGTTTTTGGAAATCAAAGGAGAGTACAAAGAAGAGGAGGAAGAAGGATCGGAGGGTTGGAGATATGGCTACTATGACACCACCCTCCAGCTGCCTGATGATGCCAAAGTTGATGATATAAAGGCAGAGCTGAAGGATGGGGTTCTGACCATCACCATTCCTAGAACTGAGAAGCCAAAGAAGGATGTGAAGGAGGTGAACGTACAATGA
